# Cs_2_CO_3_-Initiated Trifluoro-Methylation of Chalcones and Ketones for Practical Synthesis of Trifluoromethylated Tertiary Silyl Ethers

**DOI:** 10.3390/molecules22050769

**Published:** 2017-05-18

**Authors:** Cheng Dong, Xing-Feng Bai, Ji-Yuan Lv, Yu-Ming Cui, Jian Cao, Zhan-Jiang Zheng, Li-Wen Xu

**Affiliations:** Key Laboratory of Organosilicon Chemistry and Material Technology of Ministry of Education, Hangzhou Normal University, Hangzhou 311121, China; 2014110755@stu.hznu.edu.cn (C.D.); baixf@licp.cas.cn (X.-F.B.); 2015111018019@stu.hznu.edu.cn (J.-Y.L.); ym_cui@hznu.edu.cn (Y.-M.C.); caojian@hznu.edu.cn (J.C.)

**Keywords:** chalcone, trifluoromethylation, 1,2-addition, trifluoromethylated silyl ether

## Abstract

It was found that 1,2-trifluoromethylation reactions of ketones, enones, and aldehydes were easily accomplished using the Prakash reagent in the presence of catalytic amounts of cesium carbonate, which represents an experimentally convenient, atom-economic process for this anionic trifluoromethylation of non-enolisable aldehydes and ketones.

## 1. Introduction

The challenge to generate organofluorine molecules featuring a trifluoromethyl motif at a carbon center has increasingly stimulated high interest both in academic and chemical industry research [1,2]. In the past decades, trifluoromethylated compounds have received much attention because of their significant applications as important synthons, biologically active agents, and functional materials that exhibit specific and unique biological and physical features [3,4,5,6,7,8]. Recent reports on the direct introduction of a trifluoromethyl group by electrophilic, nucleophilic, or radical processes have revealed the equally challenging approach of exploiting prochiral trifluoromethylated substrates [4,9,10,11,12,13,14,15,16,17,18,19,20]. In addition, the chemoselective construction of trifluomethylated tertiary alcohols is undoubtedly one of the most fundamental topics in organofluorine chemistry and remains a highly useful process in organic transformations. For this purpose, a direct trifluoromethylation of carbonyl compounds, such as ketones, could be easily completed by nucleophilic addition of TMSCF_3_ to give trifluomethylated alcohols in the presence of a fluoride catalyst or other Lewis bases, including phosphines, amines, TBD, sodium or lithium acetates, etc., which mediate the silicon-carbon cleavage of TMSCF_3_ [21,22,23,24,25,26,27,28,29,30,31,32,33,34,35,36,37,38,39,40,41,42,43,44,45,46,47]. Notably, since the first report of Prakash and Olah [23] concerning the trifluoromethylation of benzaldehyde to give organofluorine compounds bearing secondary hydroxyl groups in the presence of fluoride ion reagent there has been a lot of effort devoted to the development of this type of trifluoromethylation reaction, including asymmetric versions of such transformations [22,23,24,25,26,27,28,29,30,31,32,33,34,35]. In this regard, despite the fact that there are several successful synthetic methods in the case of the trifluoromethlytion reactions with TMSCF_3_, the introduction of commercially available, simple and cheap bases as catalyst precursors for the establishment of a highly efficient and practical trifluomethylation reaction and corresponding one-pot synthesis of trifluoromethylated silyl ethers is still a highly desirable synthetic methodology target.

**Scheme 1:**
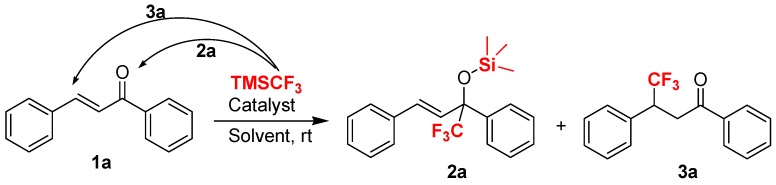
The development of new reaction conditions for the trifluoromethylation of chalcone: 1,2-addition versus 1,4-addition.

As part of our continuing interest in the catalytic construction of organofluorine molecules [48,49,50,51], we wanted to investigate trifluomethylation reactions of structurally diverse ketones. To evaluate the feasibility of a simple and cheap base-promoted trifluoromethylation in the absence of fluoride reagents, such as tetrabutylammonium fluoride (TBAF), we conducted preliminary experiments on the trifluoromethylation of chalcone **1a** with TMSCF_3_ as a model reaction. In theory, there are two possible pathway for the trifluoromethylation of chalcone with TMSCF_3_: 1,2-addition and 1,4-conjugate addition [52,53], respectively, in which the chalcone could be converted into two different organofluorine compounds bearing trifluoromethylated groups (Scheme 1).

## 2. Results and Discussion

Initially, we thought that cupric subcarbonate could be an effective catalyst in the trifluoromethylation of chalcone **1a** with TMSCF_3_ because of the similarity of its copper center and basic carbonate, which could possibly lead to an asymmetric transformation. Unfortunately, cupric subcarbonate has no activity in DCM in this reaction (Table 1, entry 1). Then we optimized the reaction conditions employing various bases to establish a possible copper-catalyzed trifluoro-methylation of chalcone with TMSCF_3_. After screening a variety of inorganic bases (Table 1, entries 2–12), we found the use of KHF_2_, KOH, *t*-BuOK, or Cs_2_CO_3_ led to the formation of only product **2a** in moderate yield (52–60%) without the formation of 1,4-adduct **3a**. Interestingly, the trifluoro-methylated silyl ether **2a** was obtained in high yield (94%) in the absence of cupric subcarbonate, which revealed Cs_2_CO_3_ was a highly active catalyst in this reaction (Entry 13). Under similar conditions, we found that fluorides and KOAc did not work well in term of the direct synthesis of trifluoromethylated silyl ethers (Entries 14–16 and 18). In addition, we found that K_2_CO_3_ gave an inferior yield (only 72% isolated yield of **2a**) in this reaction, in comparison to that obtained with Cs_2_CO_3_ (Entry 17). Other catalysts, such as KOH and *t*BuOK, provided the desired product in 83% and 71% yield, respectively (Entries 19 and 20).

In order to investigate or optimize the reactions, other solvents were examined. It seems that the trifluoromethylation of chalcone has a strong solvent effect. DCM was found to be suitable and the best solvent for this transformation in comparison with others (Table 2, entries 2–9). Notably, when THF was used, although the trifluoromethylation of chalcone occurs smoothly to give high conversion, the chemoselectivity is not good because of the low ratio of silyl ethers **2a** and desilylated **4a** (Table 2, entry 3, **2a**/**4a** = 36/51). While DMF and DMA was used as solvent, the desilylated trifluoromethylcarbinol could be obtained as the solely product in good yields (Entries 10–11). Notably, the use of Cs_2_CO_3_ in CH_2_Cl_2_ gave the desired trifluoromethlyated silyl ether **2a** without the formation of desilylated product **4a**, which exhibited superior chemoselectivity than that in DMF/DMAc for this reaction.

**Scheme 2:**
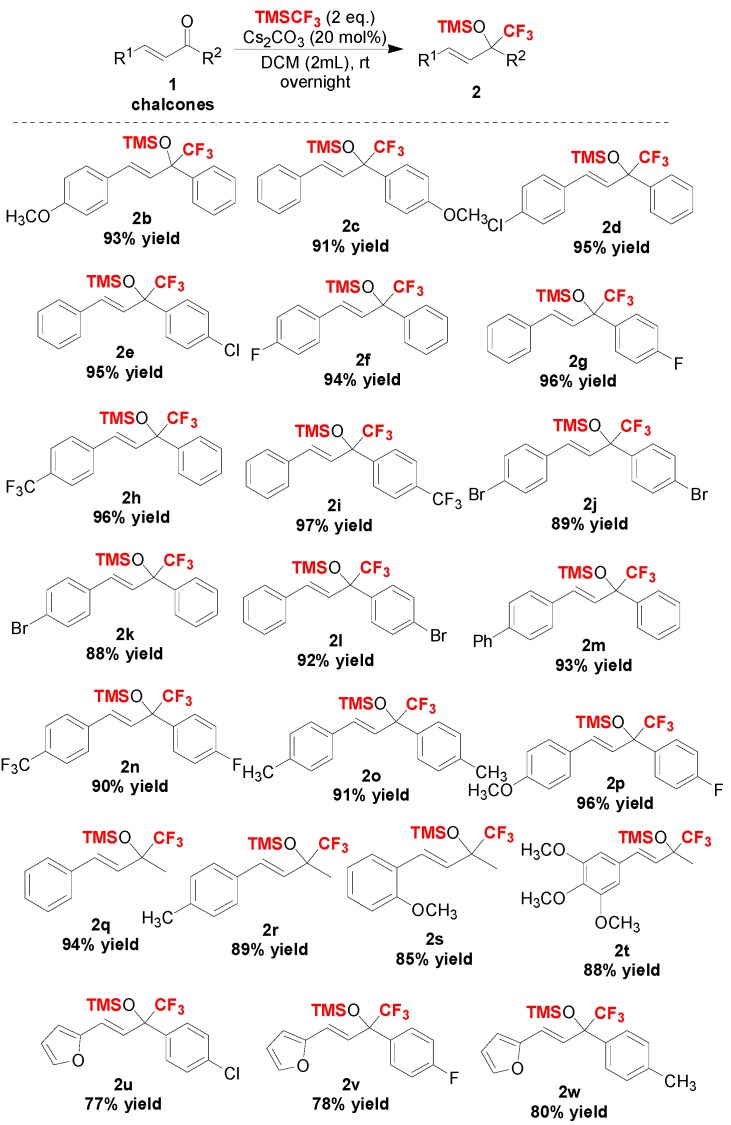
Scope of chalcones tested in this trifluoromethylation reaction.

**Scheme 3:**
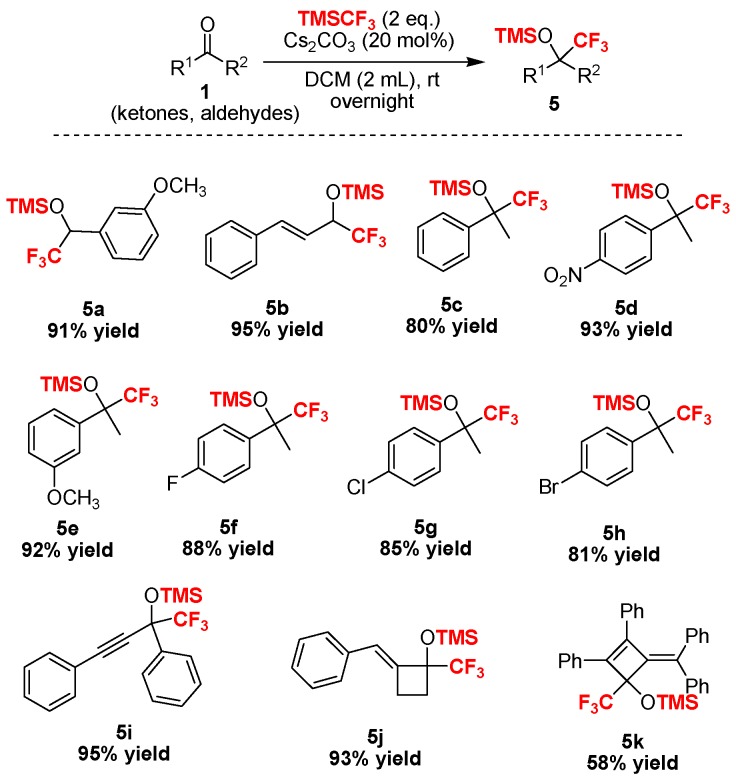
Substrate scope of ketones and aldehydes tested in this trifluoromethylation reaction.

With the optimized reaction conditions in hand (Table 2, entry 1), we further examined the scope of the reaction, and the experimental results are summarized in Scheme 2 and Scheme 3, respectively. In the first part, we demonstrated the general applicability of the Cs_2_CO_3_ catalyst in the trifluoromethylation of more than 22 different chalcones (Scheme 2). The results showed that chalcones, including alkyl, aryl, halogen or other substituents on the phenyl moiety showed good to excellent conversion, affording the corresponding trifluoromethylated silyl ethers in high yields (Scheme 2). Moreover, heterocylic enones and alkyl enones were also suitable substrates in this reaction (Scheme 3).

**Figure 1:**
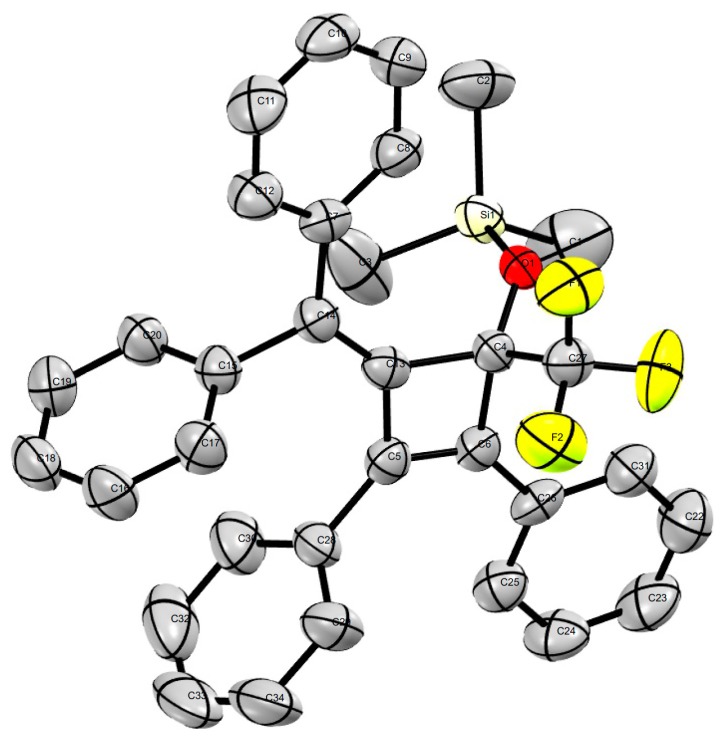
Crystal structure of **5k** (CCDC 1487785).

In addition, we found that a broad range of aldehydes and ketones bearing alkynyl and sterically demanding bulky substituents were fully converted into the trifluoromethylated silyl ethers without any evidence of the undesired desilylated byproducts, which illustrated the superior selectivity of this procedure in comparison with fluoride ion-promoted trifluoromethylation. Notably, we recently found that the alkylidenecyclobutenone **1k** [54] was a highly reactive compound and could be easily used in ring-opening and ring expansion with Grignard reagents, organolithium species, primary amines, and water [55], in which the four-membered ring was easily broken by the nucleophilic reagent. Interestingly, we found that the four-membered ring was stable in the trifluoromethylation reaction [56] and the desired products **5j** or **5k** were isolated in promising yields. The structure of the novel compound **5k** was unambiguously confirmed by X-ray diffraction analysis (Figure 1). Therefore, this Cs_2_CO_3_–initiated trifluoromethylation described can be performed successfully without the need for harsh reaction conditions.

**Scheme 4:**
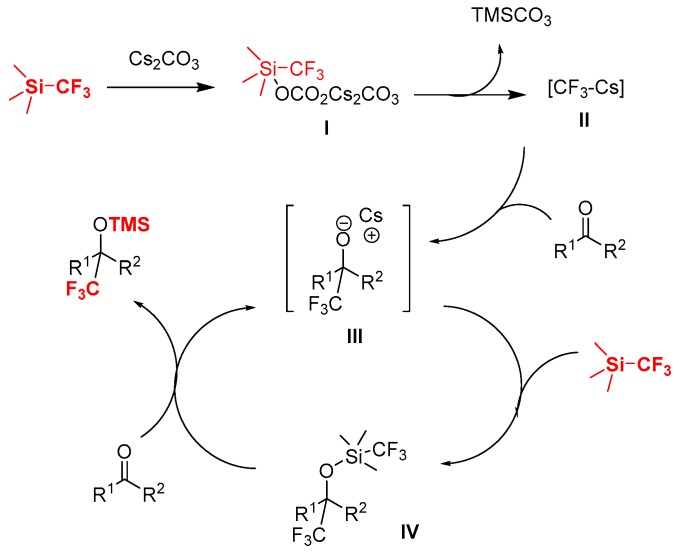
Possible mechanism for the Cs_2_CO_3_-catalyzed trifluoromethylation of ketones with TMSCF_3_.

From a mechanistic standpoint, as shown in Scheme 4, it is reasonable to consider that the initiation by carbonate anion of Cs_2_CO_3_ leads to the formation of the possible hypervalent silicon complex [3,23,57,58,59,60,61,62] to give trifluoromethylated nucleophilic reagent **II**, which then reacted with the ketone to give the reactive, and more nucleophilic alkoxide **III**, and the reaction between **III** and TMSCF_3_ leads to the pentavalent complex **IV,** then **IV** readily gives the product through CF_3_ transfer and *O*-silylation in the presence of ketone with regeneration of the catalyst **III**.

## 3. Materials and Methods

### 3.1. General Information

All solvents were purified by standard method. All reagents were received from commercial sources (Aldrich, Shanghai, China; Alfa Aesar, Shanghai, China; TCI, Shanghai, China). NMR spectra were recorded on 500 MHz or 400 MHz spectrometers (Bruker, Shanghai, China). Chemical shifts (δ) are reported in ppm relative to the signal of an internal TMS standard (δ 0.0) Coupling constants (*J*) are in Hertz (Hz). The following abbreviations were used to explain the multiplicities: s = singlet, d = doublet, t = triplet, q = quartet, m = multiplet, br = broad, Flash column chromatograph was carried out at medium pressure using 300–400 mesh silica gel (Qingdao Haiyang Chemical Co., Ltd., Qingdao, China). High-resolution mass spectrometry was performed on a JMS-700 MStation (FAB-MS and EI-MS, JEOL, Shanghai, China), 6520 Accurate Mass Q-TOFLC/MS (ESI-MS, Agilent, Shanghai, China) or EXACTIVE Plus (ESI-MS, Thermo Fisher Scientific, Shanghai, China) instrument.

### 3.2. Experimental Procedures

#### 3.2.1. General Procedure for Cs_2_CO_3_-Catalyzed Addition of TMSCF_3_ and Chalcone **1a** (Scheme 5)

A solution of chalcone (**1a**, 0.1040 g, 0.5 mmol) and TMSCF_3_ (0.1420 g, 1.0 mmol) in CH_2_Cl_2_ (2.0 mL) and Cs_2_CO_3_ (0.0325 g, 0.1 mmol) in a reaction tube were mixed. After stirring at r.t. overnight, once starting material was consumed (monitored by TLC), the mixture was purified by column chromatography (silica gel, petroleum ether/EtOAc = 50:1).

#### 3.2.2. Characterization of Compounds **2a**–**2w**

*(1,3-Diphenyl-1-trifluoromethyl-allyloxy)trimethylsilane* (**2a**). ^1^H-NMR (400 MHz, CDCl_3_) δ = 7.67–7.56 (m, 2H), 7.44–7.23 (m, 8H), 6.70 (d, *J* = 16.3 Hz, 1H), 6.56 (d, *J* = 16.4 Hz, 1H), 0.15 (s, 9H). ^13^C-NMR (100 MHz, CDCl_3_) δ = 136.1, 133.7, 133.3, 126.9, 126.4, 125.9, 124.9, 121.7, 78.2 (q, *J*_C-C-F_ = 28 Hz), 0.0. ^19^F-NMR (470 MHz, CDCl_3_) δ = −77.5 (s, 3F), HRMS (ESI) calcd for C_19_H_21_F_3_NaOSi [M + Na]^+^: 373.1206; found 373.1215.

*[3-(4-Methoxyphenyl)-1-phenyl-1-trifluoromethyl-allyloxy]trimethylsilane* (**2b**). ^1^H-NMR (400 MHz, CDCl_3_) δ = 7.60 (d, *J* = 7.3 Hz, 2H), 7.41–7.29 (m, 5H), 6.88 (d, *J* = 8.6 Hz, 2H), 6.60 (d, *J* = 16.3 Hz, 1H), 6.42 (d, *J* = 16.3 Hz, 1H), 3.81 (s, 3H), 0.11 (s, 9H). ^13^C-NMR (100 MHz, CDCl3) δ = 160.5, 138.1, 134.8, 128.0, 127.8, 124.6, 123.6, 114.2, 80.1 (q, *J*_C-C-F_ = 29 Hz), 55.2, 1.9. ^19^F-NMR (470 MHz, CDCl_3_) δ = −77.6 (s, 3F), HRMS (ESI) calcd for C_20_H_23_F_3_NaO_2_Si [M + Na]^+^: 403.1312; found 403.1325.

*[1-(4-Methoxyphenyl)-3-phenyl-1-trifluoromethylallyloxy]trimethylsilane* (**2c**). ^1^H-NMR (400 MHz, CDCl_3_) δ = 7.50 (d, *J* = 8.8 Hz, 2H), 7.44–7.27 (m, 5H), 6.95–6.85 (m, 2H), 6.69 (d, *J* = 16.3 Hz, 1H), 6.53 (d, *J* = 16.3 Hz, 1H), 3.83 (s, 3H), 0.13 (s, 9H). ^13^C-NMR (100 MHz, CDCl_3_) δ = 159.8, 135.8, 135.0, 130.0, 129.3, 128.8, 128.5, 127.1, 126.8, 126.5, 123.6, 113.3, 79.9 (q, *J*_C-C-F_ = 29 Hz), 55.2, 2.0. ^19^F-NMR (470 MHz, CDCl_3_) δ = −77.5 (s, 3F), HRMS (ESI) calcd for C_20_H_23_F_3_NaO_2_Si [M + Na]^+^: 403.1312; found 403.1320.

*[3-(4-Chlorophenyl)-1-phenyl-1-trifluoromethylallyloxy]trimethylsilane* (**2d**). ^1^H-NMR (400 MHz, CDCl_3_) δ = 7.62–7.52 (m, 2H), 7.45–7.28 (m, 7H), 6.65 (d, *J* = 16.3 Hz, 1H), 6.51 (d, *J* = 16.3 Hz, 1H), 0.18 (s, 9H). ^13^C-NMR (100 MHz, CDCl_3_) δ = 137.8, 136.2, 134.3, 134.2, 134.0, 133.7, 127.9, 127.6, 127.2, 126.1, 119.5, 80.0 (q, *J*_C-C-F_ = 28 Hz), 3.7, 3.4. ^19^F-NMR (470 MHz, CDCl_3_) δ = −77.2 (s, 3F), HRMS (ESI) calcd for C_19_H_20_ClF_3_NaOSi [M + Na]^+^: 407.0816; found 407.0832.

*[1-(4-Chlorophenyl)-3-phenyl-1-trifluoromethylallyloxy]trimethylsilane* (**2e**). ^1^H-NMR (400 MHz, CDCl_3_) δ = 7.53 (d, *J* = 8.6 Hz, 2H), 7.45–7.28 (m, 7H), 6.65 (d, *J* = 16.4 Hz, 1H), 6.53 (d, *J* = 16.4 Hz, 1H), 0.19 (s, 9H). ^13^C-NMR (100 MHz, CDCl_3_) δ = 136.8, 135.8, 135.7, 135.1, 134.8, 128.9, 128.6, 128.2, 127.7, 126.9, 126.7, 126.5, 126.4, 124.4, 79.9 (q, *J*_C-C-F_ = 28 Hz), 2.1. ^19^F-NMR (470 MHz, CDCl_3_) δ = −77.7 (s, 3F), HRMS (ESI) calcd for C_19_H_20_ClF_3_NaOSi [M + Na]^+^: 407.0816; found 407.0820.

*[3-(4-Fluorophenyl)-1-phenyl-1-trifluoromethylallyloxy]trimethylsilane* (**2f**). ^1^H-NMR (400 MHz, CDCl_3_) δ = 7.64–7.54 (m, 2H), 7.44–7.33 (m, 5H), 7.04 (dd, *J* = 12.0, 5.2 Hz, 2H), 6.65 (d, *J* = 16.3 Hz, 1H), 6.46 (d, *J* = 16.3 Hz, 1H), 0.14 (s, 9H). ^13^C-NMR (100MHz, CDCl_3_) δ = 164.1, 162.0, 161.7, 137.9, 137.1, 133.9, 128.3, 127.8, 126.1, 115.8 (d, *J*_C-F_ = 88 Hz), 80.1 (q, *J*_C-C-F_ = 29 Hz), 1.9. ^19^F-NMR (470 MHz, CDCl_3_) δ = −77.3 (s, 3F), −112.7 (s, 1F), HRMS (ESI) calcd for C_19_H_20_F_4_NaOSi [M + Na]^+^: 391.1112; found 391.1121.

*[1-(4-Fluorophenyl)-3-phenyl-1-trifluoromethylallyloxy]trimethylsilane* (**2g**). ^1^H-NMR (400 MHz, CDCl_3_) δ = 7.57 (dd, *J* = 8.6, 5.5 Hz, 2H), 7.45–7.27 (m, 5H), 7.07 (t, *J* = 8.7 Hz, 2H), 6.67 (d, *J* = 16.4 Hz, 1H), 6.54 (d, *J* = 16.4 Hz, 1H), 0.15 (s, 9H). ^13^C-NMR (100 MHz, CDCl_3_) δ = 164.3, 163.6, 161.8, 135.7, 130.0, 128.9, 126.9, 115.0 (d, *J*_C-F_ = 88 Hz), 79.9 (q, J_C-C-F_ = 29 Hz), 2.1. ^19^F-NMR (470 MHz, CDCl_3_) δ = −77.8 (s, 3F), −113.7 (s, 1F), HRMS (ESI) calcd for C_19_H_20_F_4_NaOSi [M + Na]^+^: 391.1112; found 391.1131.

*Trimethyl-[1-phenyl-1-trifluoromethyl-3-(4-trifluoromethylphenyl)allyloxy]silane* (**2h**). ^1^H-NMR (400 MHz, CDCl_3_) δ = 7.65–7.47 (m, 6H), 7.39 (dt, *J* = 7.2, 2.2 Hz, 3H), 6.76 (d, *J* = 16.3 Hz, 1H), 6.63 (d, *J* = 16.3 Hz, 1H), 0.14 (s, 9H). ^13^C-NMR (100 MHz, CDCl_3_) δ = 139.0, 138.9, 137.5, 137.4, 133.0, 126.8, 126.7, 125.6, 125.4, 123.1 (q, *J*_C-F_ = 312 Hz), 122.3, 79.9 (q, *J*_C-C-F_ = 27 Hz), 1.7. ^19^F-NMR (470 MHz, CDCl_3_) δ = −62.6 (s, 3F), −76.9 (s, 3F), HRMS (ESI) calcd for C_20_H_20_F_6_NaOSi [M + Na]^+^: 441.1080; found 441.1088.

*Trimethyl-[3-phenyl-1-trifluoromethyl-1-(4-trifluoromethylphenyl)allyloxy]silane* (**2i**). ^1^H-NMR (400 MHz, CDCl_3_) δ = 7.74 (d, *J* = 8.3 Hz, 2H), 7.66 (d, *J* = 8.4 Hz, 2H), 7.43–7.29 (m, 5H), 6.64 (d, *J* = 16.4 Hz, 1H), 6.55 (d, *J* = 16.4 Hz, 1H), 0.17 (s, 9H). ^13^C-NMR (100MHz, CDCl_3_) δ = 142.1, 135.9, 135.1, 128.9, 128.6, 126.9, 126.42, 124.8, 124.1, 122.9, 80.0 (q, *J*_C-C-F_ = 28 Hz), 2.0. ^19^F-NMR (470 MHz, CDCl_3_) δ = −62.6 (s, 3F), −77.6 (s, 3F), HRMS (ESI) calcd for C_20_H_20_F_6_NaOSi [M + Na]^+^: 441.1080; found 441.1069.

*[1,3-Bis-(4-bromophenyl)-1-trifluoromethylallyloxy]trimethylsilane* (**2j**). ^1^H-NMR (400 MHz, CDCl_3_) δ = 7.56–7.42 (m, 6H), 7.28–7.22 (m, 2H), 6.59 (d, *J* = 16.4 Hz, 1H), 6.50 (d, *J* = 16.4 Hz, 1H), 0.14 (s, 9H). ^13^C-NMR (100 MHz, CDCl_3_) δ = 137.0, 136.9, 134.4, 132.1, 131.2, 129.7, 128.3, 127.2, 123.1 (q, *J*_C-F_ = 108 Hz), 79.9 (q, *J*_C-C-F_ = 29 Hz), 2.0. ^19^F-NMR (470 MHz, CDCl_3_) δ = −77.6 (s, 3F), HRMS (ESI) calcd for C_19_H_19_Br_2_F_3_NaOSi [M + Na]^+^: 528.9416; found 528.9438.

*[3-(4-Bromophenyl)-1-phenyl-1-trifluoromethylallyloxy]trimethylsilane* (**2k**). ^1^H-NMR (400 MHz, CDCl_3_) δ = 7.62–7.54 (m, 2H), 7.51–7.45 (m, 2H), 7.43–7.35 (m, 3H), 7.29–7.24 (m, 2H), 6.64 (d, *J* = 16.3 Hz, 1H), 6.53 (d, *J* = 16.3 Hz, 1H), 0.13 (s, 9H). ^13^C-NMR (100 MHz, CDCl_3_) δ = 137.7, 133.7, 131.8, 130.0, 127.7, 126.3 126.1, 126.0, 125.7, 125.9, 120.6, 80.0 (q, *J*_C-C-F_ = 31 Hz), 1.8. ^19^F-NMR (470 MHz, CDCl_3_) δ = −77.2 (s, 3F), HRMS (ESI) calcd for C_19_H_20_BrF_3_NaOSi [M + Na]^+^: 451.0311; found 451.0322.

*[1-(4-Bromophenyl)-3-phenyl-1-trifluoromethylallyloxy]trimethylsilane* (**2l**). ^1^H-NMR (400 MHz, CDCl_3_) δ = 7.58–7.44 (m, 4H), 7.36 (dt, *J* = 12.1, 7.5 Hz, 5H), 6.64 (d, *J* = 16.4 Hz, 1H), 6.52 (d, *J* = 16.4 Hz, 1H), 0.15 (s, 9H). ^13^C-NMR (100 MHz, CDCl_3_) δ = 137.1, 135.6, 133.4, 129.1, 127.7, 126.8, 124.8, 124.3, 122.8, 79.8 (q, *J*_C-C-F_ = 27 Hz), 1.9. ^19^F-NMR (470 MHz, CDCl_3_) δ = −77.7 (s, 3F), HRMS (ESI) calcd for C_19_H_20_BrF_3_NaOSi [M + Na]^+^: 451.0311; found 451.0330.

*(3-Biphenyl-4-yl-1-phenyl-1-trifluoromethylallyloxy)trimethylsilane* (**2m**). ^1^H-NMR (400 MHz, CDCl_3_) δ = 7.65–7.56 (m, 6H), 7.51–7.33 (m, 8H), 6.73 (d, *J* = 16.3 Hz, 1H), 6.60 (d, *J* = 16.4 Hz, 1H), 0.17 (s, 9H). ^13^C-NMR (100 MHz, CDCl_3_) δ = 139.5, 138.4, 136.0, 132.7, 126.7, 126.5, 125.9, 125.5, 125.2, 124.9, 78.1 (q, *J*_C-C-F_ = 22 Hz), 0.1. ^19^F-NMR (470 MHz, CDCl_3_) δ = −77.3 (s, 3F), HRMS (ESI) calcd for C_25_H_25_F_3_NaOSi [M + Na]^+^: 449.1519; found 449.1528.

*[1-(4-Fluorophenyl)-1-trifluoromethyl-3-(4-trifluoromethylphenyl)allyloxy]trimethylsilane* (**2n**). ^1^H-NMR (400 MHz, CDCl_3_) δ = 7.62 (d, *J* = 8.2 Hz, 2H), 7.58–7.48 (m, 4H), 7.13–7.04 (m, 2H), 6.73 (d, *J* = 16.4 Hz, 1H), 6.61 (d, *J* = 16.3 Hz, 1H), 0.14 (s, 9H). ^13^C-NMR (100 MHz, CDCl_3_) δ = 162.2, 159.7, 137.0, 137.0, 131.7, 128.8, 128.6, 128.0, 127.9, 125.0, 123.9 (q, *J*_C-F_ = 16 Hz), 113.1, 113.0, 112.9, 77.7 (q, *J*_C-C-F_ = 29 Hz), 0.0. ^19^F-NMR (470 MHz, CDCl_3_) δ = −62.7 (s, 3F), −77.4 (s, 3F), −113.3 (s, 1F), HRMS (ESI) calcd for C_20_H_19_F_7_NaOSi [M + Na]^+^: 459.0986; found 459.0989.

*(1,3-Di-p-tolyl-1-trifluoromethylallyloxy)trimethylsilane* (**2o**). ^1^H-NMR (400 MHz, CDCl_3_) δ = 7.47 (d, *J* = 8.1 Hz, 2H), 7.30 (d, *J* = 8.1 Hz, 2H), 7.17 (dd, *J* = 13.2, 8.1 Hz, 4H), 6.65 (d, *J* = 16.3 Hz, 1H), 6.49 (d, *J* = 16.3 Hz, 1H), 2.36 (d, *J* = 10.7 Hz, 6H), 0.13 (s, 9H). ^13^C-NMR (100 MHz, CDCl_3_) δ = 136.5, 136.2, 133.1, 131.0, 127.4, 126.5, 125.9, 124.7, 123.9, 78.0 (q, *J*_C-C-F_ = 24 Hz), 19.1, 0.0. ^19^F-NMR (470 MHz, CDCl_3_) δ = −77.8 (s, 3F), HRMS (ESI) calcd for C_21_H_25_F_3_NaOSi [M + Na]^+^: 401.1519; found 401.1525.

*[1-(4-Fluorophenyl)-3-(4-methoxyphenyl)-1-trifluoromethylallyloxy]trimethylsilane* (**2p**). ^1^H-NMR (400 MHz, CDCl_3_) δ = 7.57 (dd, *J* = 8.5, 5.5 Hz, 2H), 7.34 (d, *J* = 8.7 Hz, 2H), 7.13–7.02 (m, 2H), 6.89 (d, *J* = 8.7 Hz, 2H), 6.57 (d, *J* = 16.4 Hz, 1H), 6.40 (d, *J* = 16.4 Hz, 1H), 3.82 (s, 3H), 0.14 (s, 9H). ^13^C-NMR (100 MHz, CDCl_3_) δ = 162.0, 159.6, 158.2, 133.2, 132.0 (d, *J* = 3.1 Hz), 127.9, 126.19, 122.3, 112.7 (d, *J* = 84 Hz), 112.6, 112.3, 77.8 (q, *J*_C-C-F_ = 29 Hz), 53.3, 0.0. ^19^F-NMR (470 MHz, CDCl_3_) δ = −78.1 (s, 3F), −113.9 (s, 1F), HRMS (ESI) calcd for C_20_H_22_F_4_NaO_2_Si [M + Na]^+^: 421.1217; found 421.1227.

*Trimethyl-(1-methyl-3-phenyl-1-trifluoromethylallyloxy)silane* (**2q**) [23]. ^1^H-NMR (400 MHz, CDCl_3_) δ = 7.39–7.13 (m, 5H), 6.67(dd, *J* = 16.1, 2.1 Hz, 1H), 6.18 (d, *J* = 16.0 Hz, 1H), 1.51 (s, 3H), 0.12 (s, 9H). ^13^C-NMR (100 MHz, CDCl_3_) δ = 136.0, 132.6, 128.7, 128.3, 127.6, 126.8, 73.9 (q, *J*_C-C-F_ = 29 Hz), 21.7, 2.1. ^19^F-NMR (470 MHz, CDCl_3_) δ = −82.1 (s, 3F), HRMS (ESI) calcd for C_14_H_19_F_3_NaOSi [M + Na]^+^: 311.1049; found 311.1040.

*Trimethyl-(1-methyl-3-p-tolyl-1-trifluoromethylallyloxy)silane* (**2r**). ^1^H-NMR (500 MHz, CDCl_3_) δ = 7.37–7.29 (m, 2H), 6.90–6.81 (m, 2H), 6.67 (dd, *J* = 16.0, 7.1 Hz, 1H), 6.11 (dd, *J* = 16.0, 6.9 Hz, 1H), 3.81 (d, *J* = 7.2 Hz, 3H), 1.58 (s, 3H), 0.18 (s, 9H). ^13^C-NMR (125 MHz, CDCl_3_) δ = 159.7, 131.9, 128.6, 128.0, 126.5, 125.3, 124.2, 114.1, 76.0 (q, *J*_C-C-F_ = 24 Hz), 55.3, 21.6, 2.1. ^19^F-NMR (470 MHz, CDCl_3_) δ = −77.3 (s, 3F), HRMS (ESI) calcd for C_15_H_21_F_3_OSi [M + H]^+^: 303.1225; found 303.1229.

*[3-(2-Methoxyphenyl)-1-methyl-1-trifluoromethylallyloxy]trimethylsilane* (**2s**). ^1^H-NMR (500 MHz, CDCl_3_) δ= 7.43 (d, *J* = 7.6 Hz, 1H), 7.26 (d, *J* = 7.5 Hz, 1H), 7.09 (d, *J* = 16.3 Hz, 1H), 6.99–6.84 (m, 2H), 6.30 (d, *J* = 16.2 Hz, 1H), 3.84 (d, *J* = 2.9 Hz, 3H), 1.60 (s, 3H), 0.19 (s, 9H). ^13^C-NMR (125 MHz, CDCl_3_) δ = 157.1, 129.3, 127.7, 127.1, 126.5, 124.9, 124.3, 120.7, 111.0, 76.3 (q, *J*_C-C-F_ = 24 Hz), 55.4, 21.6, 2.1. ^19^F-NMR (470 MHz, CDCl_3_) δ = −82.2 (s, 3F), HRMS (ESI) calcd for C_15_H_21_F_3_NaO_2_Si [M + Na]^+^: 341.1155; found 341.1158.

*Trimethyl-[1-methyl-1-trifluoromethyl-3-(3,4,5-trimethoxyphenyl)allyloxy]silane* (**2t**). ^1^H-NMR (500 MHz, CDCl_3_) δ = 6.68 (d, *J* = 15.9 Hz, 1H), 6.64 (s, 2H), 6.16 (d, *J* = 15.9 Hz, 1H), 3.90 (s, 9H), 1.60 (s, 3H), 0.21 (s, 9H). ^13^C-NMR (125 MHz, CDCl_3_) δ = 153.4, 138.4, 132.5, 131.5, 127.0, 126.4, 124.1, 105.4, 103.9, 75.9 (q, *J*_C-C-F_ = 23 Hz), 60.8, 56.1, 21.6, 2.1. ^19^F-NMR (470 MHz, CDCl_3_) δ = −82.2 (s, 3F), HRMS (ESI) calcd for C_17_H_26_F_3_O_4_Si [M + H]^+^: 379.1547; found 379.1556.

*[1-(4-Chlorophenyl)-3-furan-2-yl-1-trifluoromethylallyloxy]trimethylsilane* (**2u**). ^1^H-NMR (400 MHz, CDCl_3_) δ = 7.51 (d, *J* = 8.6 Hz, 2H), 7.41 (d, *J* = 1.5 Hz, 1H), 7.39–7.33 (m, 2H), 6.45 (d, *J* = 7.1 Hz, 2H), 6.41 (dd, *J* = 3.3, 1.8 Hz, 1H), 6.33 (d, *J* = 3.3 Hz, 1H), 0.15 (s, 9H). ^13^C-NMR (100 MHz, CDCl_3_) δ = 149.2, 141.2, 134.5, 132.7, 127.4, 126.2, 122.7, 121.5, 109.6, 108.6, 77.5 (q, *J*_C-C-F_ = 29 Hz), 0.0. ^19^F-NMR (470 MHz, CDCl_3_) δ = −77.7 (s, 3F), HRMS (ESI) calcd for C_17_H_18_ClF_3_NaO_2_Si [M + Na]^+^: 397.0609; found 397.0627.

*[1-(4-Fluorophenyl)-3-furan-2-yl-1-trifluoromethylallyloxy]trimethylsilane* (**2v**). ^1^H-NMR (400 MHz, CDCl_3_) δ = 7.56 (dd, *J* = 8.5, 5.4 Hz, 2H), 7.39 (d, *J* = 1.6 Hz, 1H), 7.09–7.01 (m, 2H), 6.48 (d, *J* = 5.9 Hz, 2H), 6.39 (dd, *J* = 3.3, 1.8 Hz, 1H), 6.32 (d, *J* = 3.3 Hz, 1H), 0.15 (s, 9H). ^13^C-NMR (100 MHz, CDCl_3_) δ = 162.2, 159.7, 149.4, 141.2, 131.7, 127.9, 123.0, 121.5, 112.9 (d, *J*_C-F_ = 22 Hz), 109.6, 108.5, 77.6 (q, *J*_C-C-F_ = 29 Hz), 0.0. ^19^F-NMR (470 MHz, CDCl_3_) δ = −77.9 (s, 3F), −113.8 (s, 1F), HRMS (ESI) calcd for C_17_H_18_F_4_NaO_2_Si [M + Na]^+^: 381.0904; found 381.0915.

*(3-Furan-2-yl-1-p-tolyl-1-trifluoromethylallyloxy)trimethylsilane* (**2w**). ^1^H-NMR (400 MHz, CDCl_3_) δ = 7.46 (d, *J* = 8.1 Hz, 2H), 7.38 (d, *J* = 1.5 Hz, 1H), 7.18 (d, *J* = 8.1 Hz, 2H), 6.49 (d, *J* = 2.7 Hz, 2H), 6.38 (dd, *J* = 3.3, 1.8 Hz, 1H), 6.30 (d, *J* = 3.3 Hz, 1H), 2.36 (s, 3H), 0.13 (s, 9H). ^13^C-NMR (100 MHz, CDCl_3_) δ = 149.6, 141.0, 136.4, 132.8, 126.7, 125.89, 123.5, 121.1, 109.5, 108.1, 77.9 (q, *J*_C-C-F_ = 29Hz), 19.0, 0.0. ^19^F-NMR (470 MHz, CDCl_3_) δ = −77.5 (s, 3F), HRMS (ESI) calcd for C_18_H_21_F_3_NaO_2_Si [M + Na]^+^: 377.1155; found 377.1169.

#### 3.2.3. Characterization of Compounds **5a**–**5k**

The spectral data of compounds **5a**, **5b**, **5c**, **5d**, **5e**, **5g**, **5h** and **5i** matched the reported data in all respects [42,44,45,63].

*Trimethyl-[2,2,2-trifluoro-1-(4-fluorophenyl)-1-methylethoxy]silane* (**5f**). ^1^H-NMR (500 MHz, CDCl_3_) δ = 7.56 (dd, *J* = 8.3, 5.5 Hz, 2H), 7.13–7.02 (m, 2H), 1.85 (s, 3H), 0.19 (s, 9H). ^13^C-NMR (125 MHz, CDCl_3_) δ = 163.7, 161.8, 135.9, 128.6, 126.3, 124.0, 114.8, 114.7, 76.0 (q, *J*_C-C-F_ = 24 Hz), 22.6, 1.8. ^19^F-NMR (470 MHz, CDCl_3_) δ = −81.9 (s, 3F), −114.2 (s, 1F), HRMS (ESI) calcd for C_12_H_17_F_4_OSi [M + H]^+^: 281.0979; found 281.0983.

*(2-Benzylidene-1-trifluoromethylcyclobutoxy)trimethylsilane* (**5j**). ^1^H-NMR (500 MHz, CDCl_3_) δ = 7.30 (ddd, *J* = 26.9, 16.5, 6.9 Hz, 5H), 6.59 (d, *J* = 2.4 Hz, 1H), 2.89 (dt, *J* = 14.7, 5.9 Hz, 2H), 2.63 (ddd, *J* = 7.1, 5.8, 4.4 Hz, 1H), 2.37 (d, *J* = 10.5 Hz, 1H), 0.20 (s, 9H). ^13^C-NMR (125 MHz, CDCl_3_) δ = 140.0, 136.1, 128.6, 128.2, 127.5, 126.2, 126.0, 123.7, 79.5 (q, *J*_C-C-F_ = 25 Hz), 30.73, 29.7, 25.9, 1.6. ^19^F-NMR (470 MHz, CDCl_3_) δ = −82.9 (s, 3F), HRMS (ESI) calcd for C_15_H_20_F_3_OSi [M + H]^+^: 301.1230; found 301.1233.

*(4-Benzhydrylidene-2,3-diphenyl-1-trifluoromethylcyclobut-2-enyloxy)trimethylsilane* (**5k**). ^1^H-NMR (400 MHz, CDCl_3_) δ = 7.47–7.39 (m, 4H), 7.28–7.18 (m, 7H), 7.08 (d, *J* = 7.3 Hz, 1H), 7.01 (t, *J* = 7.4 Hz, 2H), 6.85 (dd, *J* = 8.1, 5.2 Hz, 6H), 0.16 (s, 9H). ^13^C-NMR (100 MHz, CDCl_3_) δ = 145.3, 139.5, 133.0, 131.0, 130.4, 130.2, 128.6, 128.1, 127.9, 127.5, 126.9, 126.7, 1.6. ^19^F-NMR (470 MHz, CDCl_3_) δ = −73.2 (s, 3F), HRMS (ESI) calcd for C_33_H_30_F_3_OSi [M + H]^+^: 527.2013; found 527.2017.

All the ^1^H-NMR and ^13^C-NMR spectra of compounds **2a**–**2w** and **5a**–**5k** can be found in Supplementary Materials. CCDC 1487785 contains the supplementary crystallographic data for this paper. These data can be obtained free of charge via http://www.ccdc.cam.ac.uk/conts/retrieving.html (or from the CCDC, 12 Union Road, Cambridge CB2 1EZ, UK; Fax: +44 1223 336033; E-mail: deposit@ccdc.cam.ac.uk).

## 4. Conclusions

In summary, we have developed a simple and very efficient method for the synthesis of trifluoromethylated silyl ethers via the direct nucleophilic trifluoromethylation of ketones, enones, and aldehydes with TMS-CF_3_ in the presence of catalytic amounts of Cs_2_CO_3_. The reaction features an experimentally convenient and atom-economic process for this anionic 1,2-trifluoromethylation. This work also provides a good example of carbonate ion as an active anion that can interact with silicon to promote the chemoselective cleavage of the silicon-carbon bond in TMSCF_3_, but is not effective in the desilylation of trifluoromethylated silyl ethers. 

## Figures and Tables

**Scheme 5:**
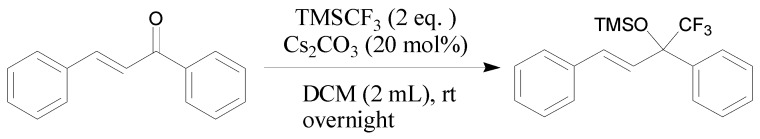
The reaction between TMSCF_3_ and chalcone **1a**.

**Table 1:**
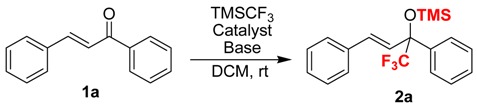
The optimization of reaction conditions for trifluoromethylation of chalcone **1a** with TMSCF_3_.

Entry	Copper	Base	Yield of 2a (%) ^a^
1	Cu_2_(OH)_2_CO_3_	-	NR
2	Cu_2_(OH)_2_CO_3_	K_2_CO_3_	<5
3	Cu_2_(OH)_2_CO_3_	KOAc	NR
4	Cu_2_(OH)_2_CO_3_	KH_2_PO_4_	NR
5	Cu_2_(OH)_2_CO_3_	KHF_2_	57
6	Cu_2_(OH)_2_CO_3_	KF	<5
7	Cu_2_(OH)_2_CO_3_	KCl	NR
8	Cu_2_(OH)_2_CO_3_	KBr	NR
9	Cu_2_(OH)_2_CO_3_	KOH	52
10	Cu_2_(OH)_2_CO_3_	*t*-BuOK	55
11	Cu_2_(OH)_2_CO_3_	Cs_2_CO_3_	60
12	Cu_2_(OH)_2_CO_3_	LiOH	<5
13	-	Cs_2_CO_3_	94
14	-	CaF_2_	37
15	-	KF	NR
16	-	BaF_2_	NR
17	-	K_2_CO_3_	72 ^b^
18	-	KOAc	NR
19	-	KOH	83
20	-	*t-*BuOK	71

^a^ Isolated yield, ^b^ The reaction time was 36 h; NR = no reaction.

**Table 2:**
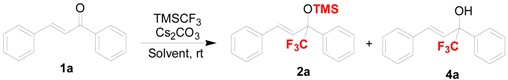
The solvent effect on the Cs_2_CO_3_-catalyzed trifluoromethylation of chalcone **1a** with TMSCF_3_.

Entry	Solvent	Yield of 2a (%) ^a^	Yield of 4a (%) ^a^
1	DCM	94	0
2	DCE	NR	NR
3	THF	36	51
4	DMSO	NR	NR
5	MeCN	NR	NR
6	toluene	15	0
7	dioxane	NR	NR
8	Et_2_O	60	0
9	CHCl_3_	NR	NR
10	DMF	0	83
11	DMA	0	81
12	TMSOTMS	68	0
13	*n*-Hexane	61	0

^a^ Isolated yield; NR = no reaction.

## Supplementary Materials

Supplementary Materials are available online.
