# Supplementary material for: Cs2CO3-Initiated Trifluoro-Methylation of Chalcones and Ketones for Practical Synthesis of Trifluoromethylated Tertiary Silyl Ethers
Source: Molecules. 2017 May 18;22(5):769. doi: 10.3390/molecules22050769 (PMC6154635; doi:10.3390/molecules22050769)

Supporting Information

Cs_2CO_3–Initiated Trifluoromethylation of Chalcones and Ketones for Practical Synthesis of Trifluoromethylated Tertiary Silyl Ethers

Cheng Dong, Xing-Feng Bai, Ji-Yuan Lv, Yu-Ming Cui, Jian Cao, Zhan-Jiang Zheng*, Li-Wen Xu*

Key Laboratory of Organosilicon Chemistry and Material Technology of Ministry of Education, Hangzhou Normal University, Hangzhou 311121, P. R. China.Fax: +86-571-28865135; Tel: +86-571-28865135; E-mail: zzjiang78@hznu.edu.cn, liwenxu@hznu.edu.cn.

Table of Contents

^{1}H NMR and ^{13}C NMR spectra for compound 2a-2w……………………………S2

^{1}H NMR and ^{13}C NMR spectra for compound 5a-5k……………………………S25

Figure S1 ^{1}H NMR spectra for compound 2a

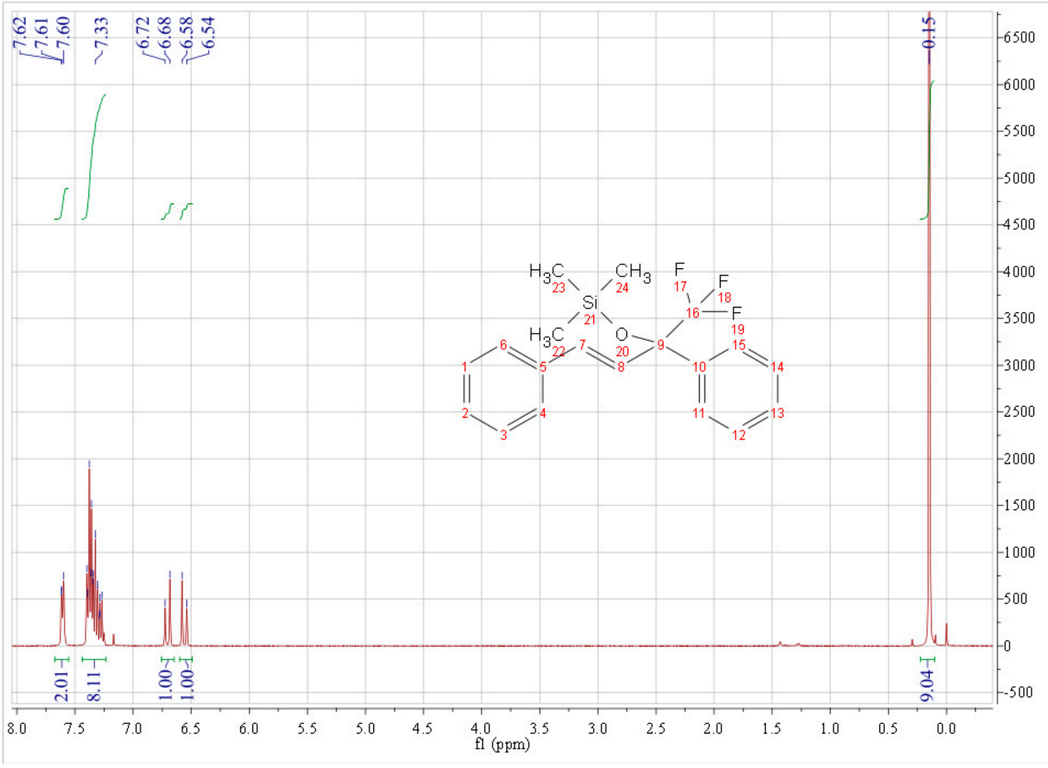

Figure S2 ^{13}C NMR spectra for compound 2a

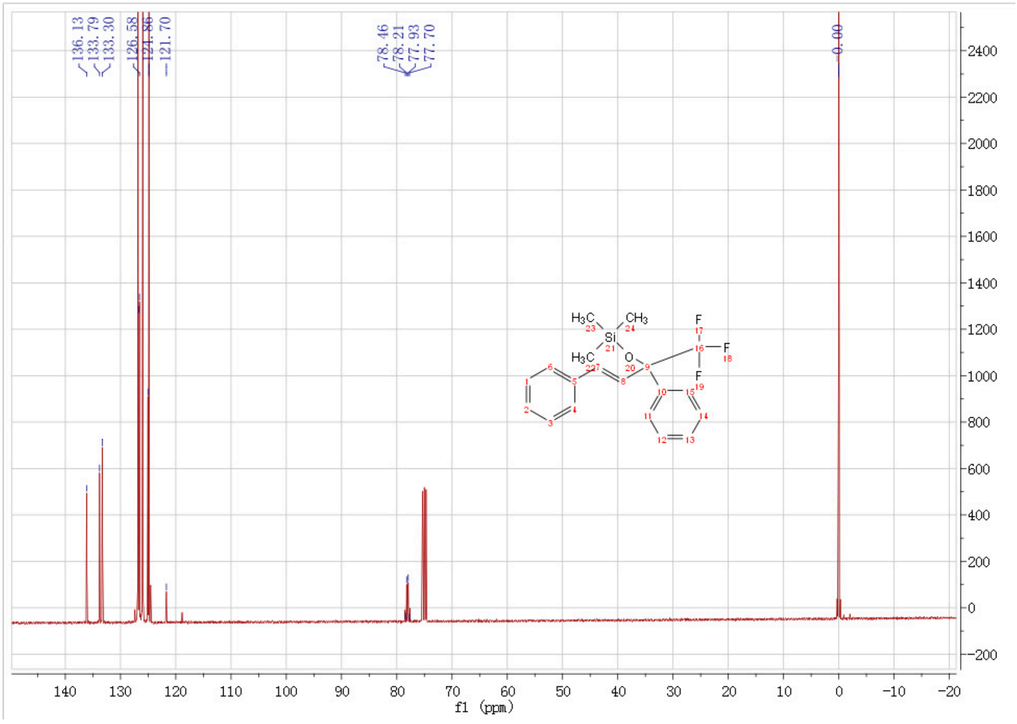

Figure S3 ^{1}H NMR spectra for compound 2b

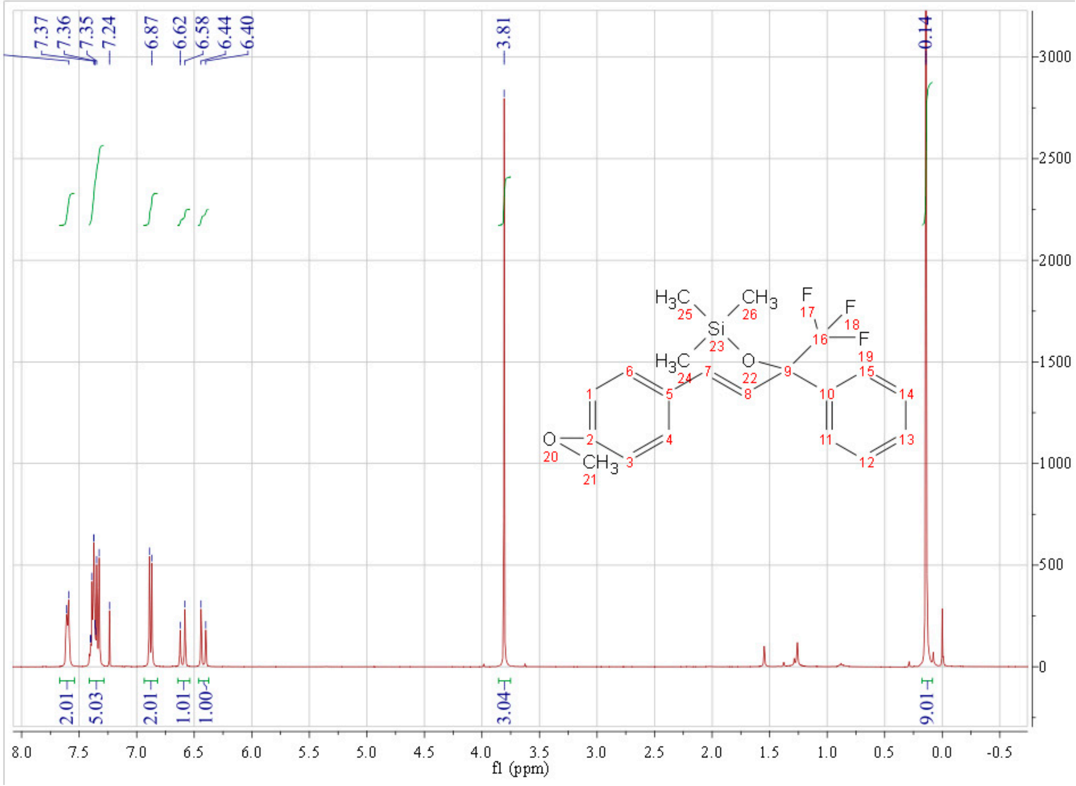

Figure S4 ^{13}C NMR spectra for compound 2b

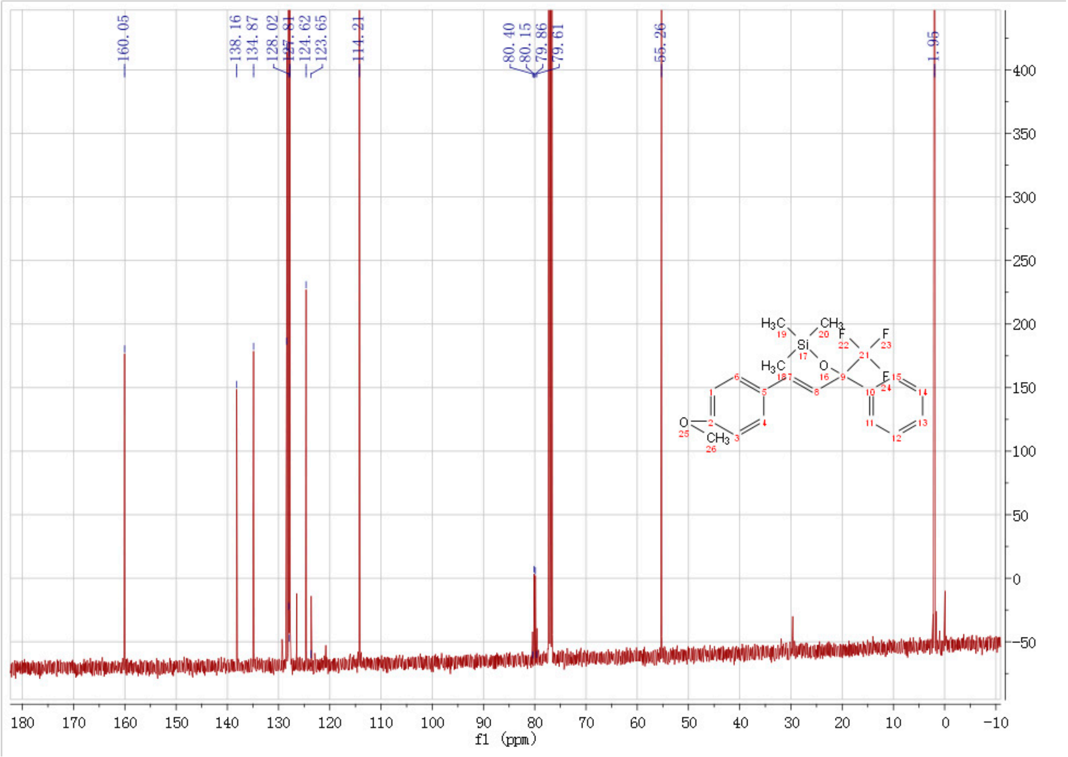

Figure S5 ^{1}H NMR spectra for compound 2c

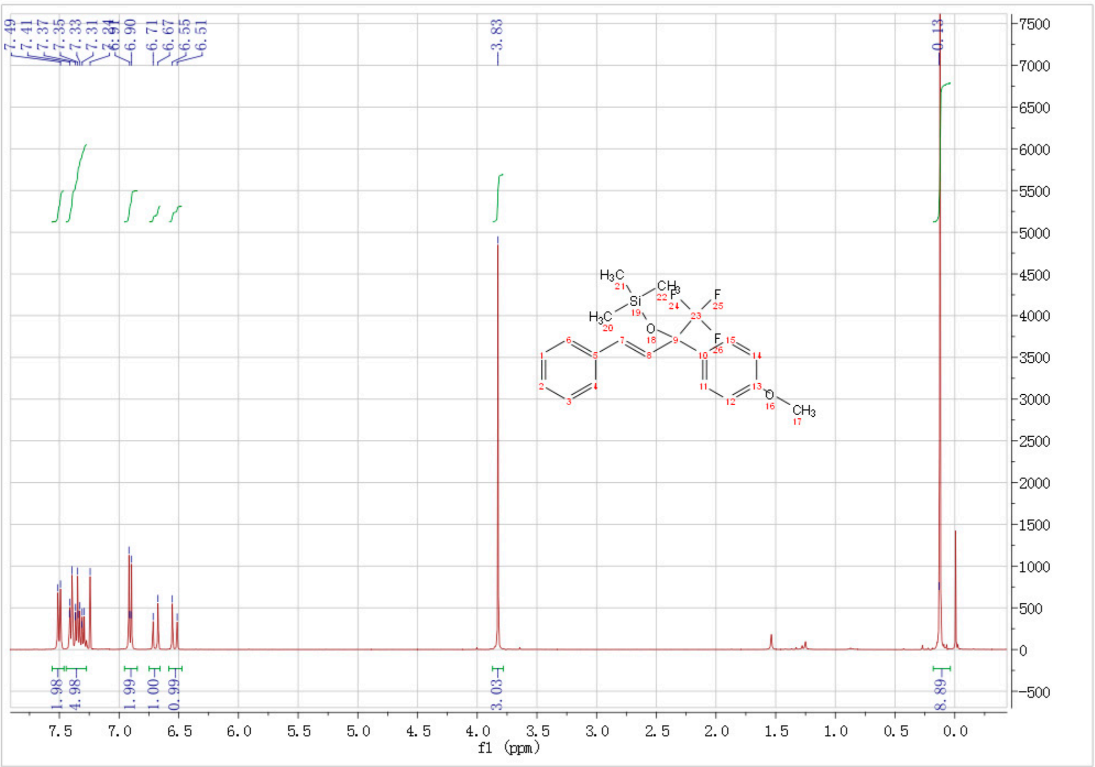

Figure S6 ^{13}C NMR spectra for compound 2c

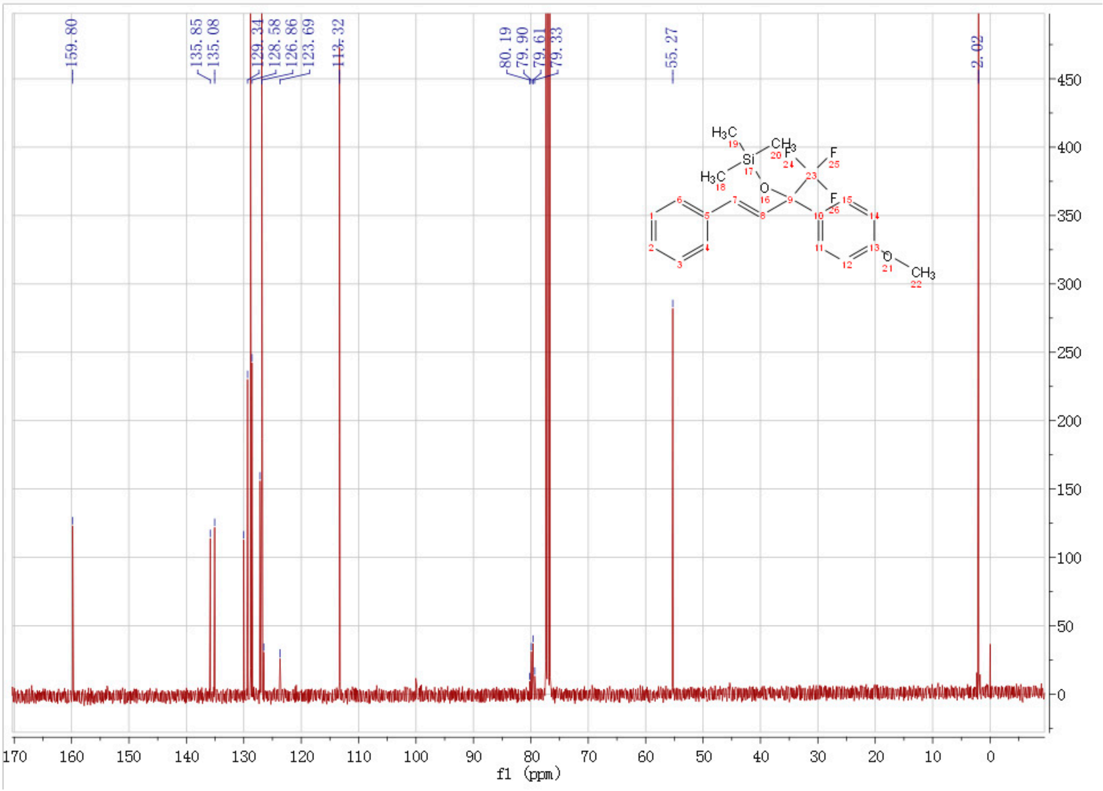

Figure S7 ^{1}H NMR spectra for compound 2d

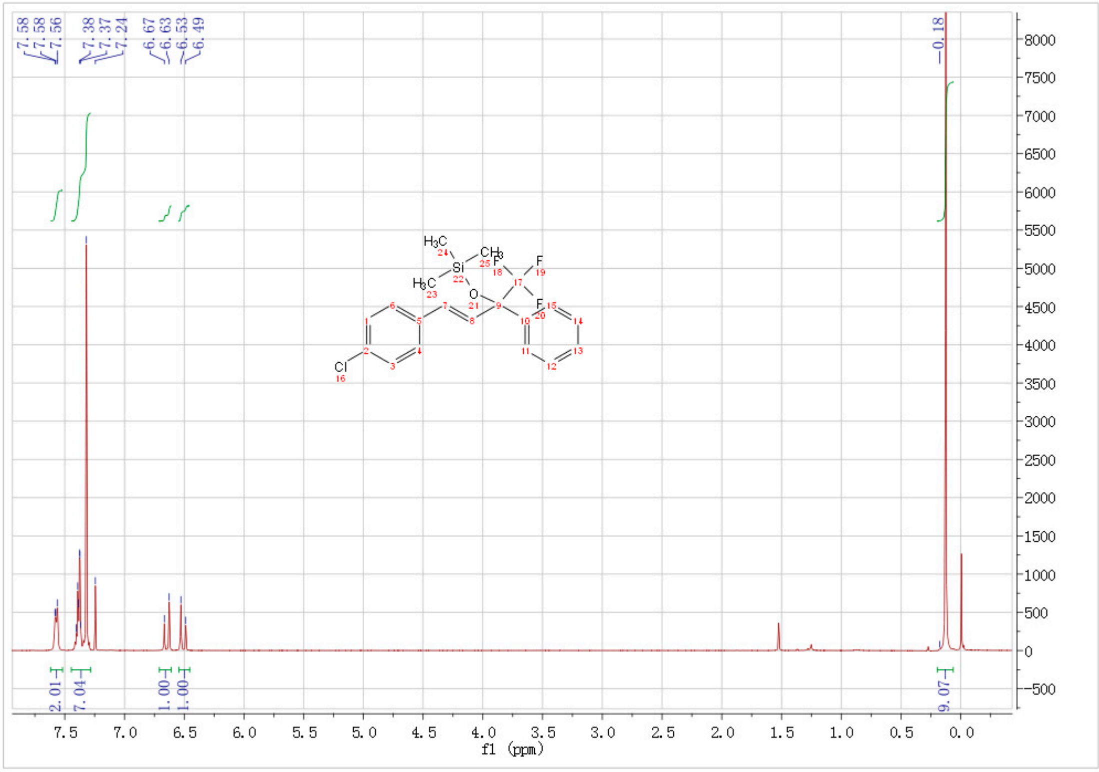

Figure S8 ^{13}C NMR spectra for compound 2d

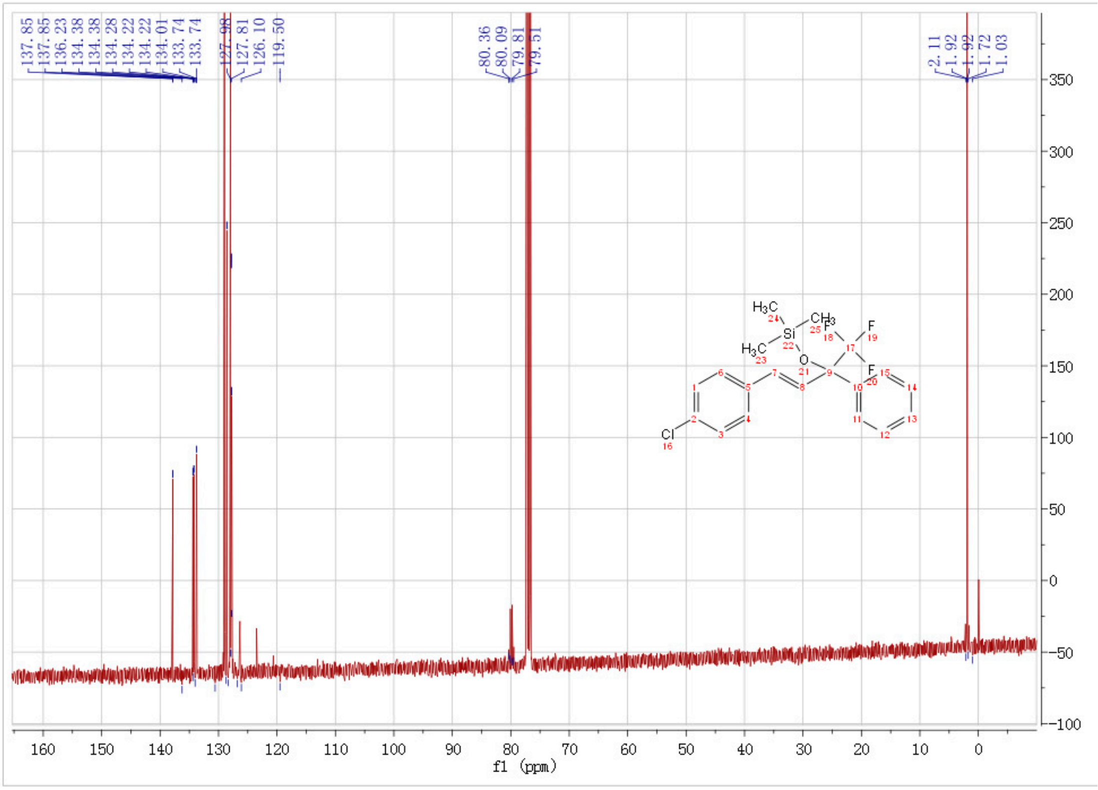

Figure S9 ^{1}H NMR spectra for compound 2e

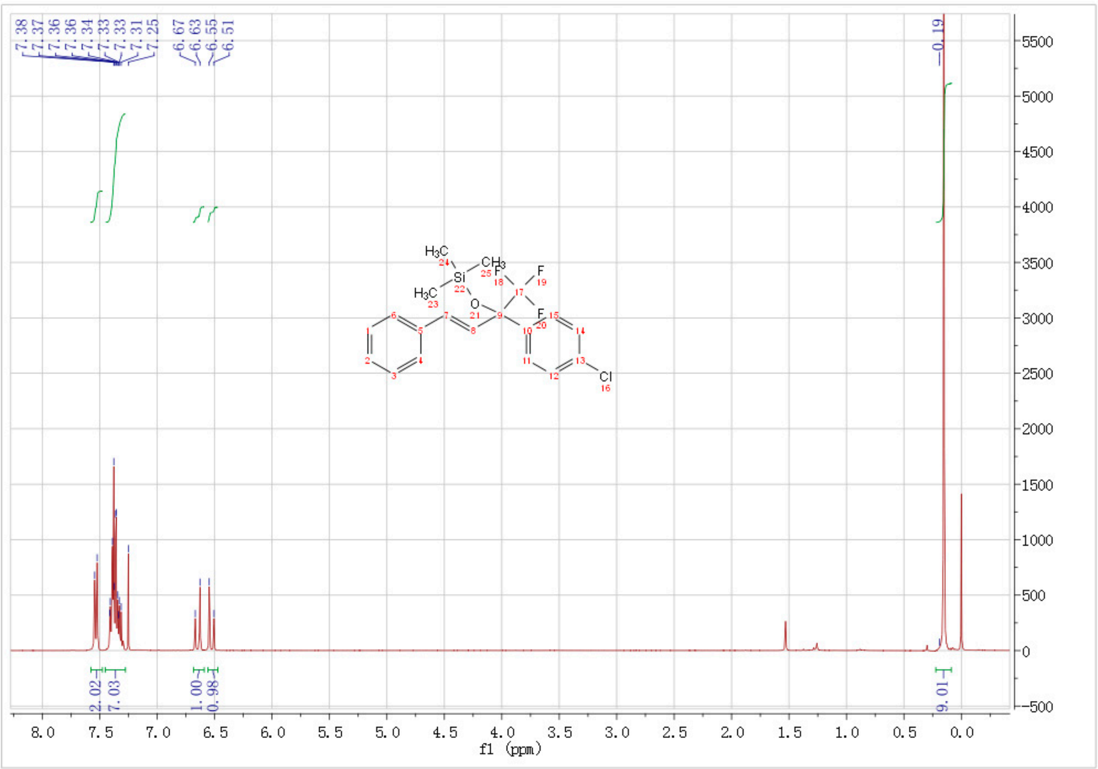

Figure S10 ^{13}C NMR spectra for compound 2e

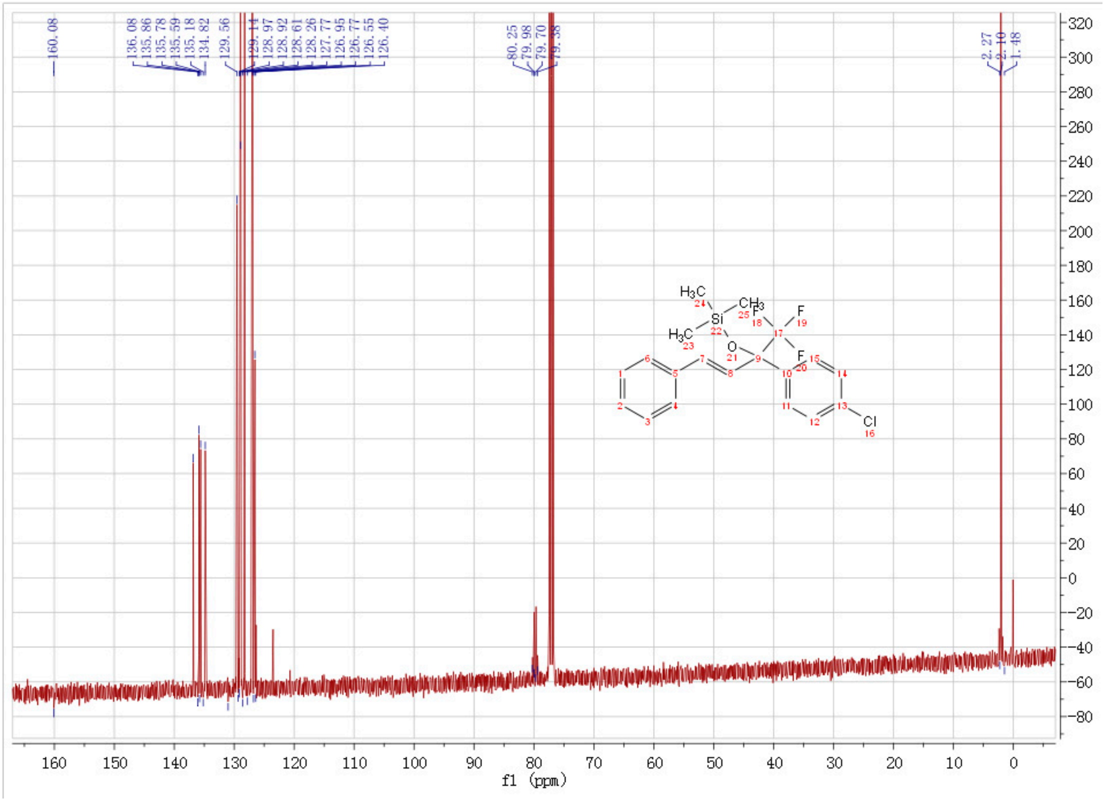

Figure S11 ^{1}H NMR spectra for compound 2f

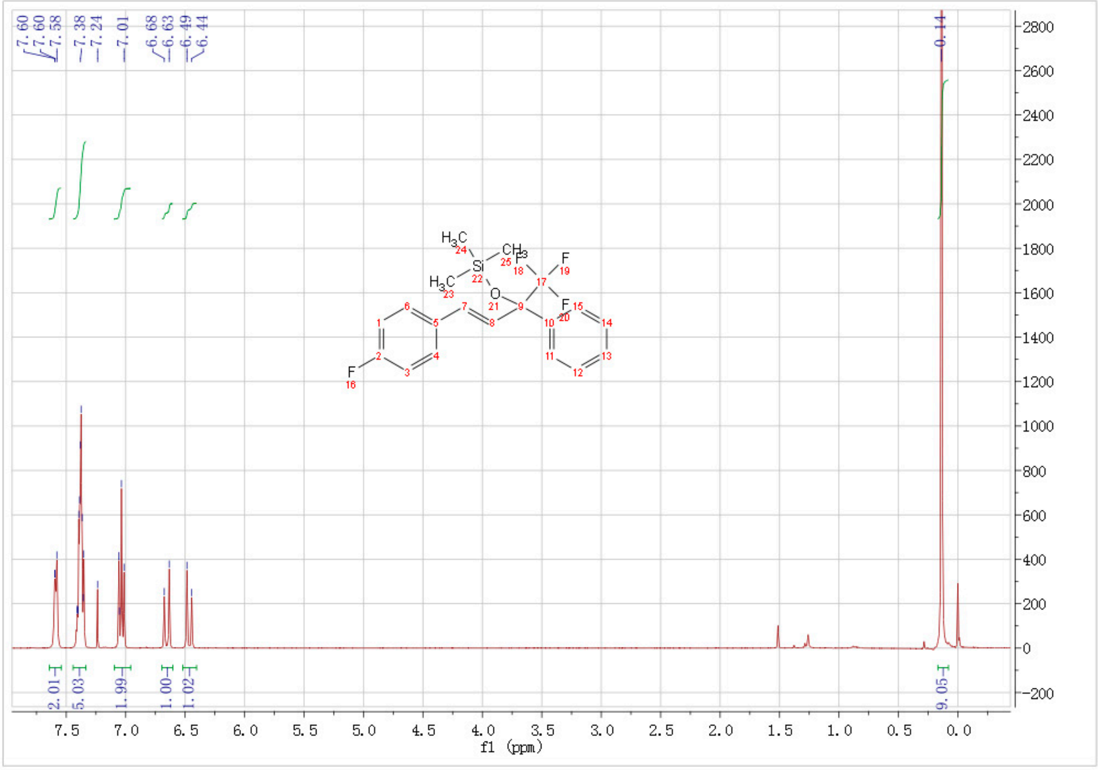

Figure S12 ^{13}C NMR spectra for compound 2f

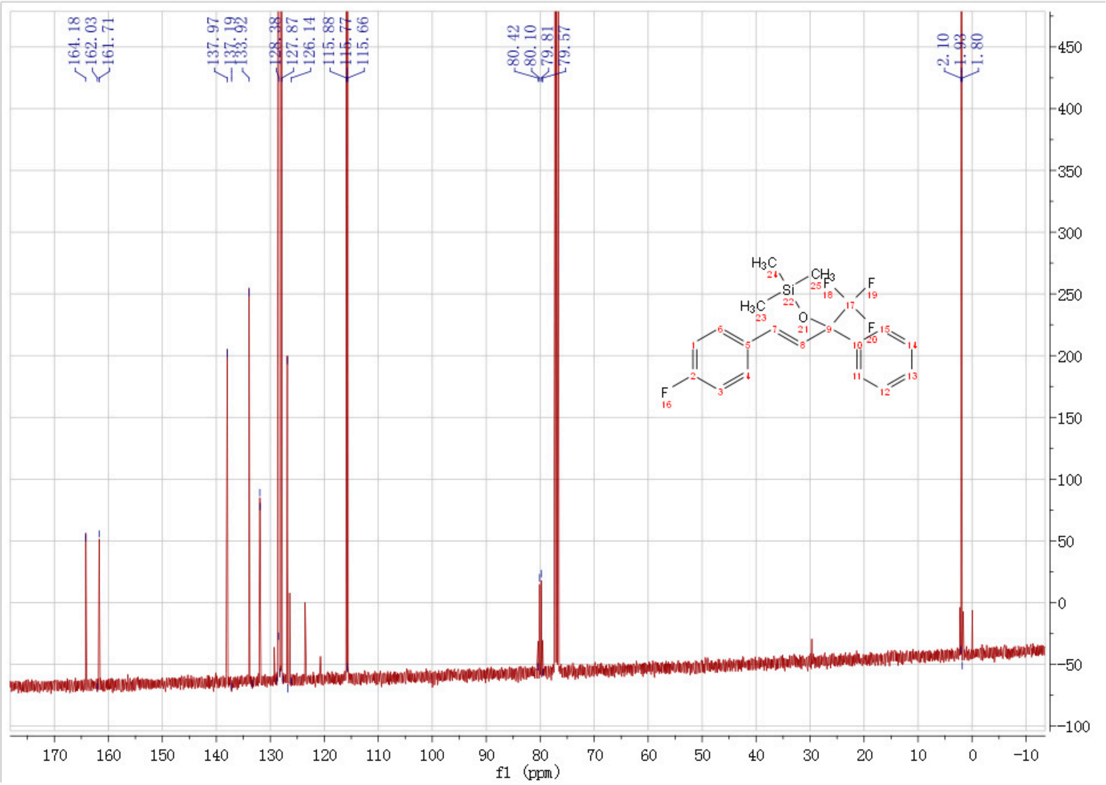

Figure S13 ^{13}C NMR spectra for compound 2g

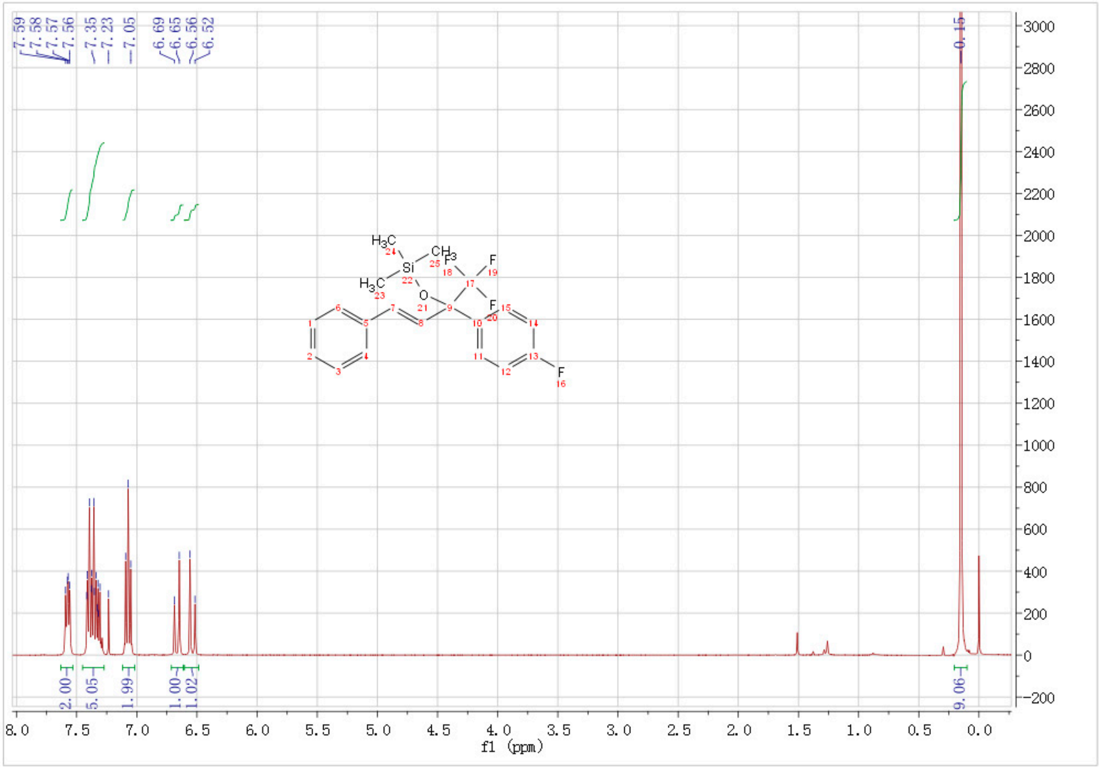

Figure S14 ^{13}C NMR spectra for compound 2g

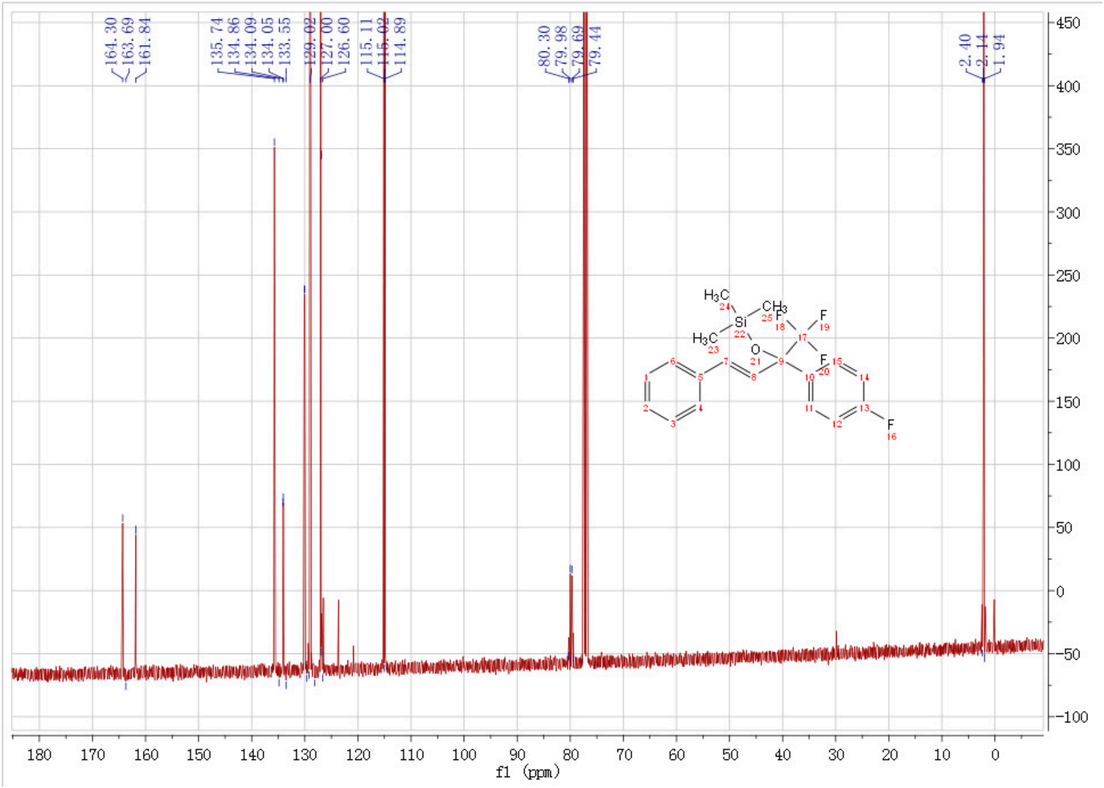

Figure S15 ^{1}H NMR spectra for compound 2h

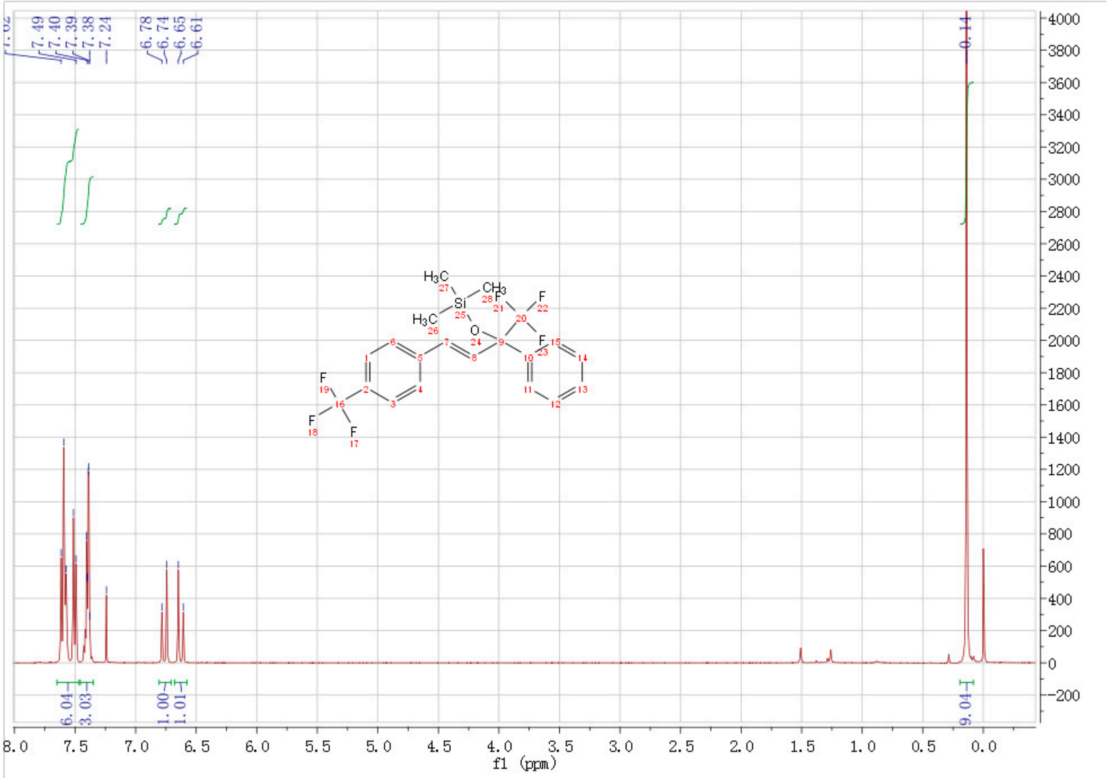

Figure S16 ^{13}C NMR spectra for compound 2h

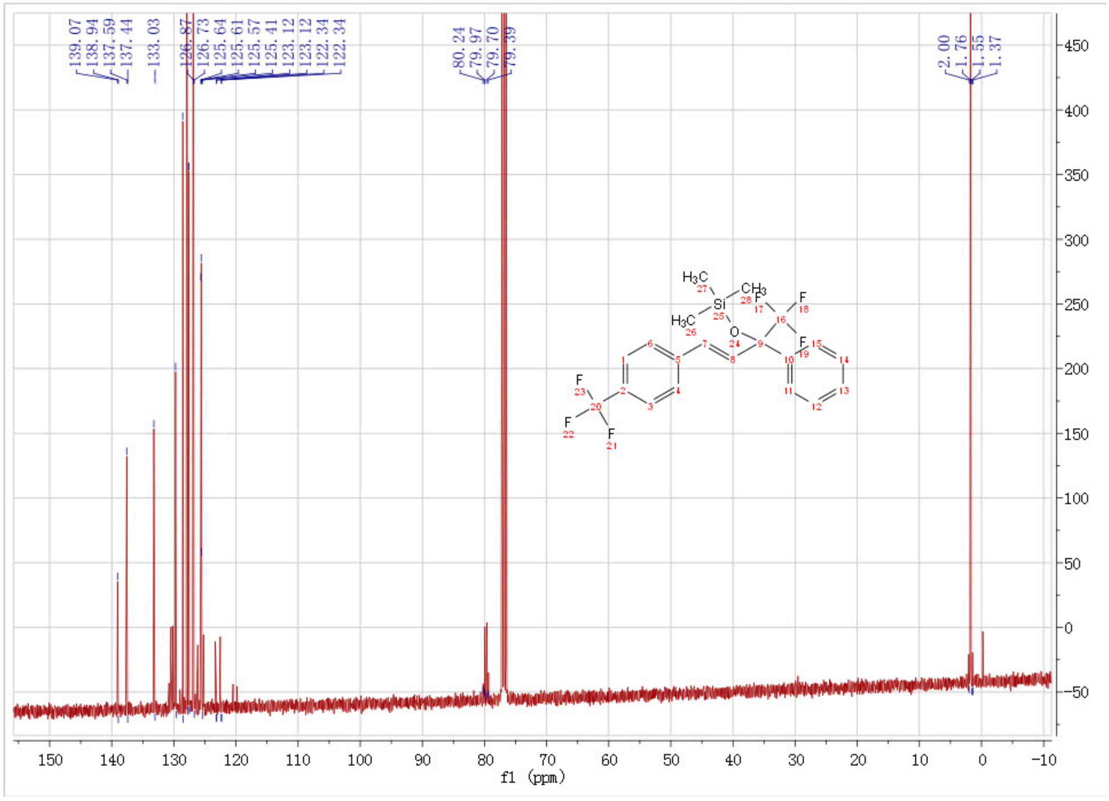

Figure S17 ^{13}C NMR spectra for compound 2i

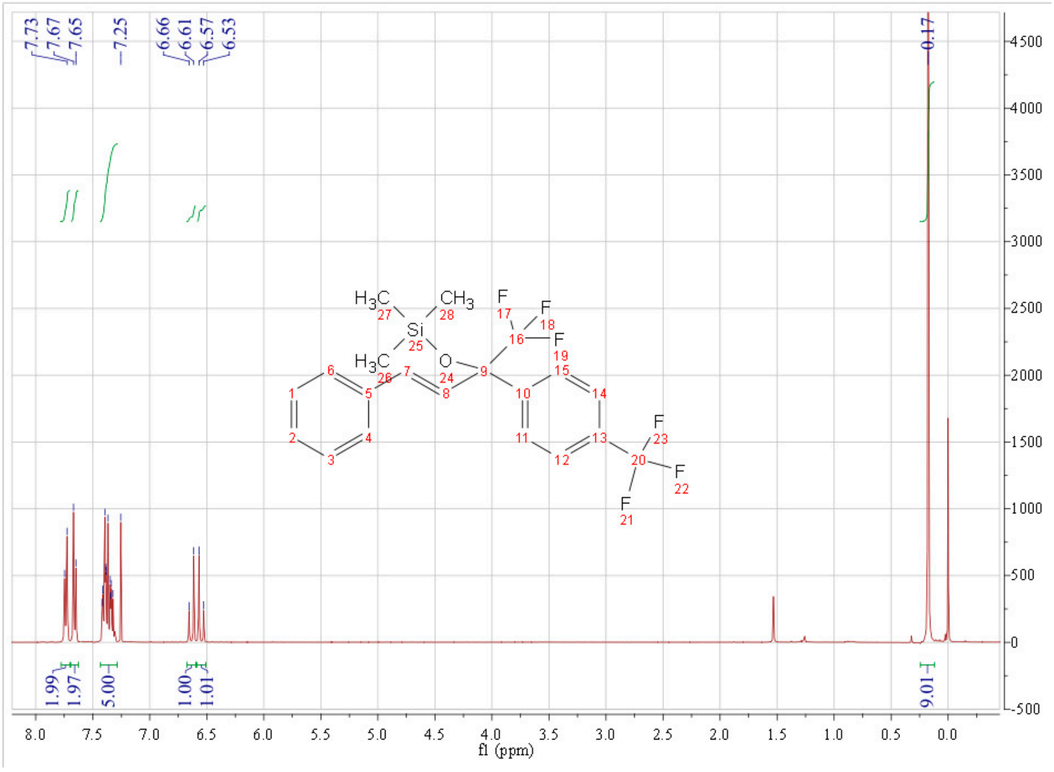

Figure S18 ^{13}C NMR spectra for compound 2i

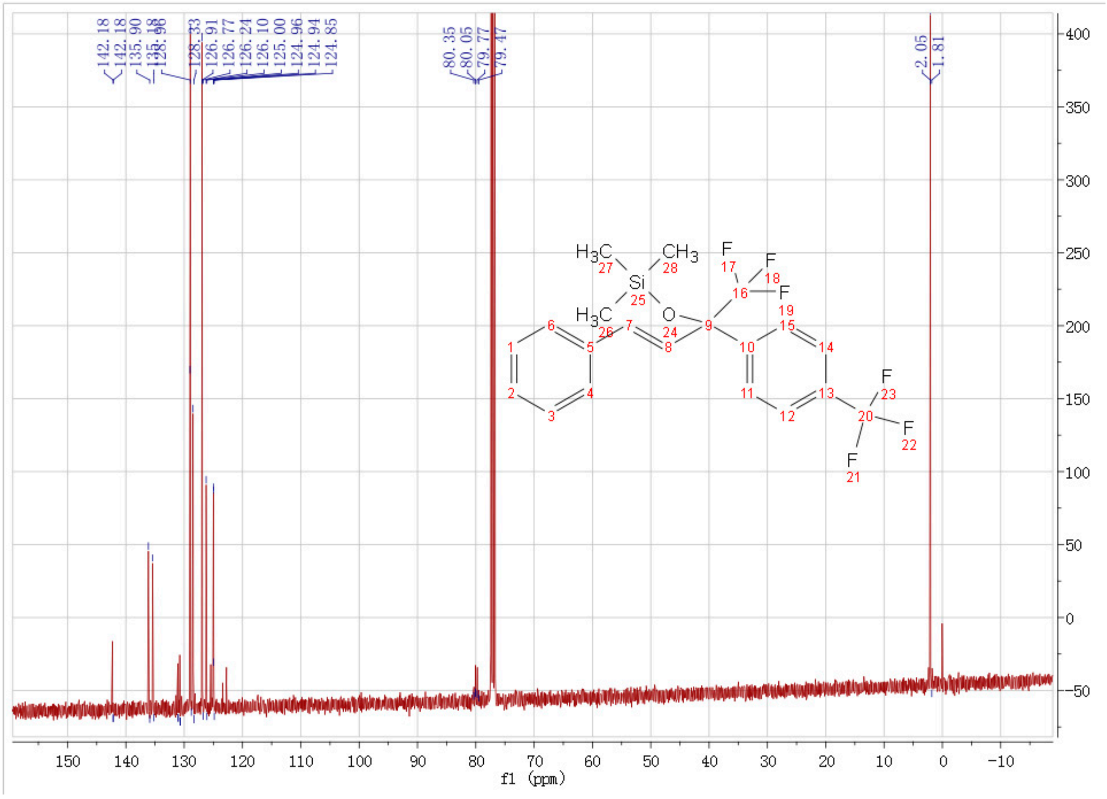

Figure S19 ^{1}H NMR spectra for compound 2j

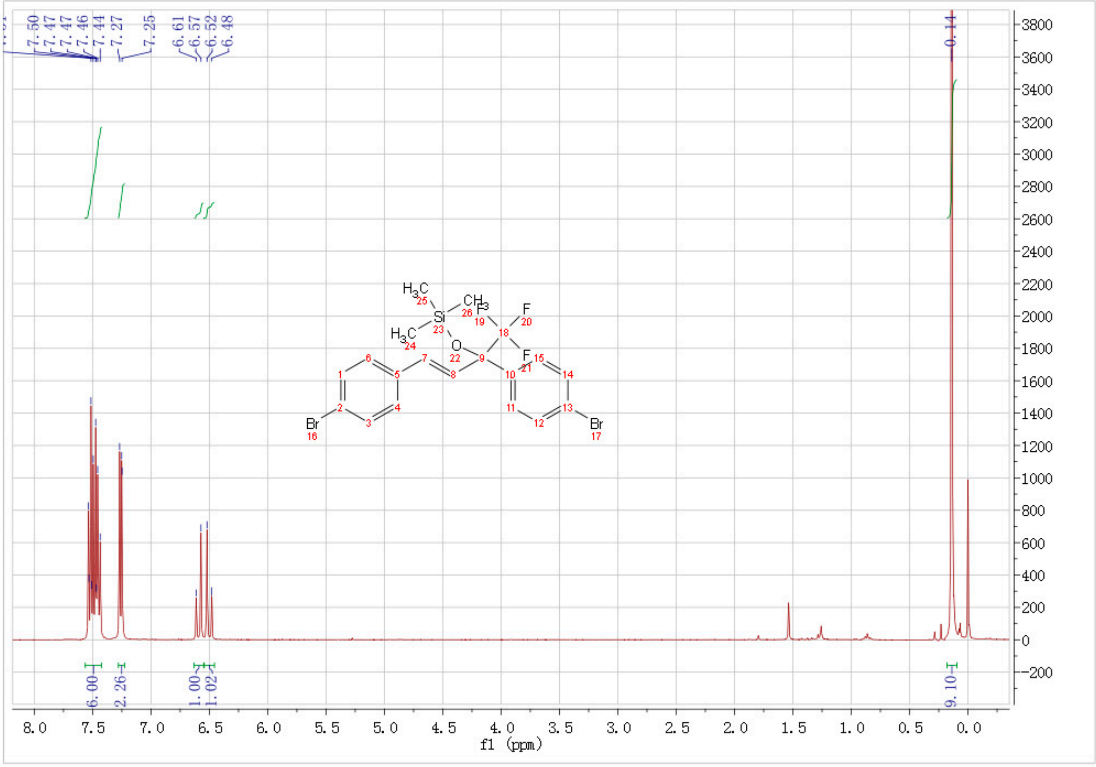

Figure S20 ^{13}C NMR spectra for compound 2j

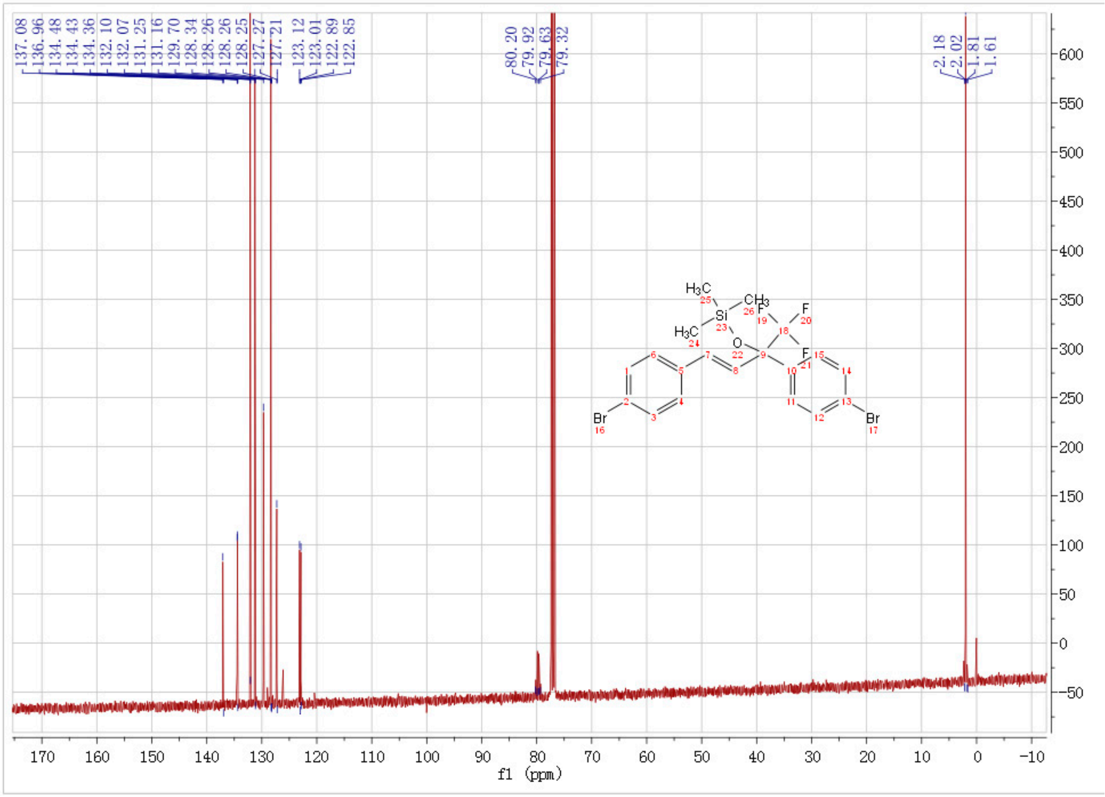

Figure S21 ^{1}H NMR spectra for compound 2k

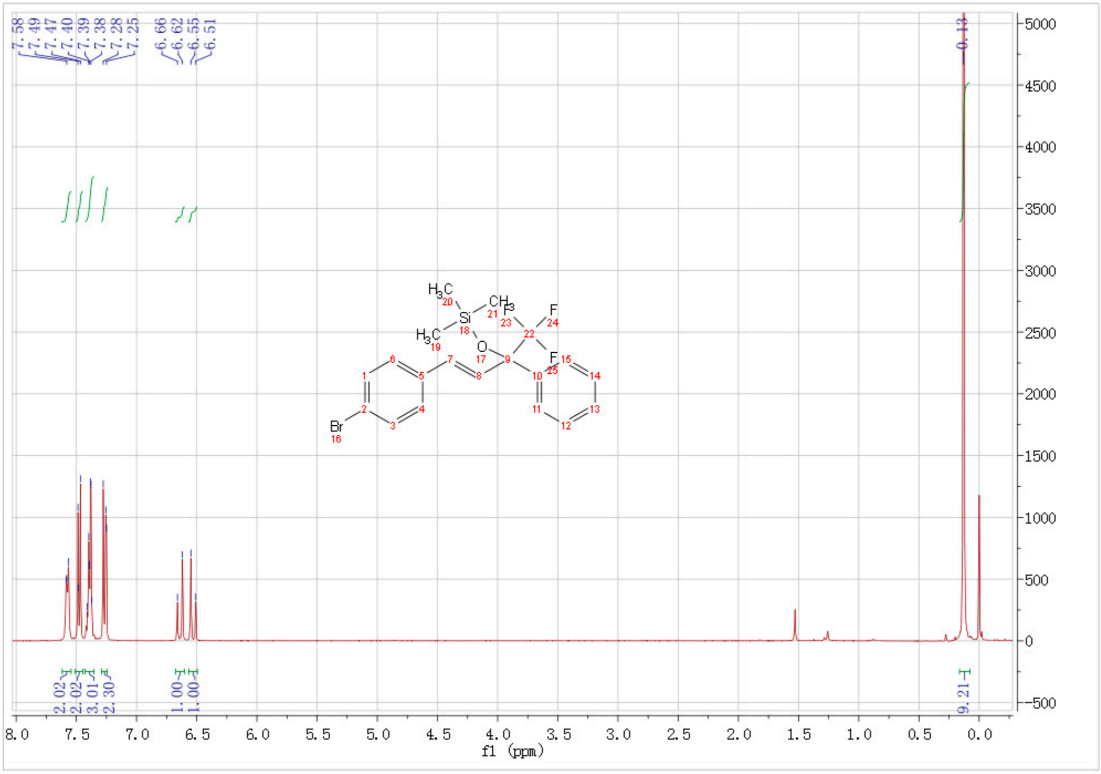

Figure S22 ^{13}C NMR spectra for compound 2k

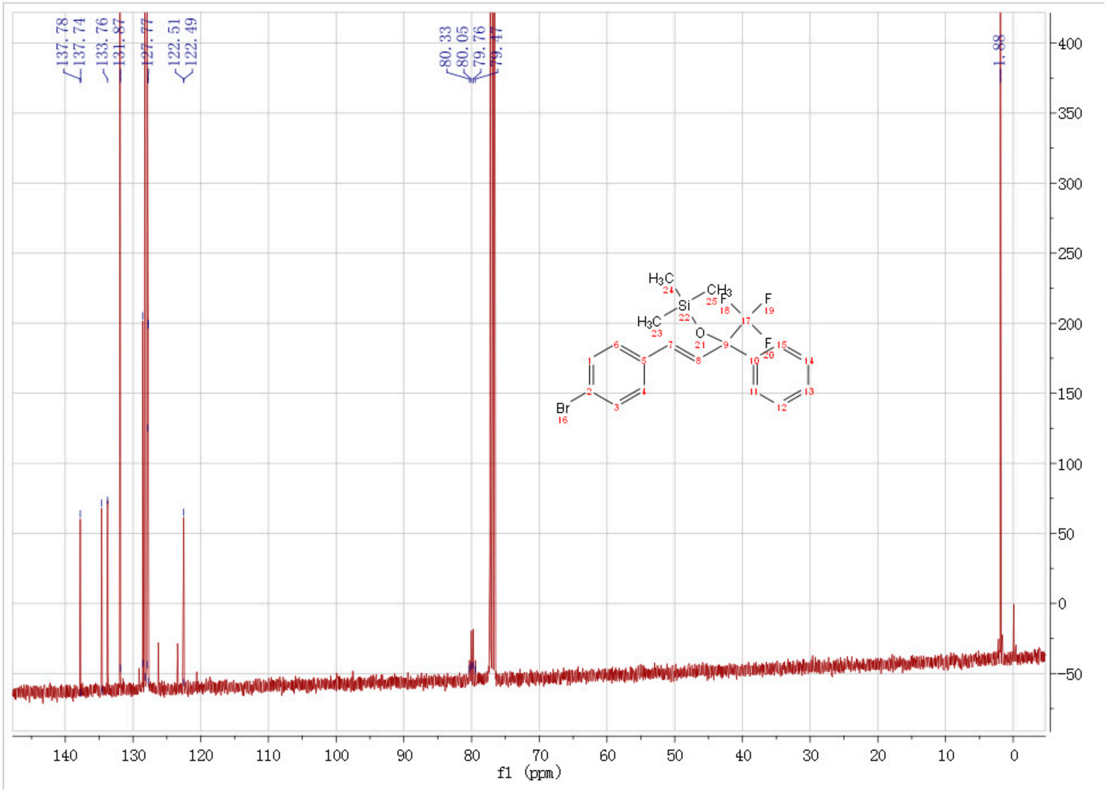

Figure S23 ^{1}H NMR spectra for compound 2l

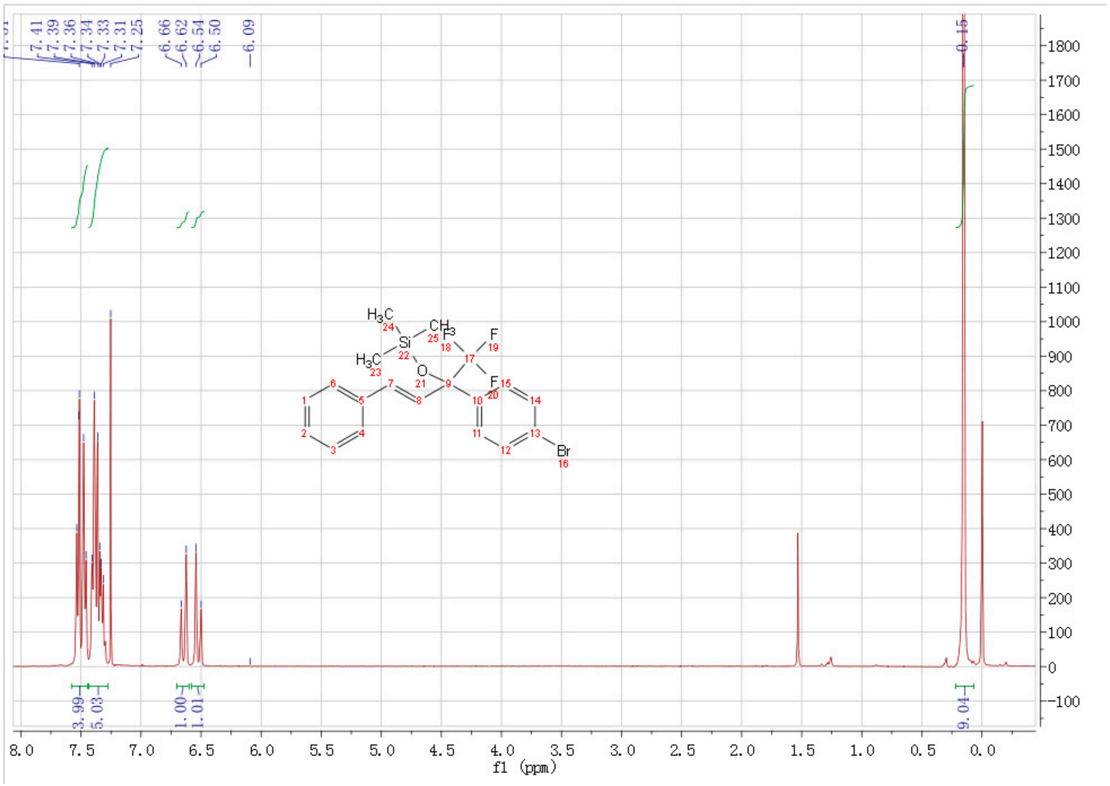

Figure S24 ^{13}C NMR spectra for compound 2l

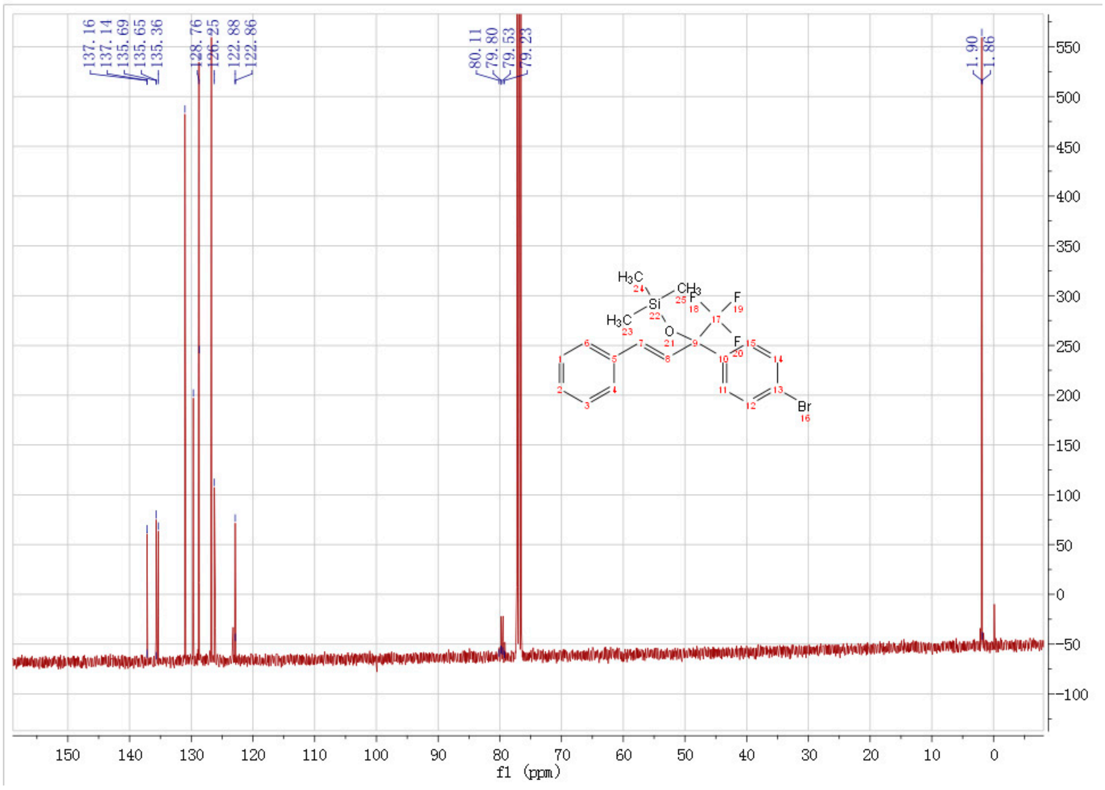

Figure S25 ^{1}H NMR spectra for compound 2m

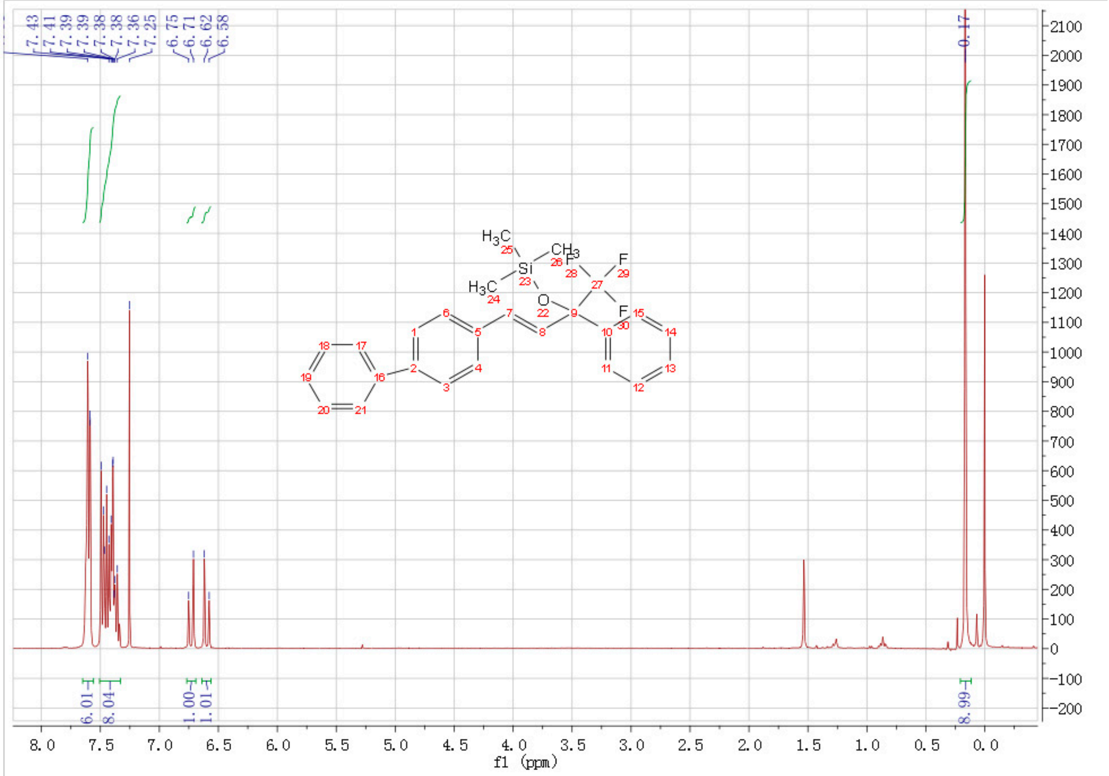

Figure S26 ^{13}C NMR spectra for compound 2m

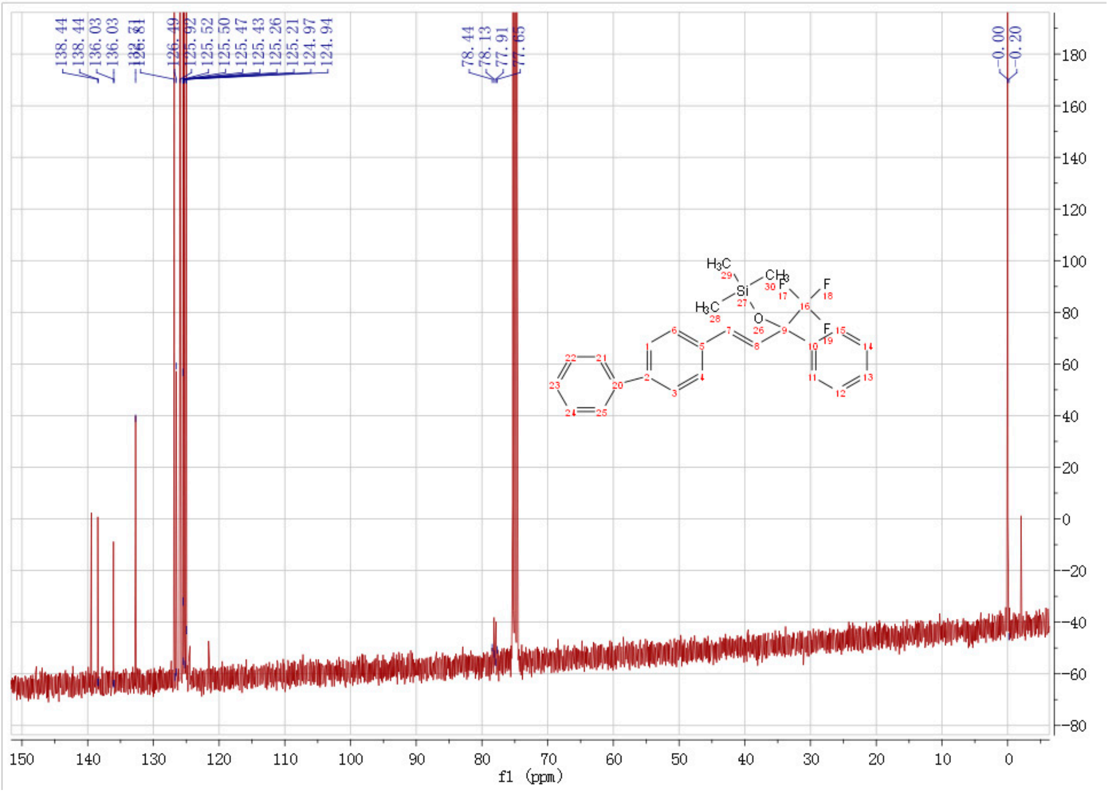

Figure S27 ^{1}H NMR spectra for compound 2n

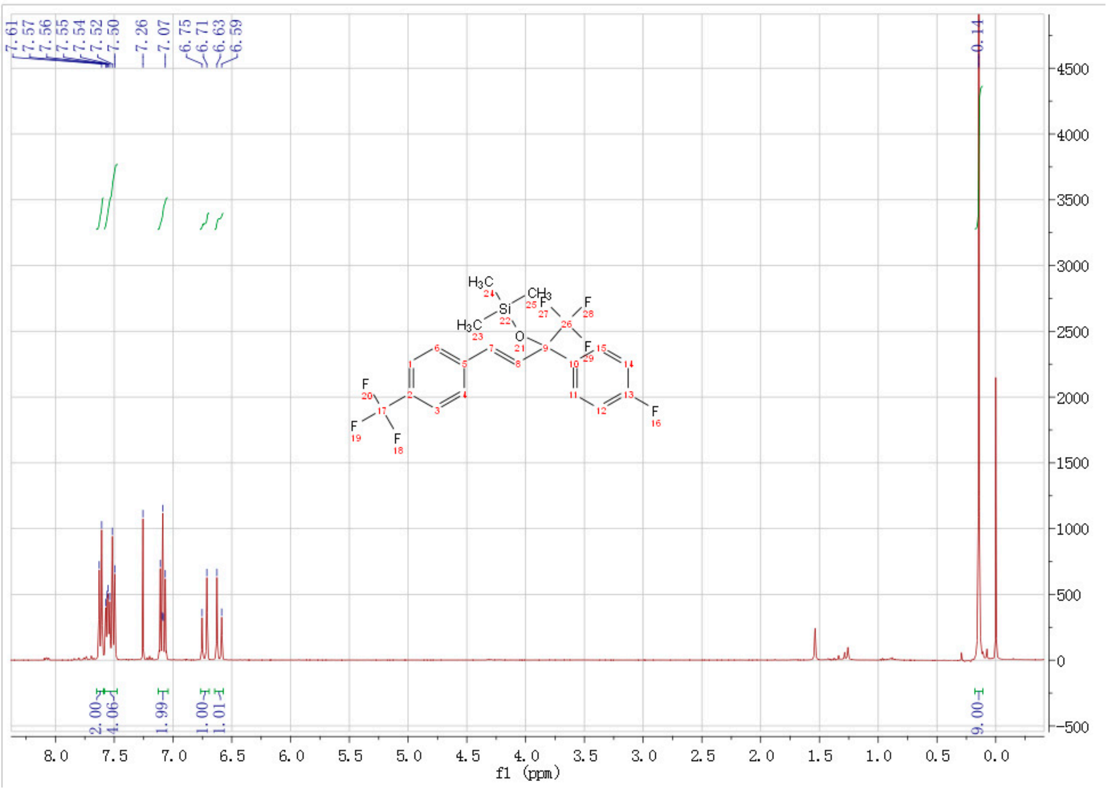

Figure S28 ^{13}C NMR spectra for compound 2n

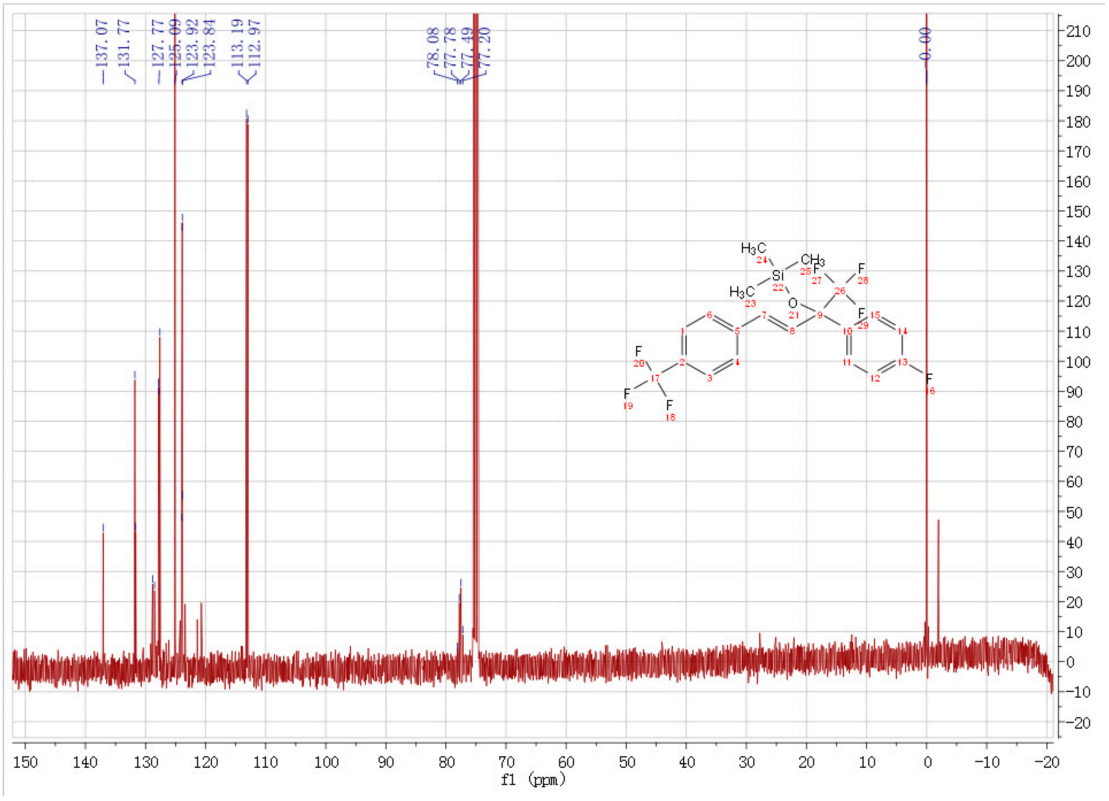

Figure S29 ^{1}H NMR spectra for compound 2o

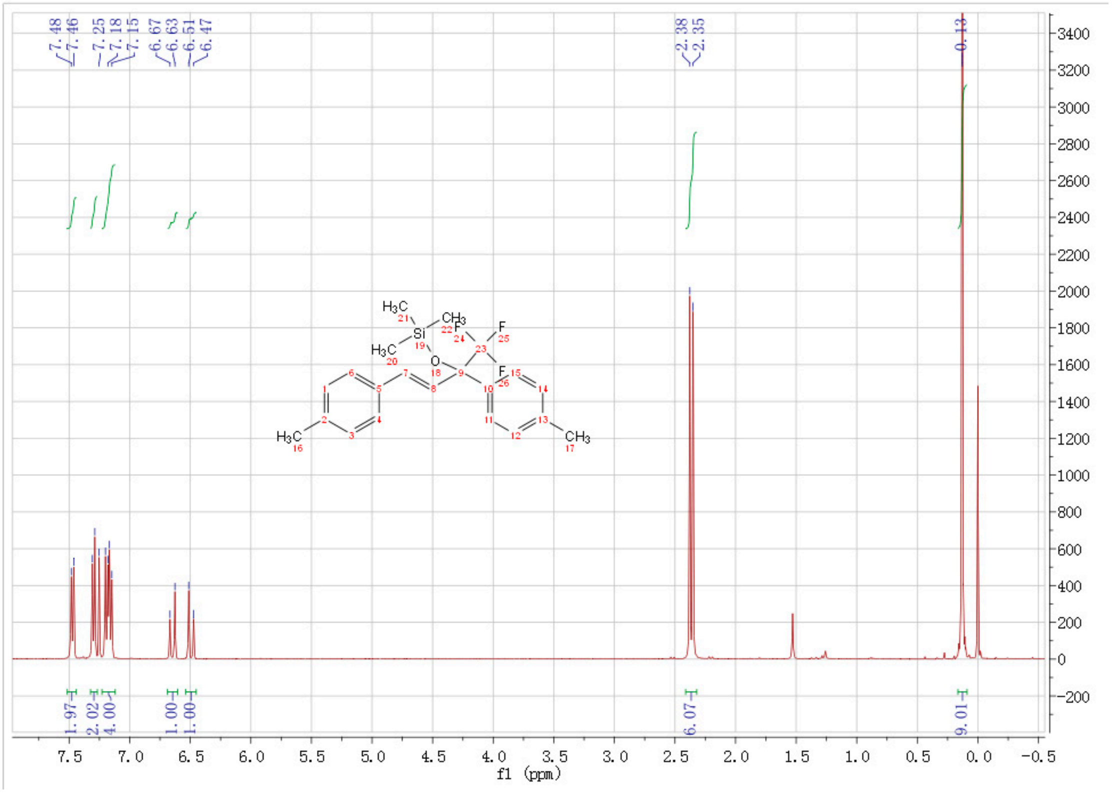

Figure S30 ^{13}C NMR spectra for compound 2o

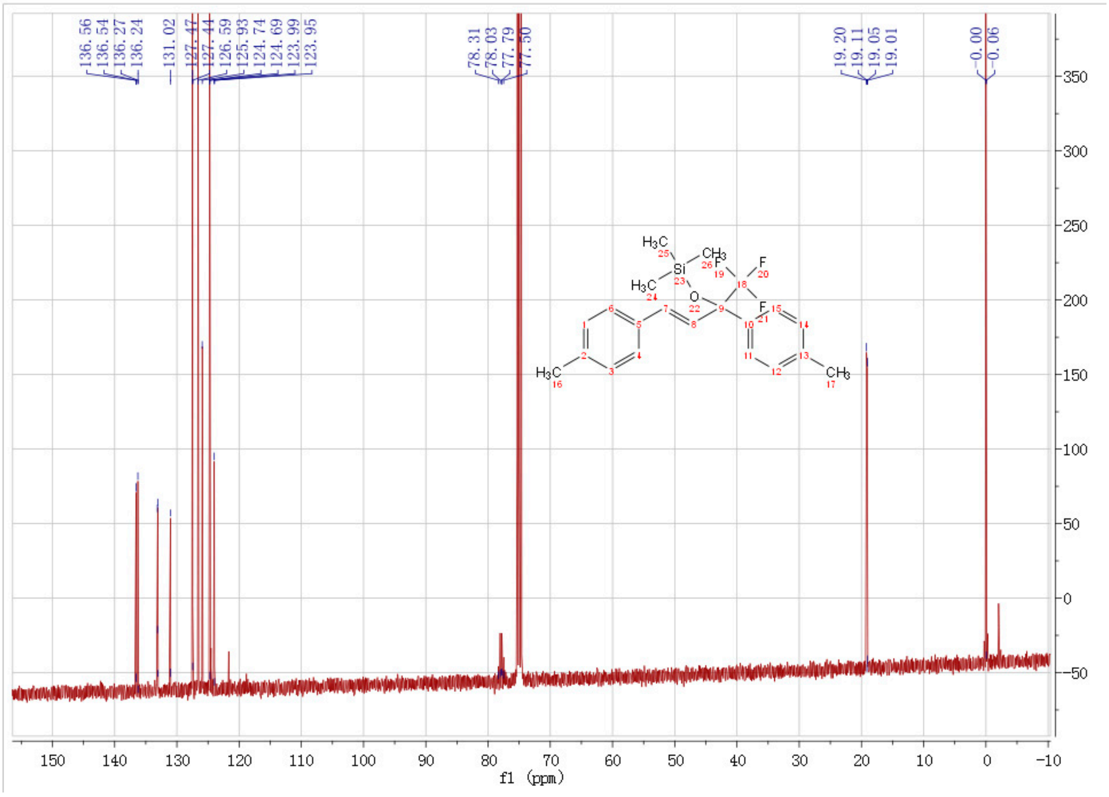

Figure S31 ^{1}H NMR spectra for compound 2p

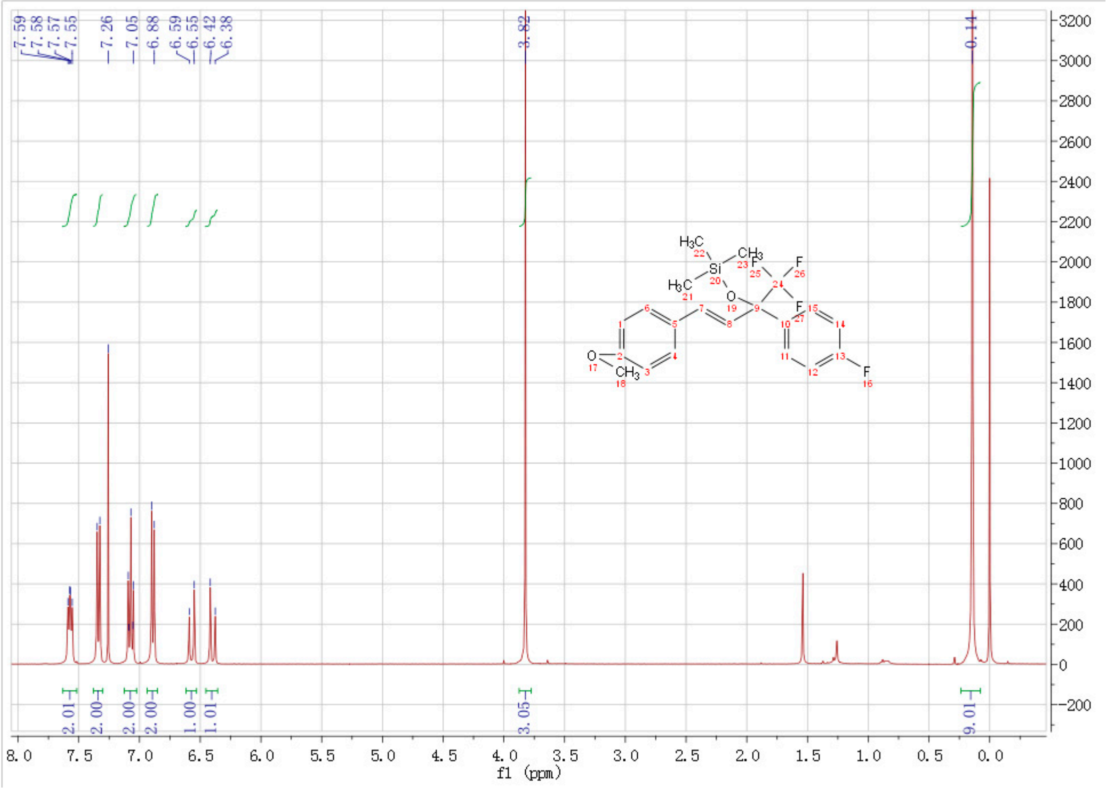

Figure S32 ^{13}C NMR spectra for compound 2p

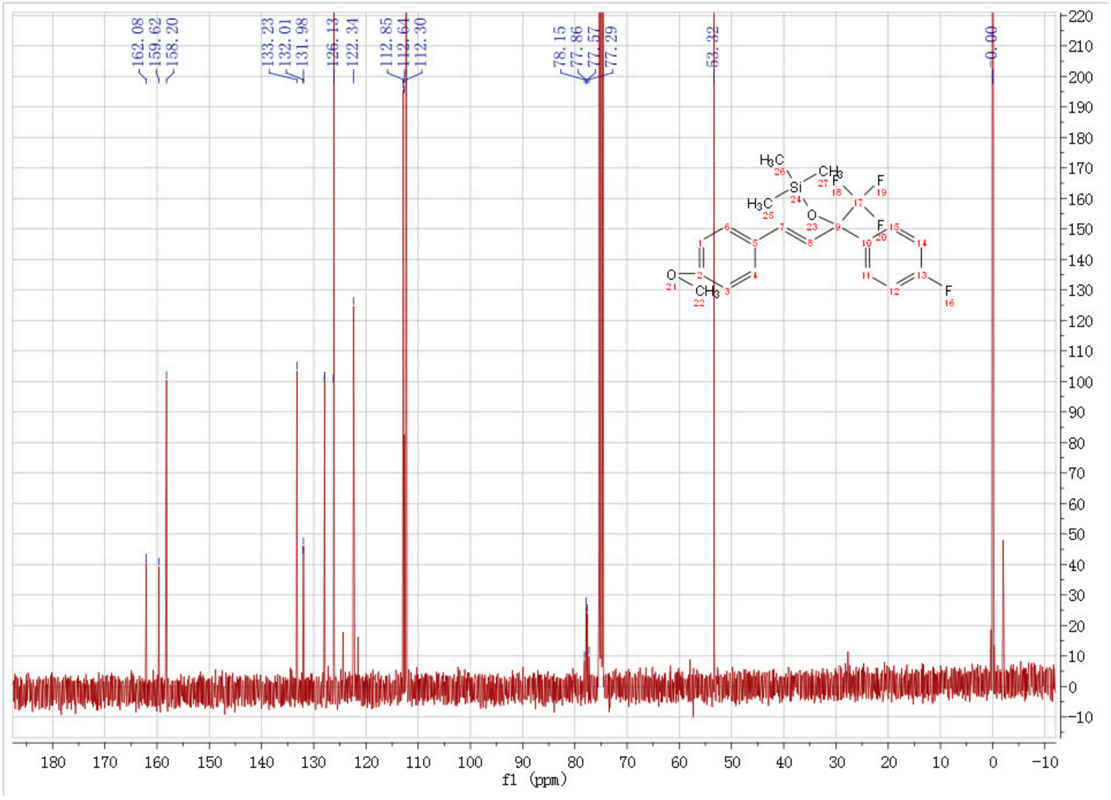

Figure S33 ^{1}H NMR spectra for compound 2q

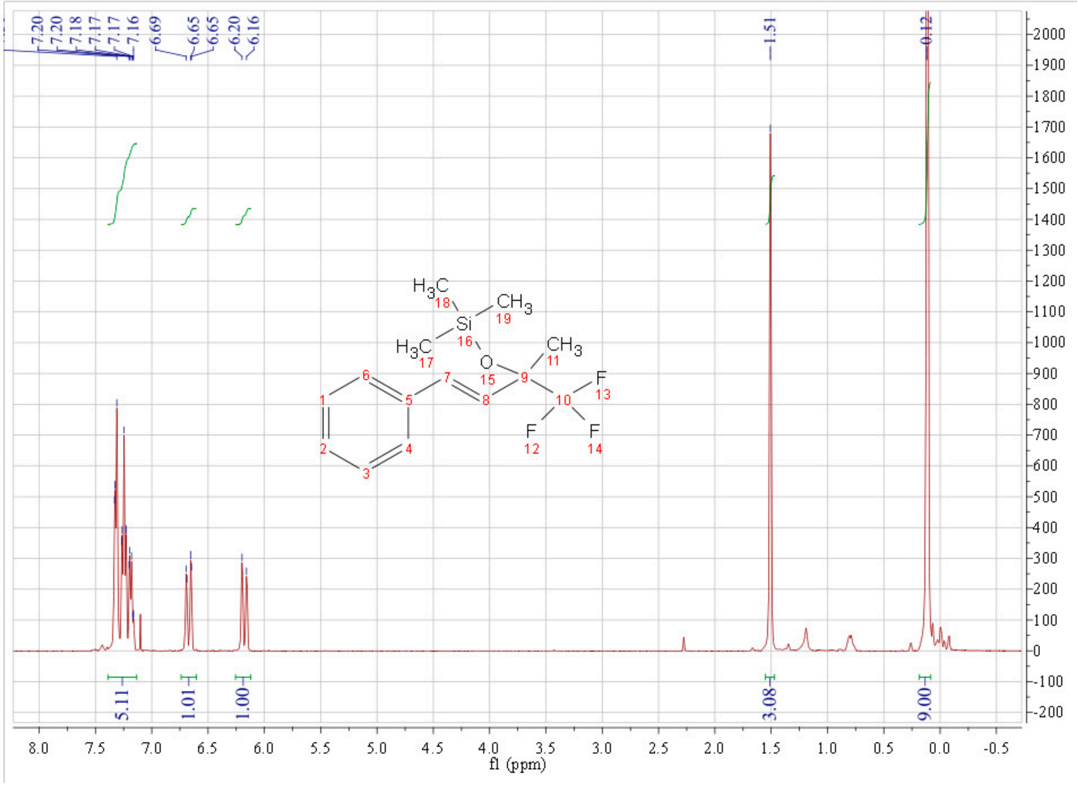

Figure S34 ^{13}C NMR spectra for compound 2q

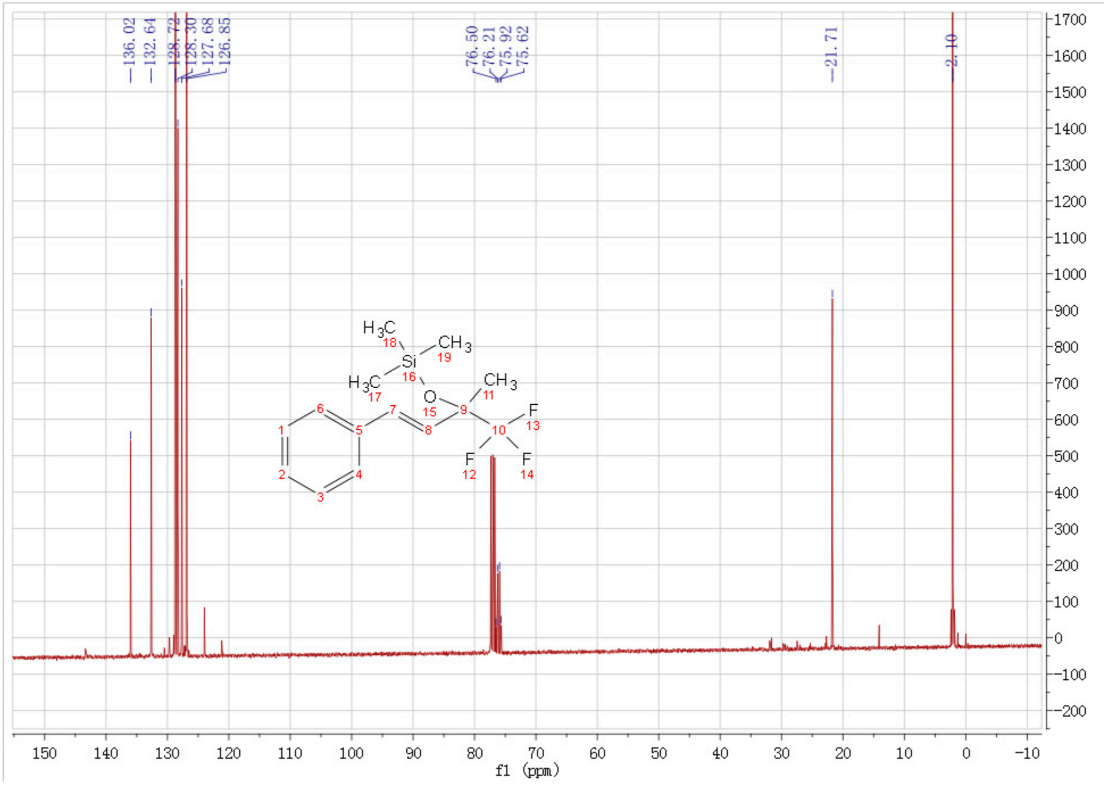

Figure S35 ^{1}H NMR spectra for compound 2r

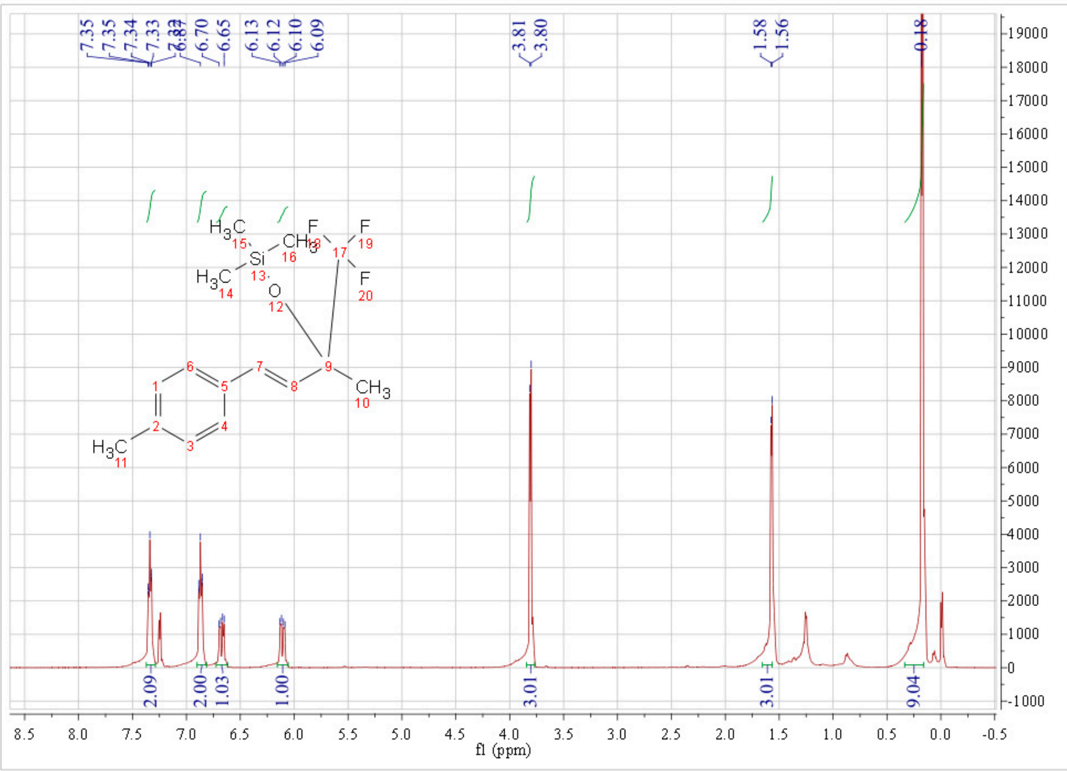

Figure S36 ^{13}C NMR spectra for compound 2r

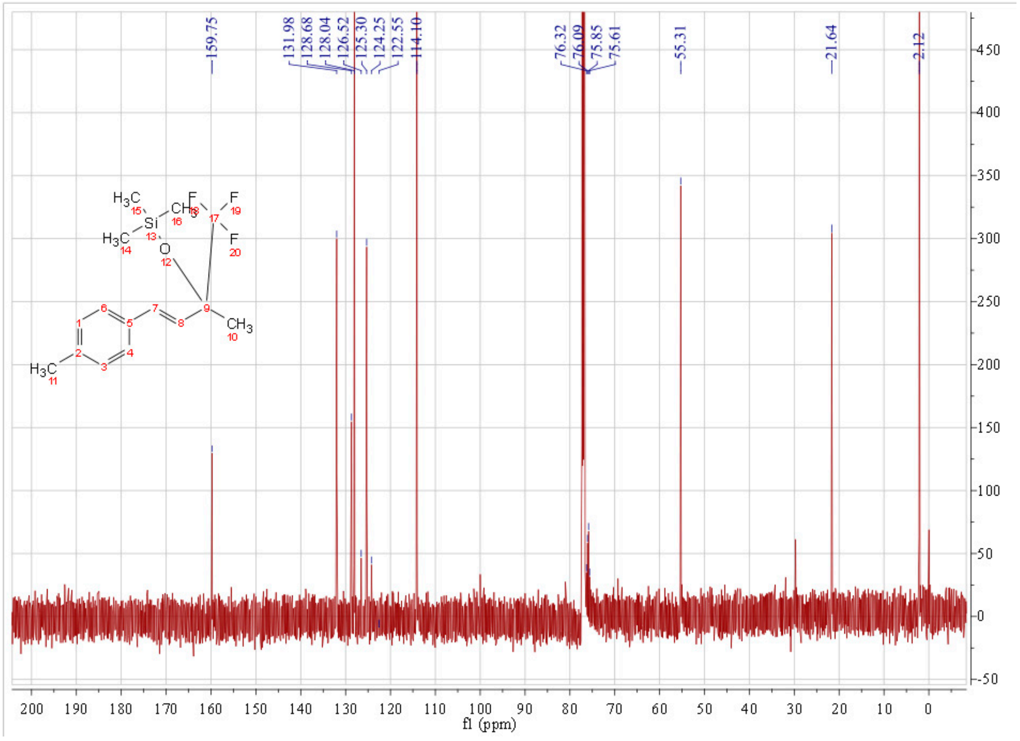

Figure S37 ^{1}H NMR spectra for compound 2s

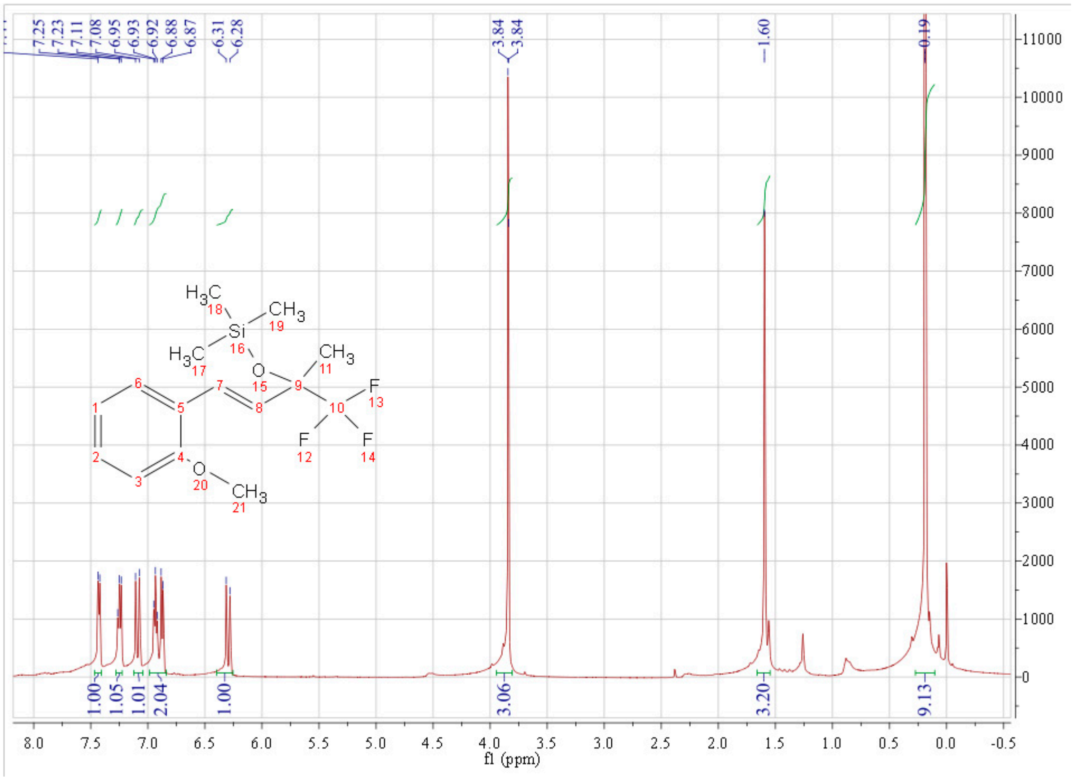

Figure S38 ^{13}C NMR spectra for compound 2s

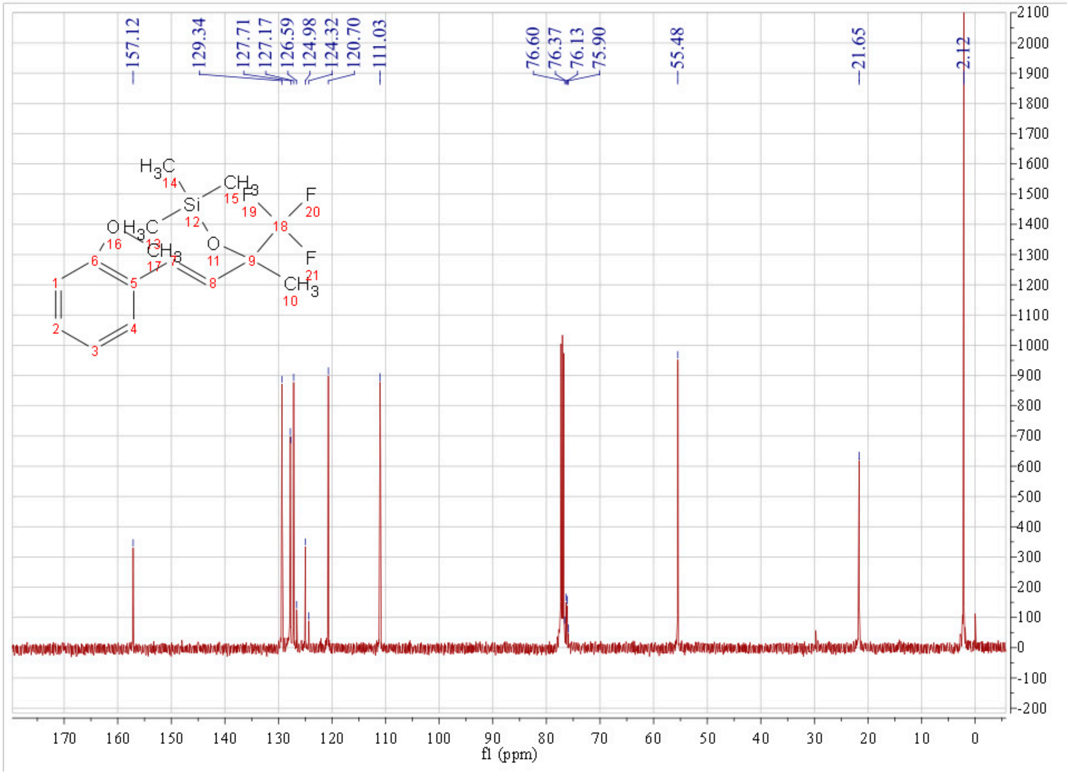

Figure S39 ^{1}H NMR spectra for compound 2t

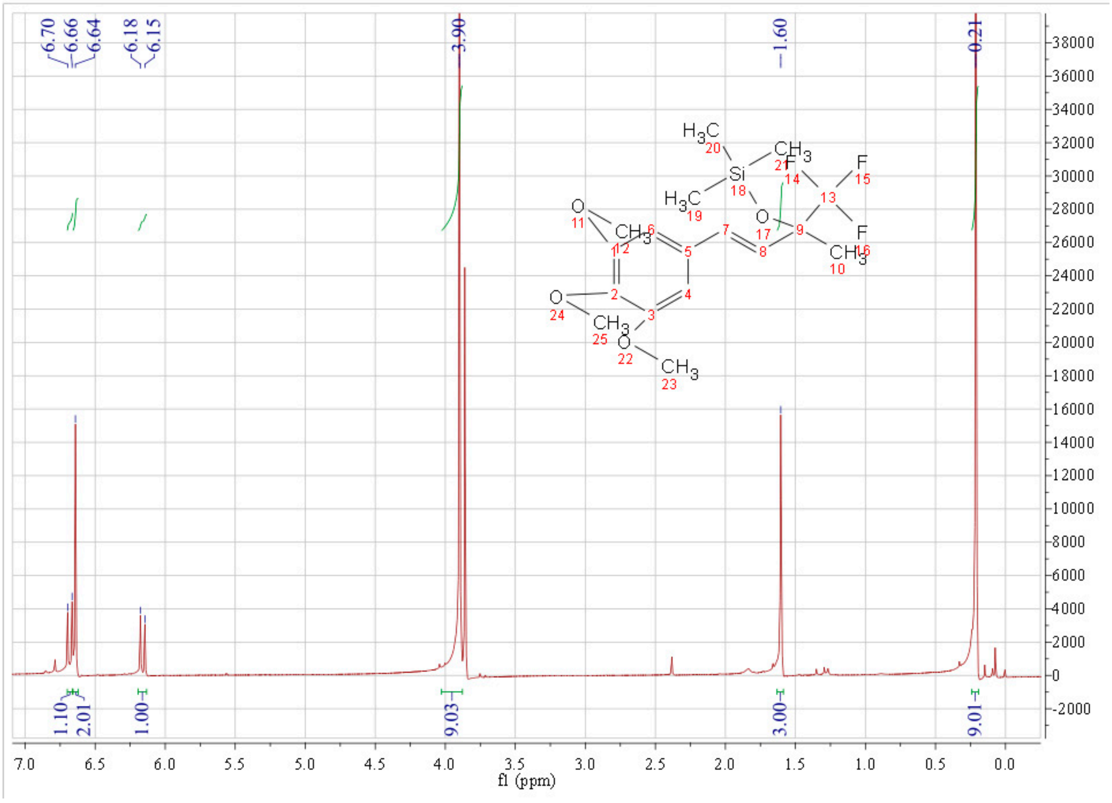

Figure S40 ^{13}C NMR spectra for compound 2t

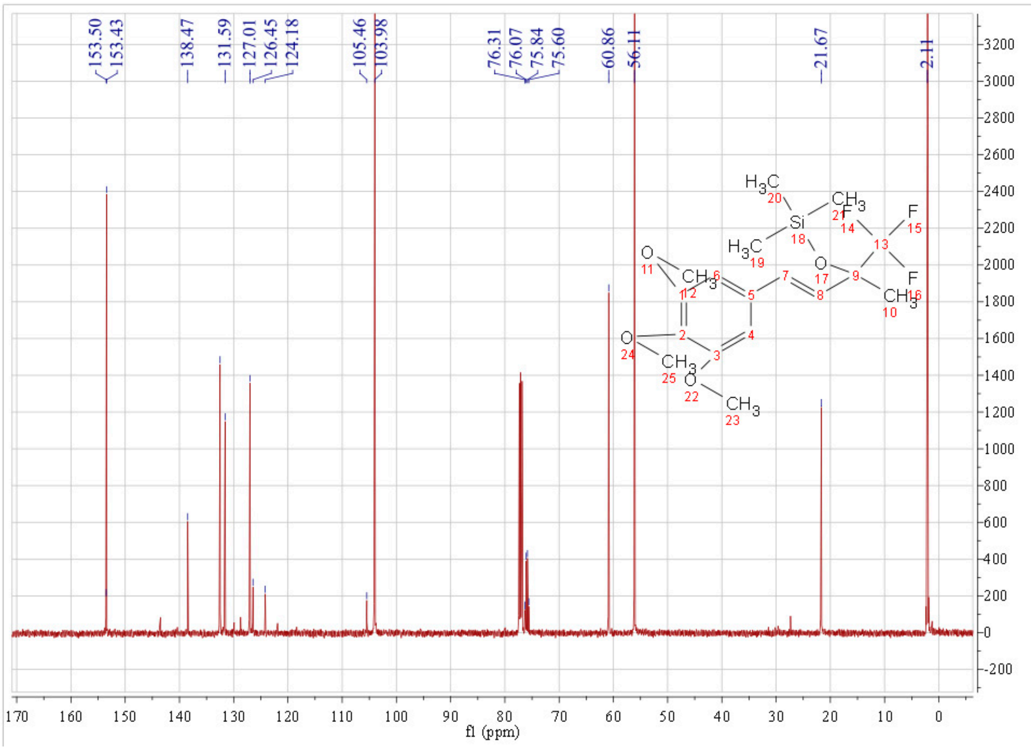

Figure S41 ^{1}H NMR spectra for compound 2u

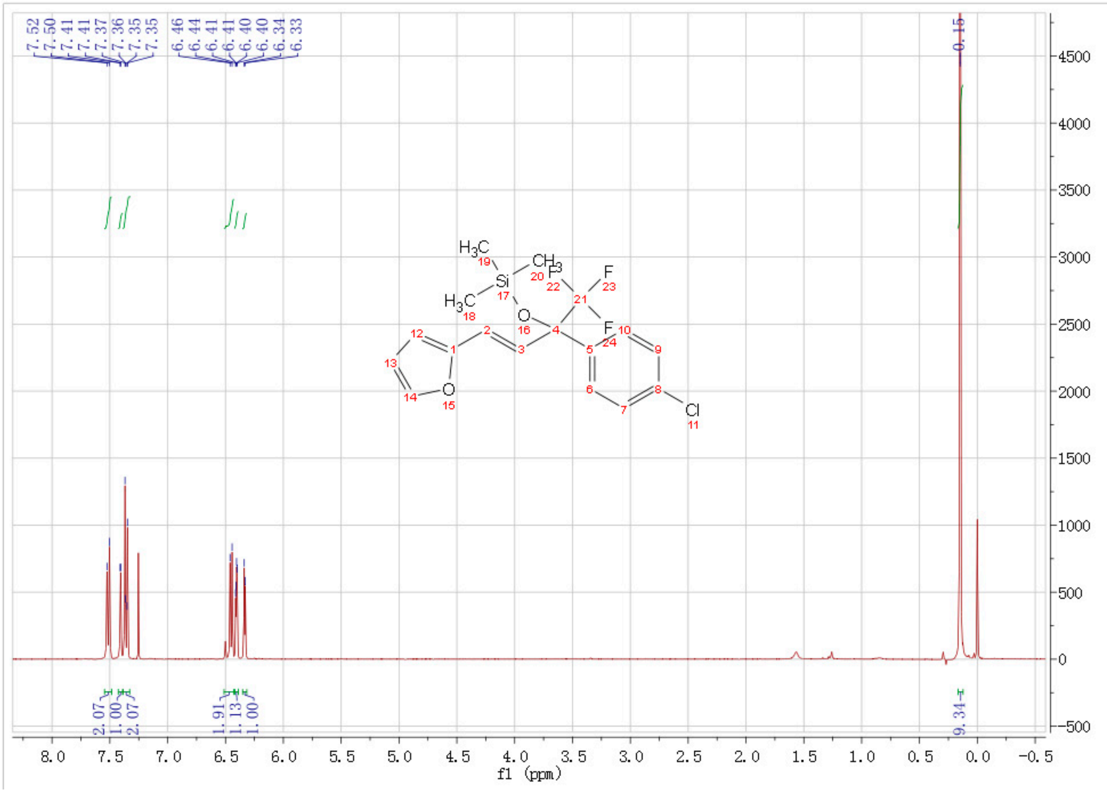

Figure S42 ^{13}C NMR spectra for compound 2u

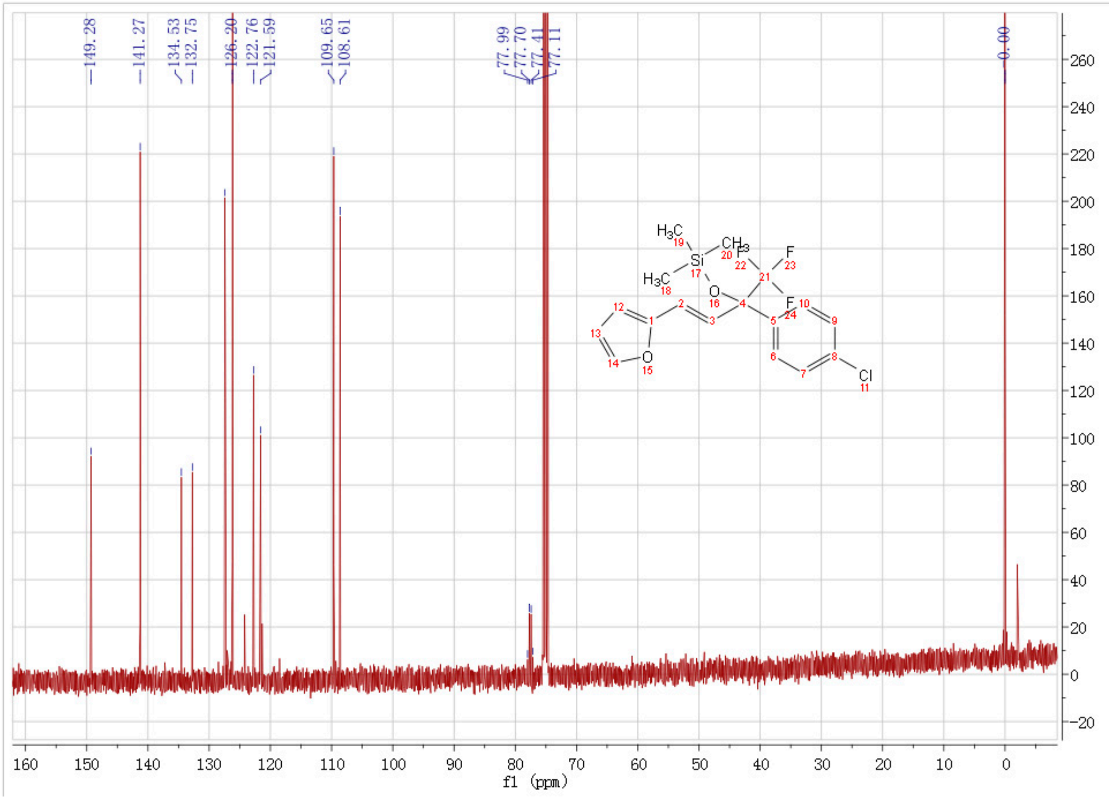

Figure S43 ^{1}H NMR spectra for compound 2v

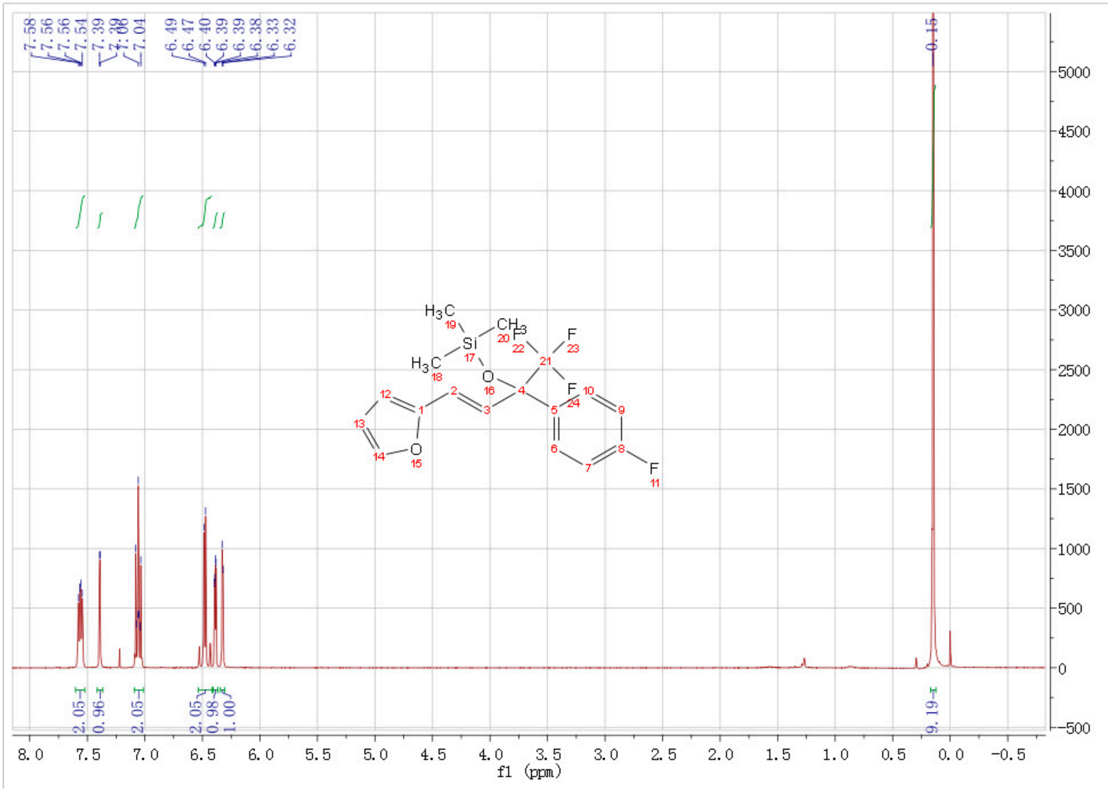

Figure S44 ^{13}C NMR spectra for compound 2v

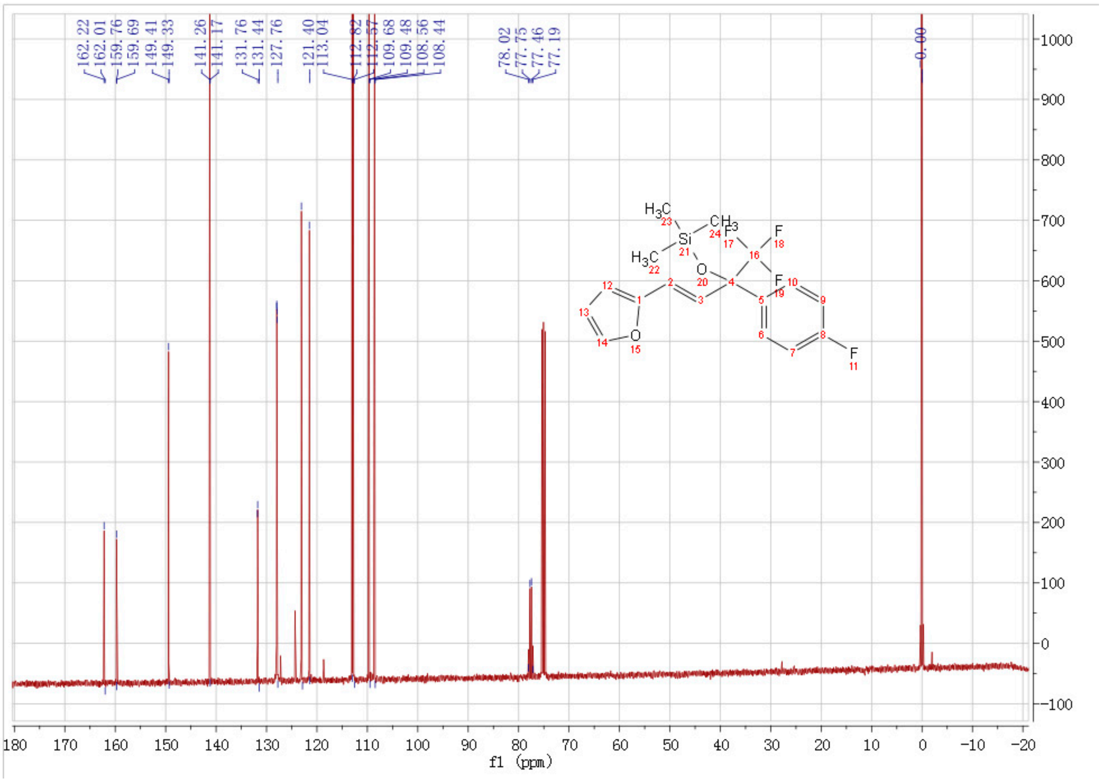

Figure S45 ^{1}H NMR spectra for compound 2w

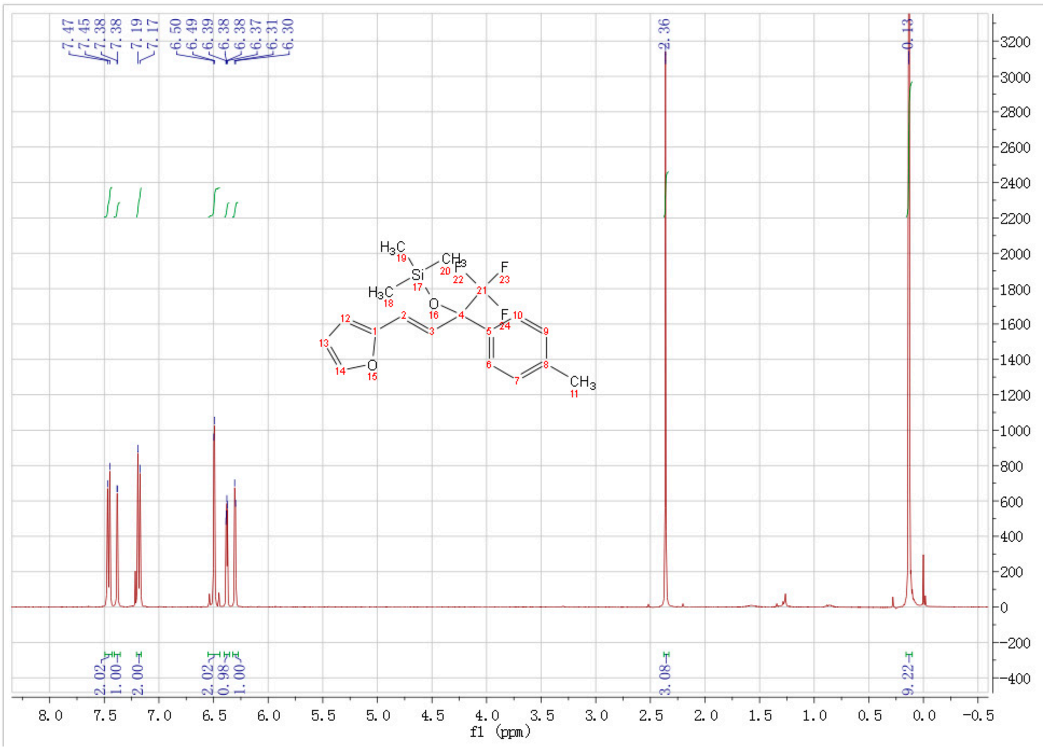

Figure S46 ^{13}C NMR spectra for compound 2w

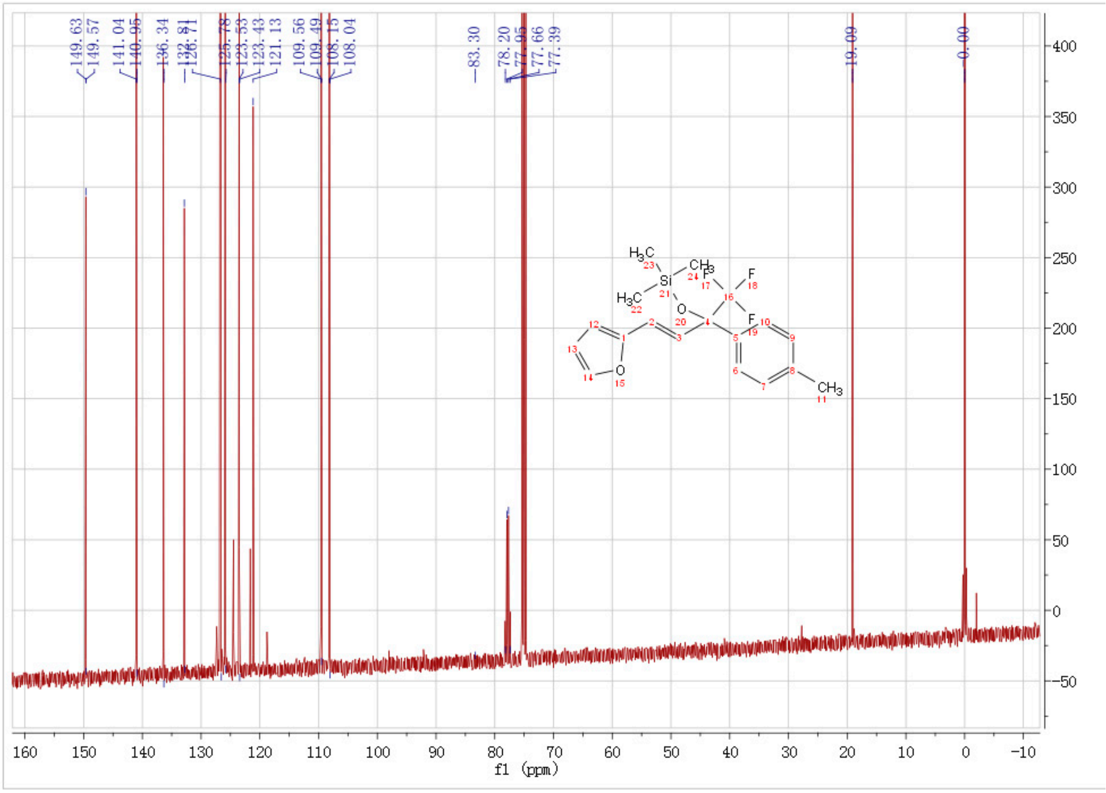

Figure S47 ^{1}H NMR spectra for compound 5a

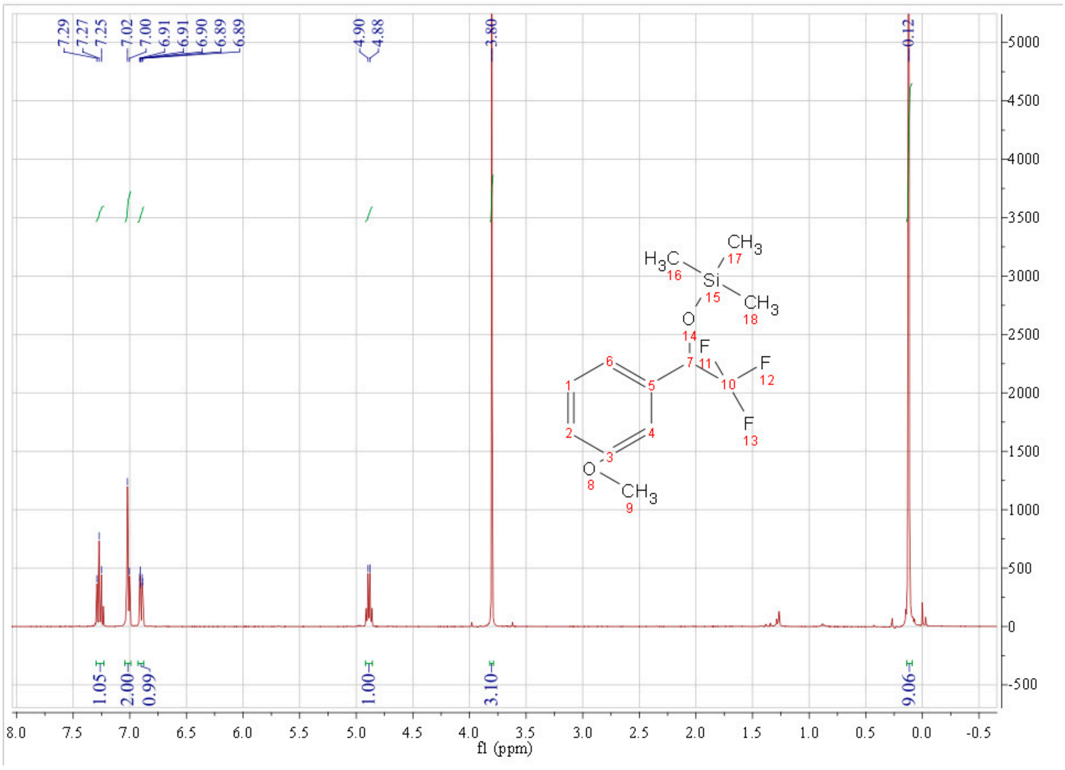

Figure S48 ^{13}C NMR spectra for compound 5a

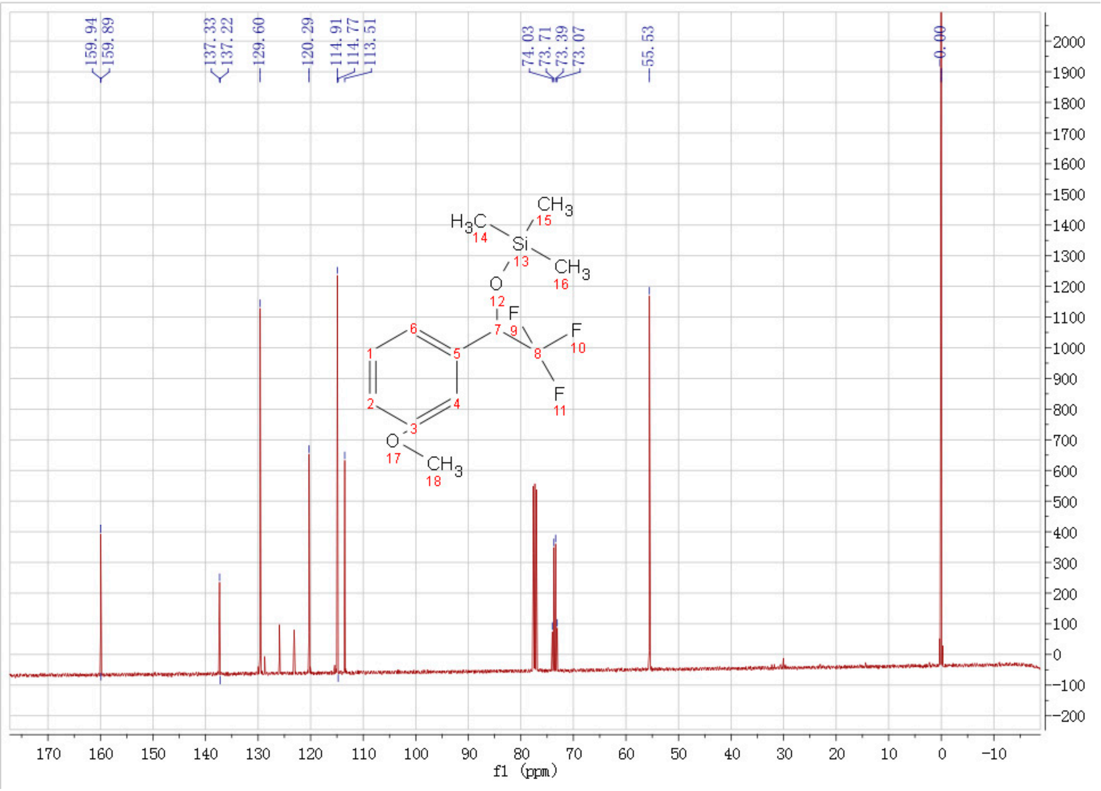

Figure S49 ^{1}H NMR spectra for compound 5b

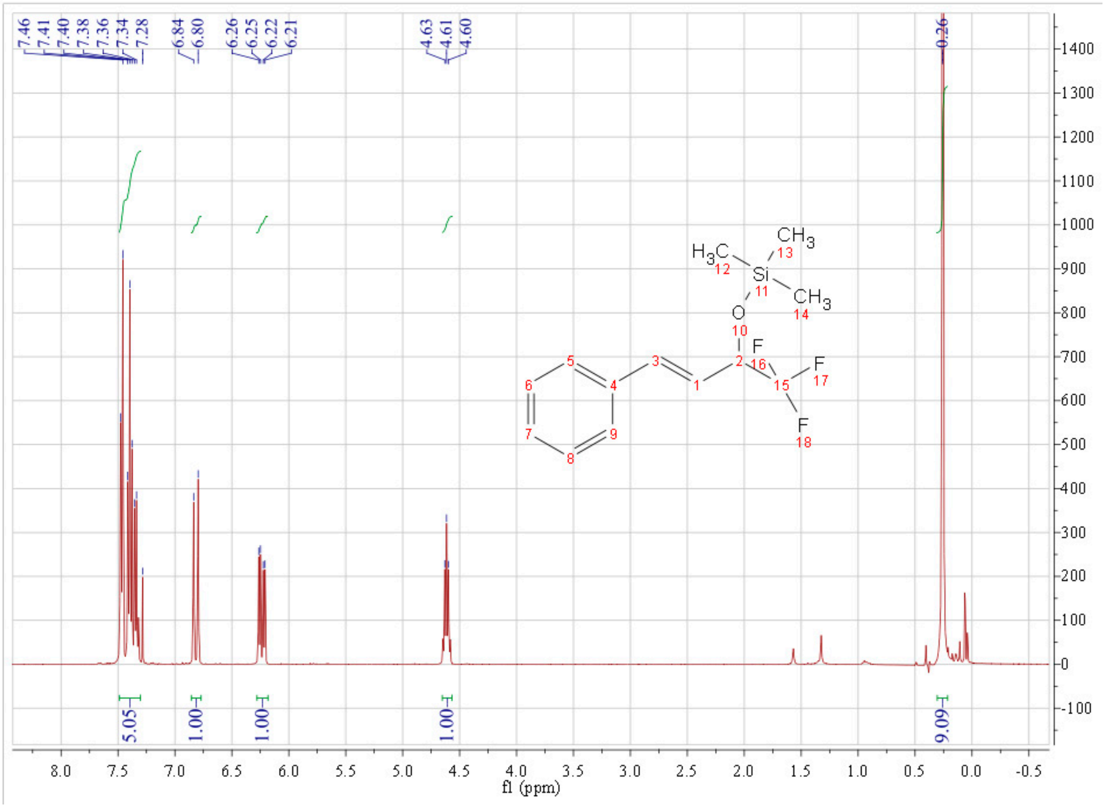

Figure S50 ^{13}C NMR spectra for compound 5b

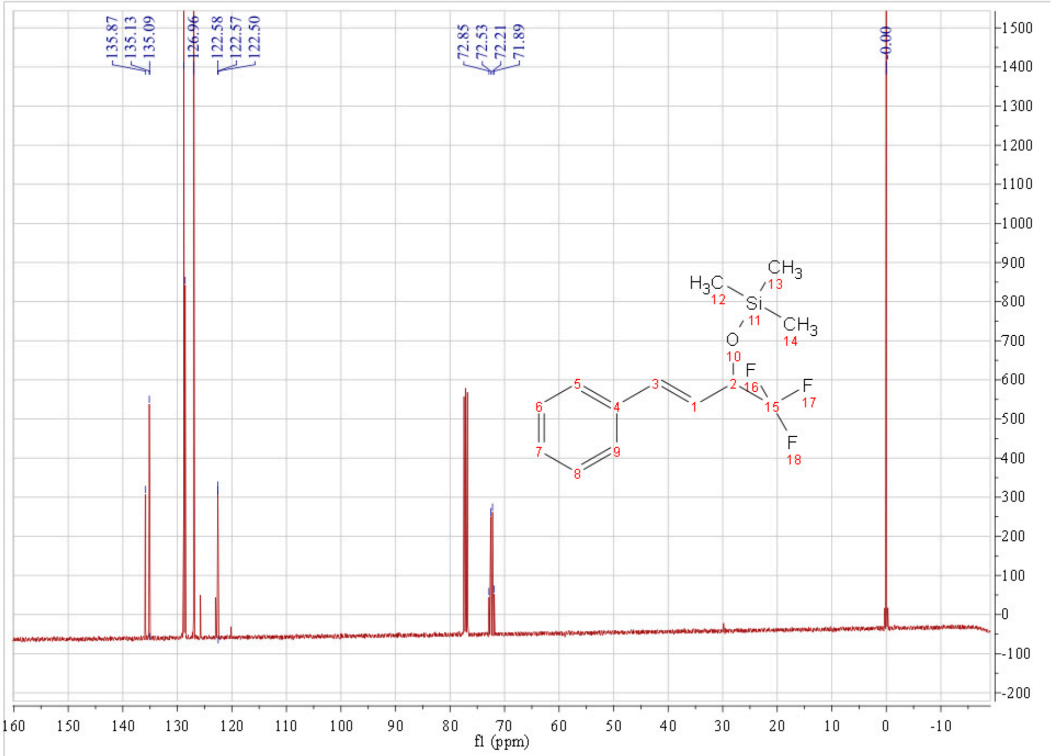

Figure S51 ^{1}H NMR spectra for compound 5c

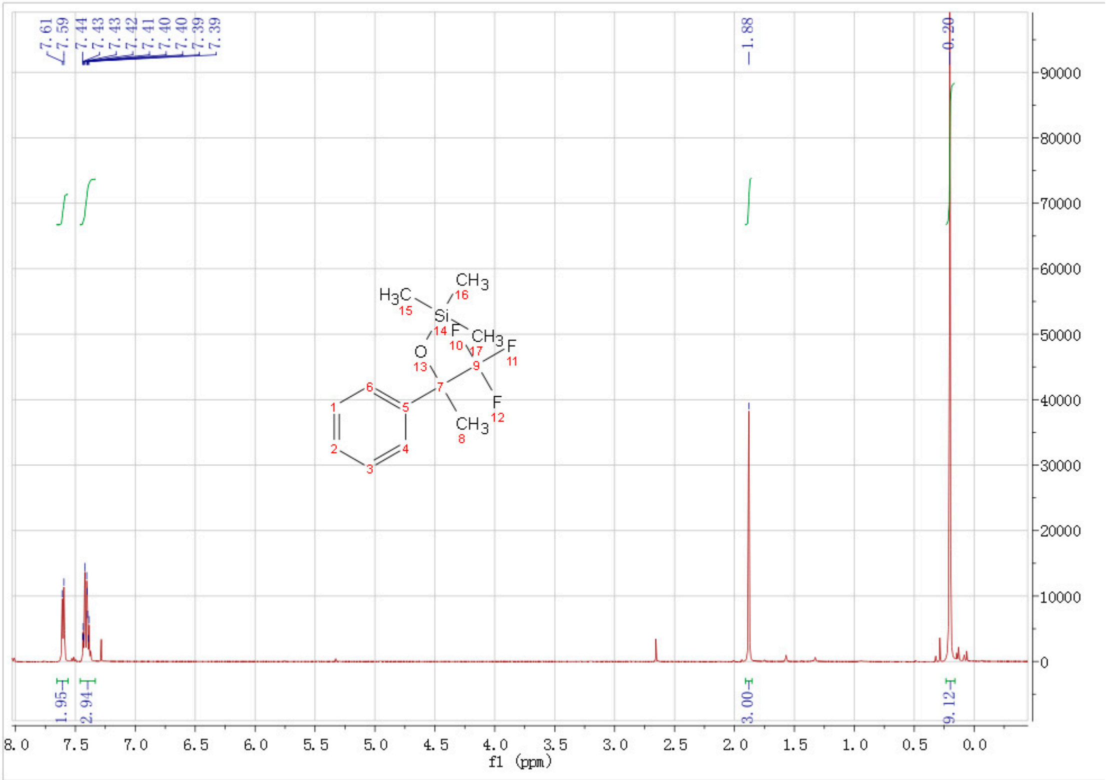

Figure S52 ^{13}C NMR spectra for compound 5c

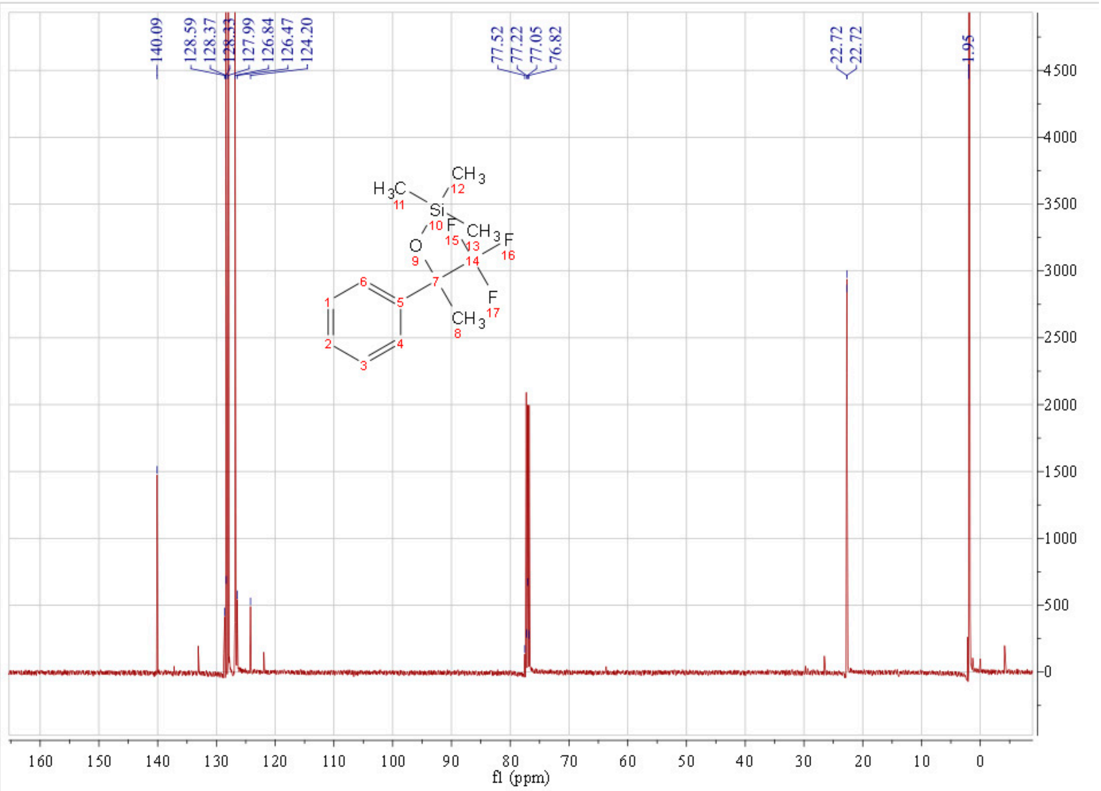

Figure S53 ^{1}H NMR spectra for compound 5d

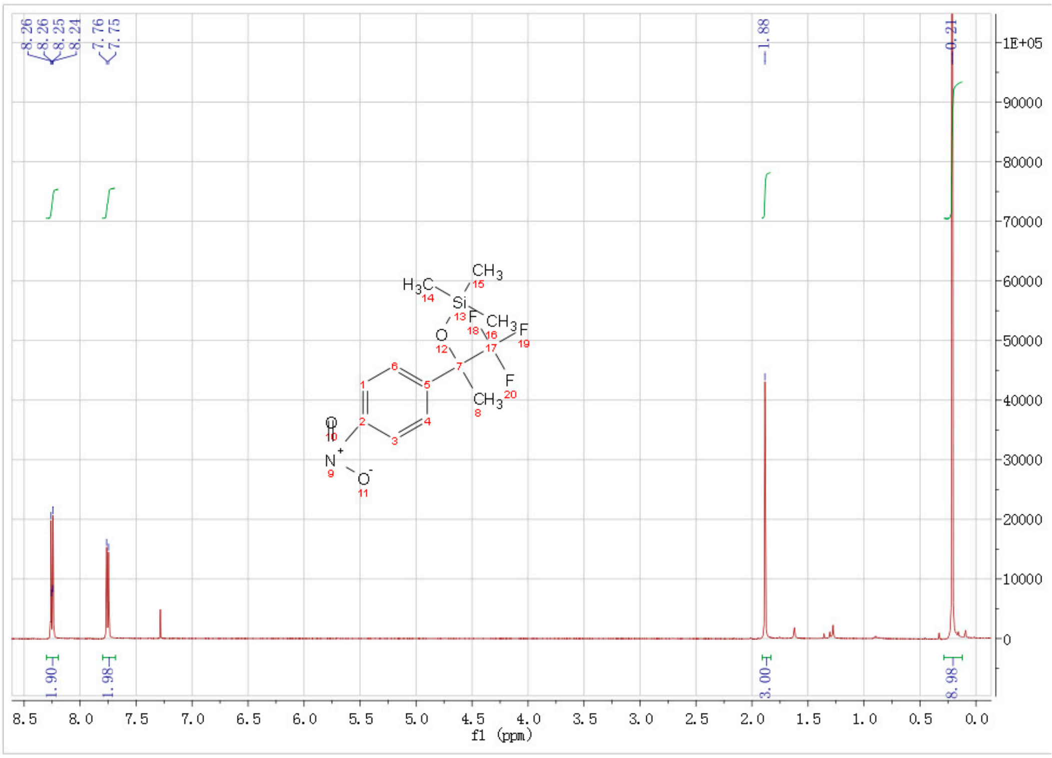

Figure S54 ^{13}C NMR spectra for compound 5d

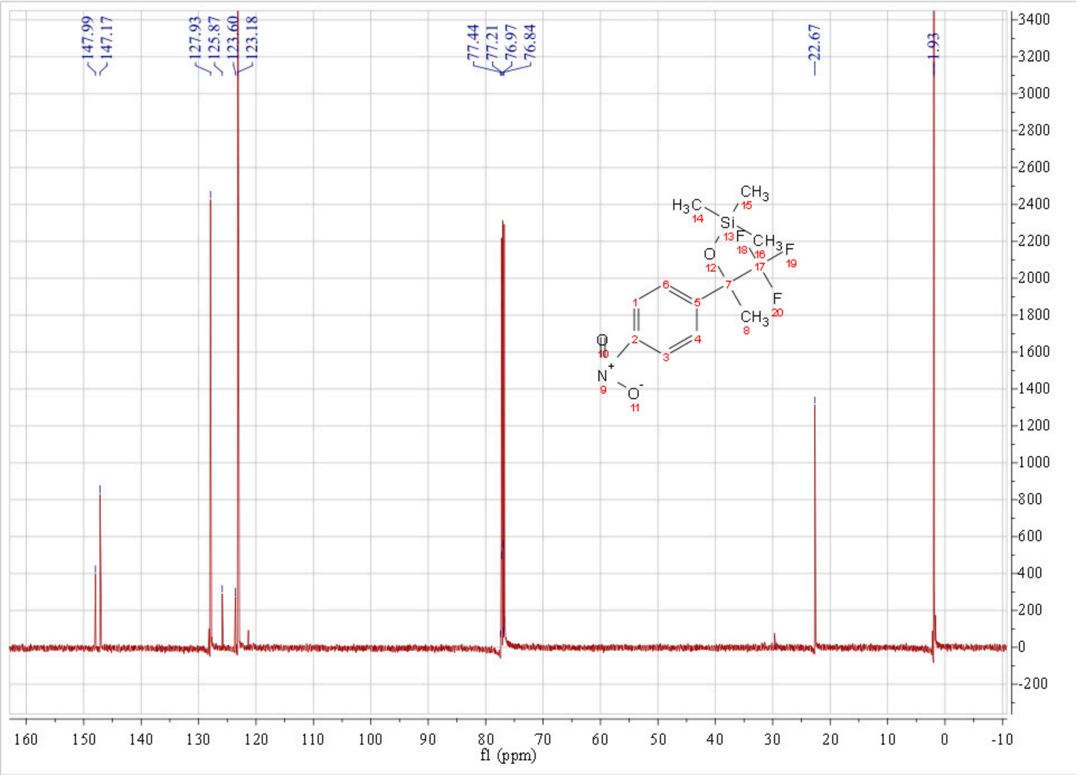

Figure S55 ^{1}H NMR spectra for compound 5e

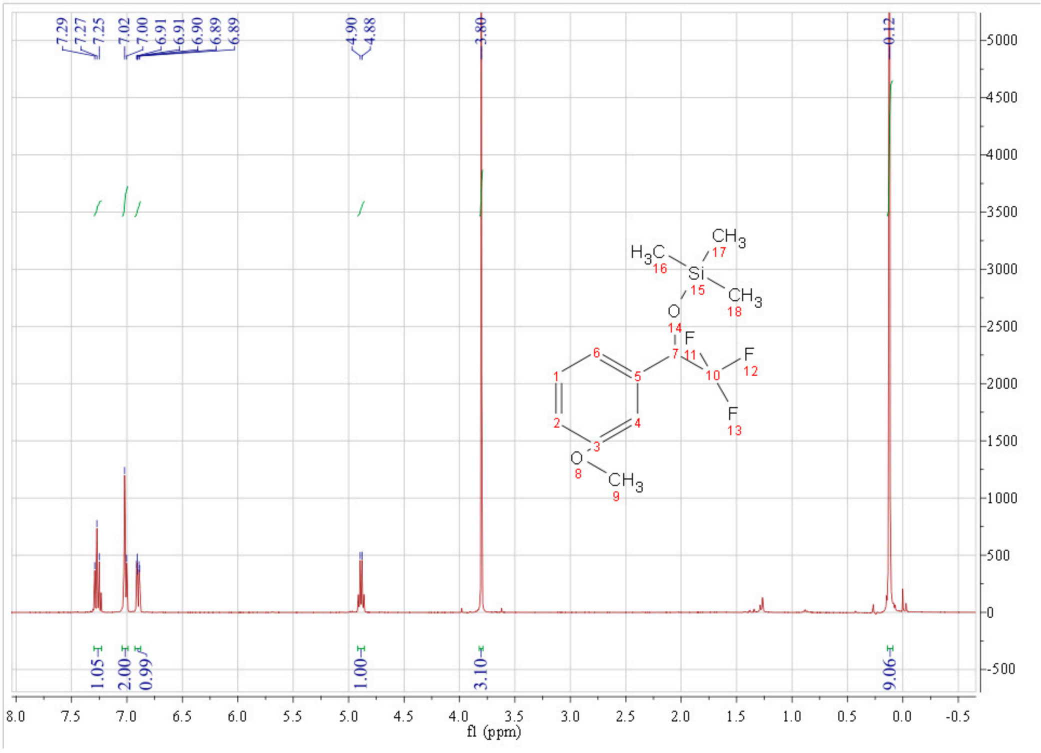

Figure S56 ^{13}C NMR spectra for compound 5e

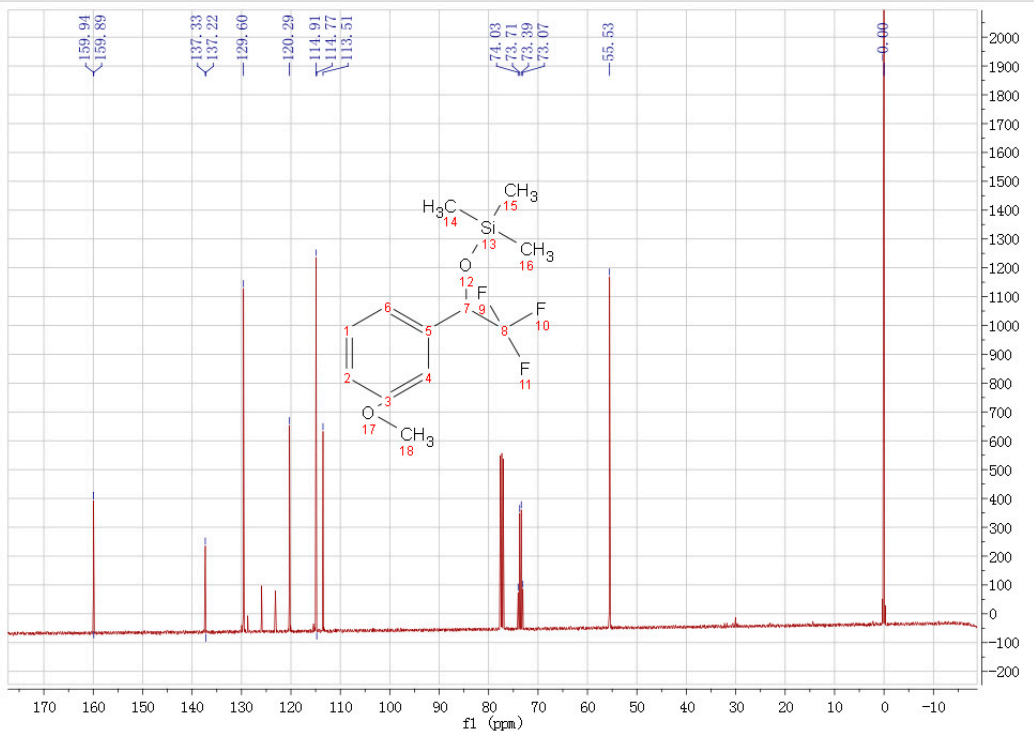

Figure S57 ^{1}H NMR spectra for compound 5f

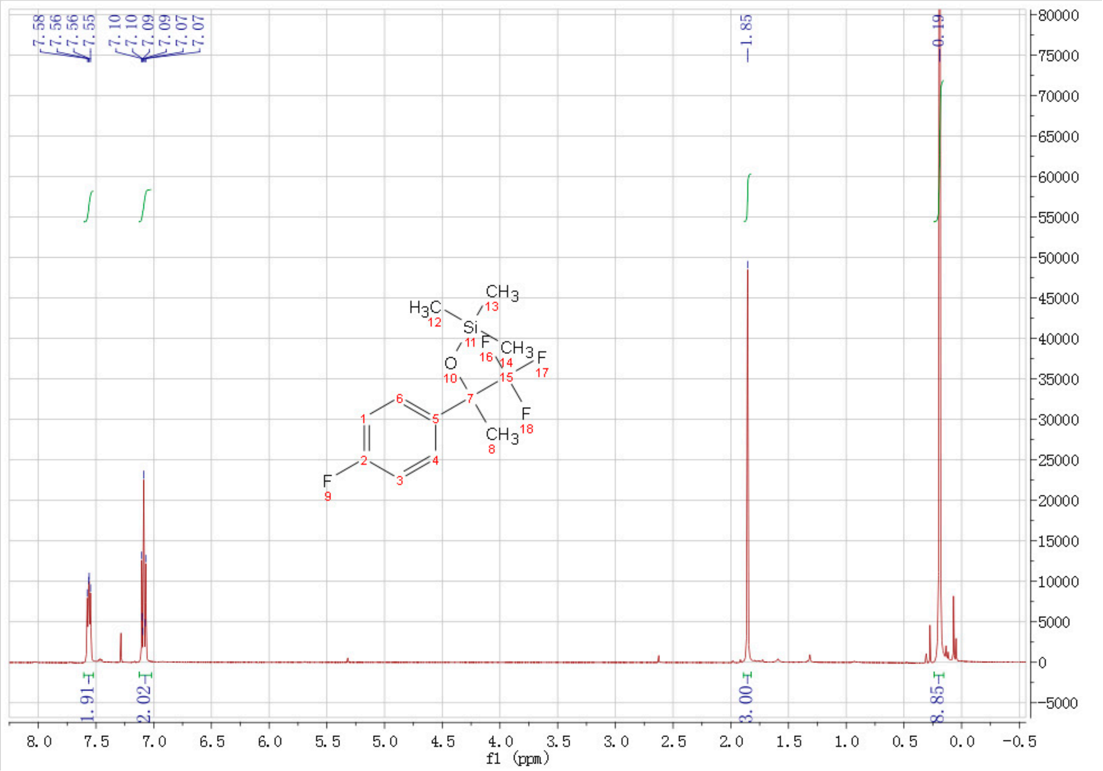

Figure S58 ^{13}C NMR spectra for compound 5f

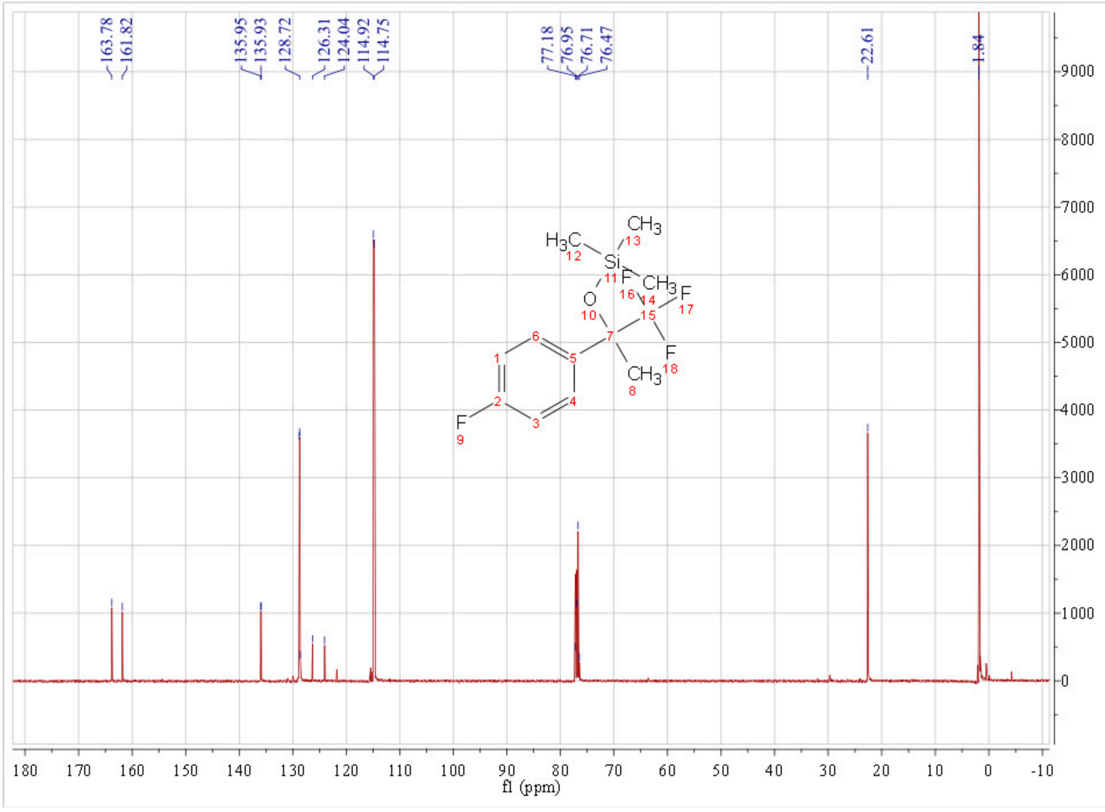

Figure S59 ^{1}H NMR spectra for compound 5g

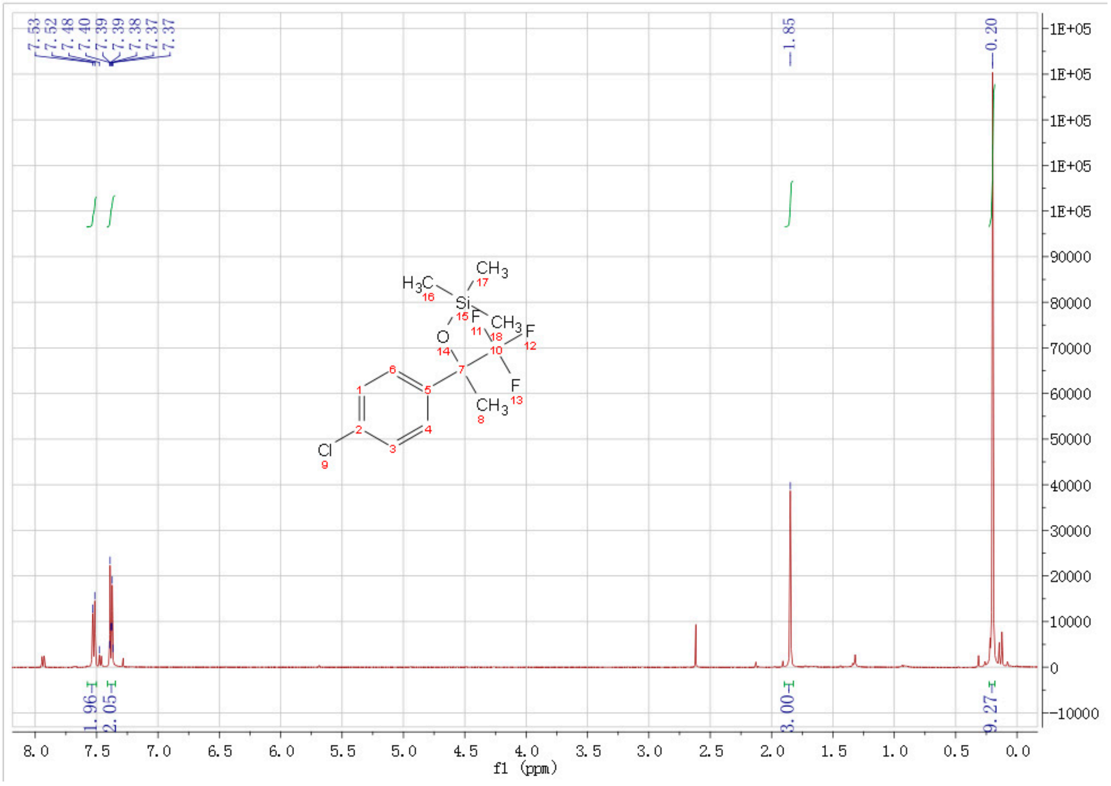

Figure S60 ^{13}C NMR spectra for compound 5g

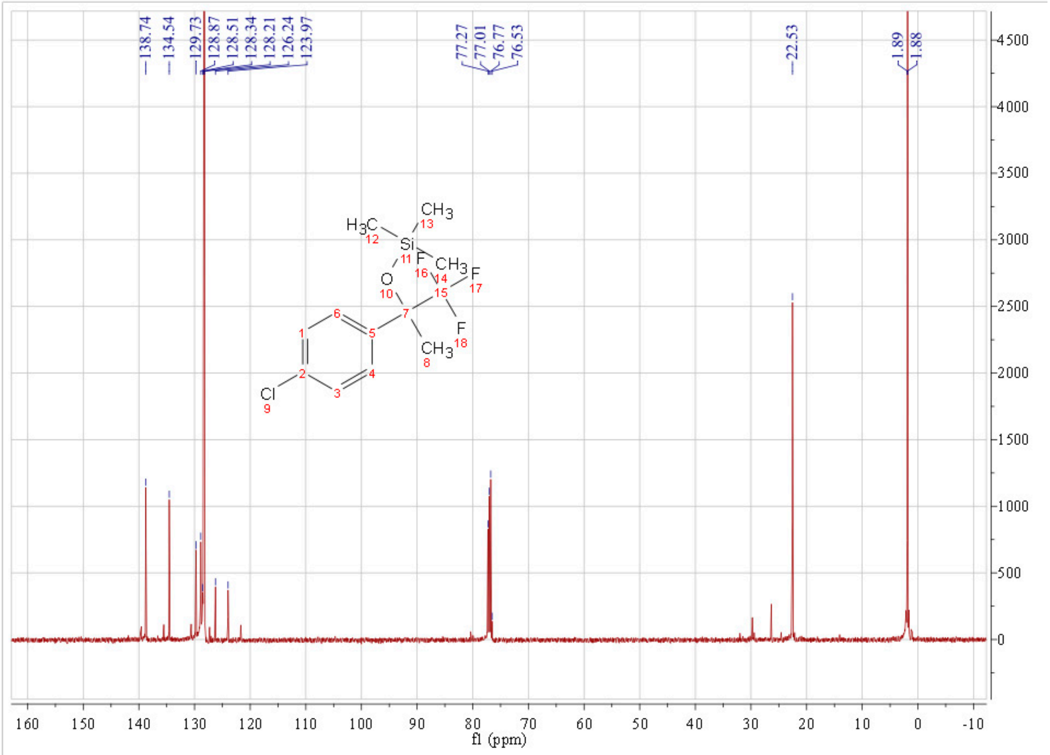

Figure S61 ^{1}H NMR spectra for compound 5h

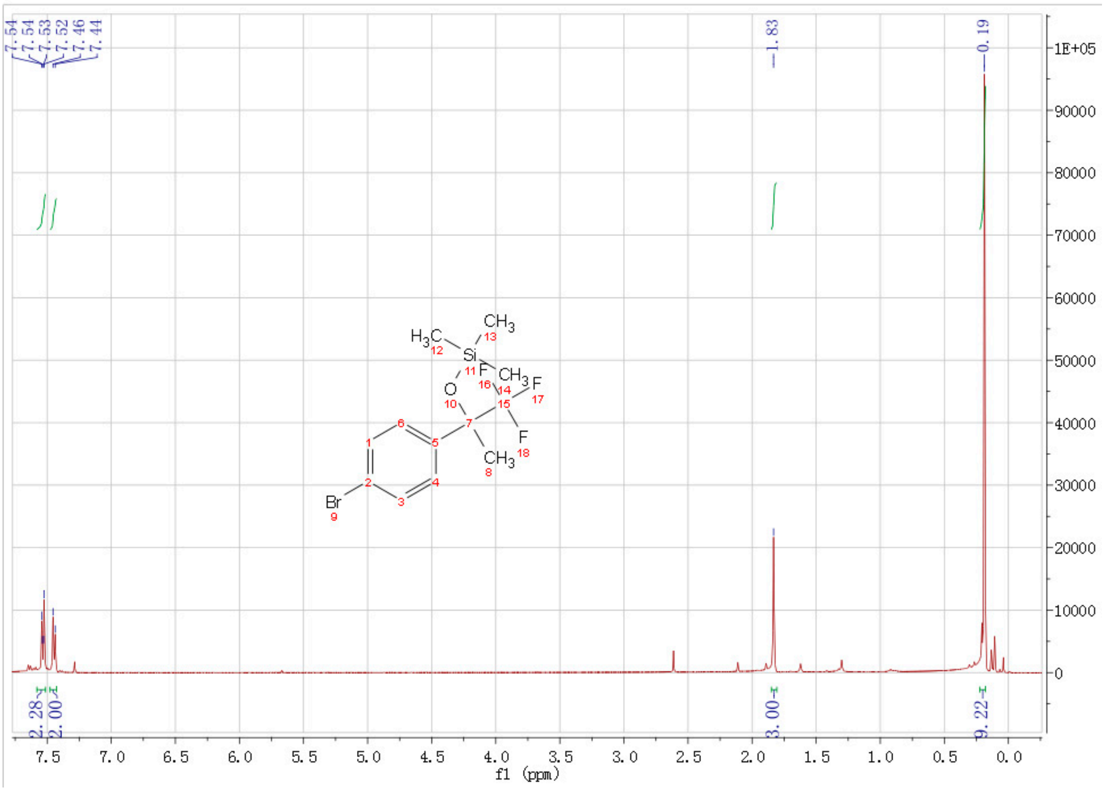

Figure S62 ^{13}C NMR spectra for compound 5h

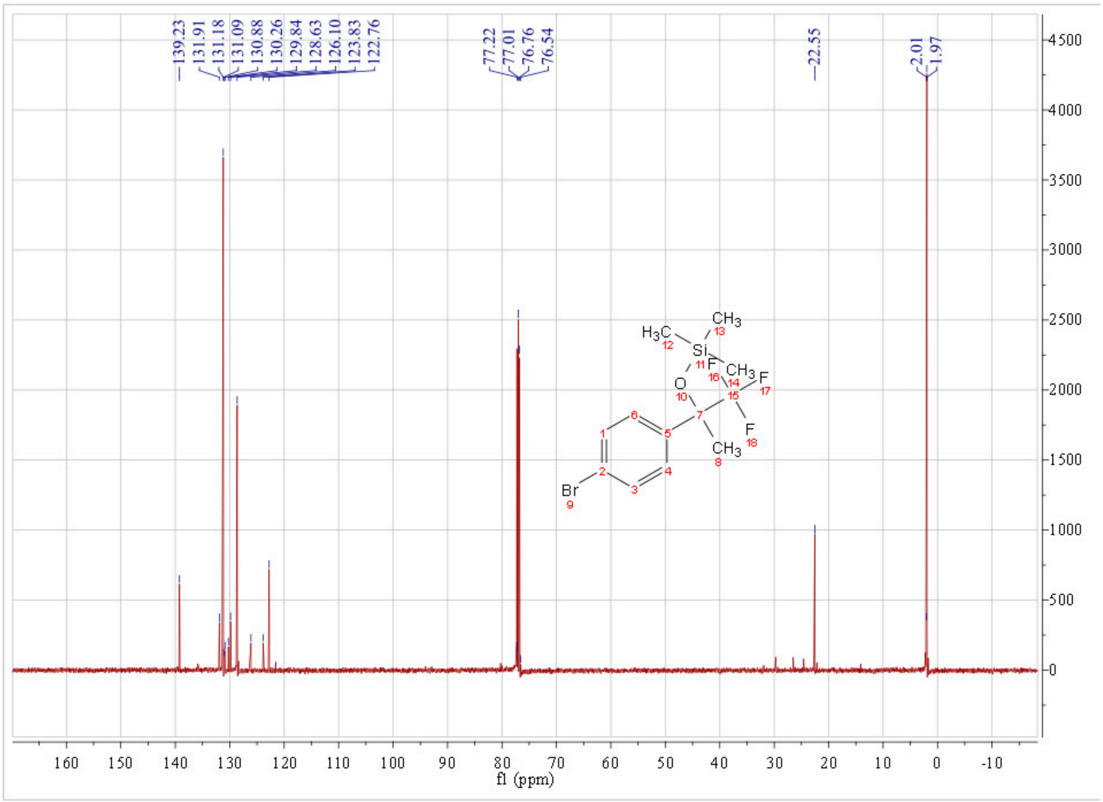

Figure S63 ^{1}H NMR spectra for compound 5i

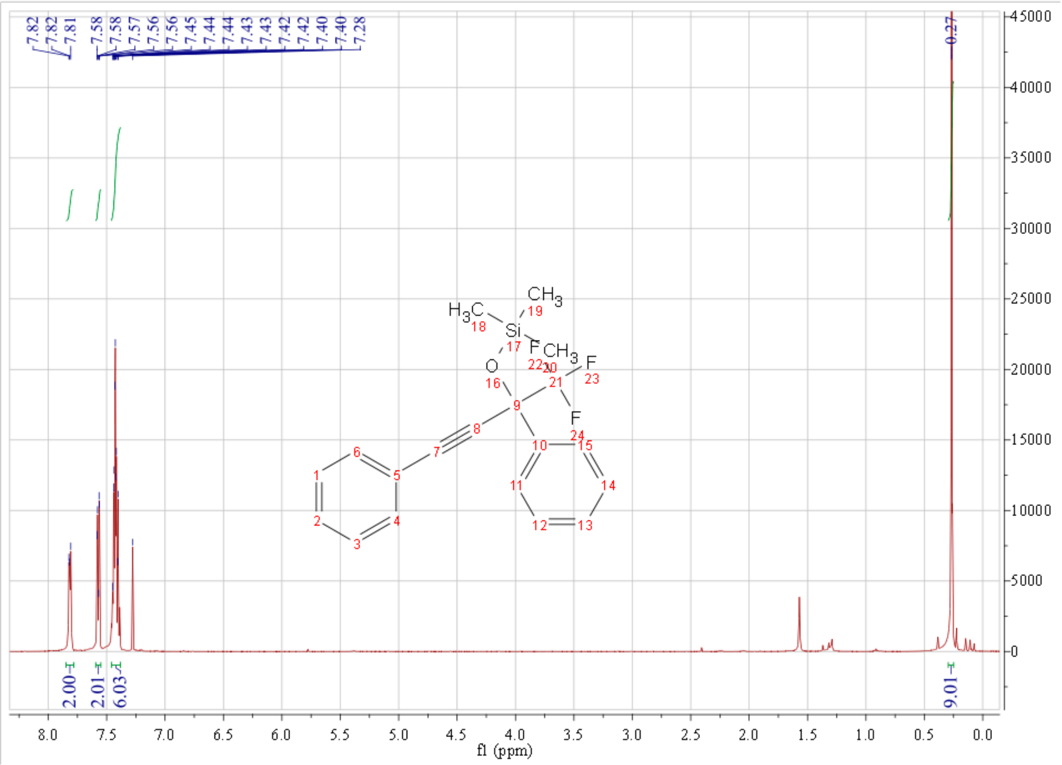

Figure S64 ^{13}C NMR spectra for compound 5i

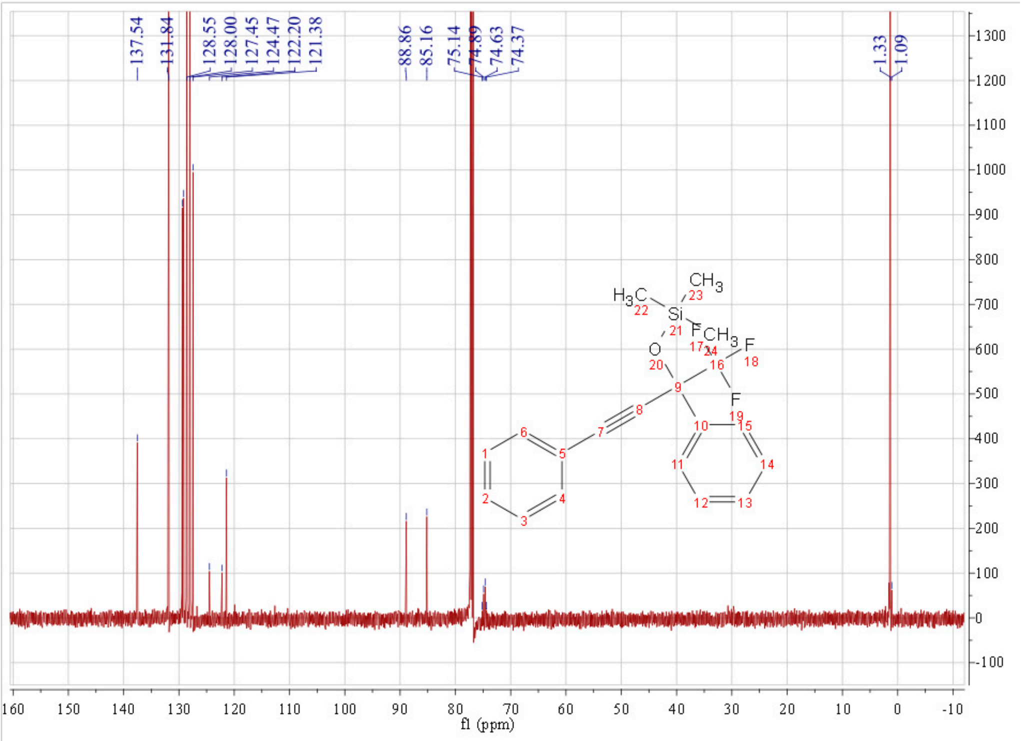

Figure S65 ¹H NMR spectra for compound 5j

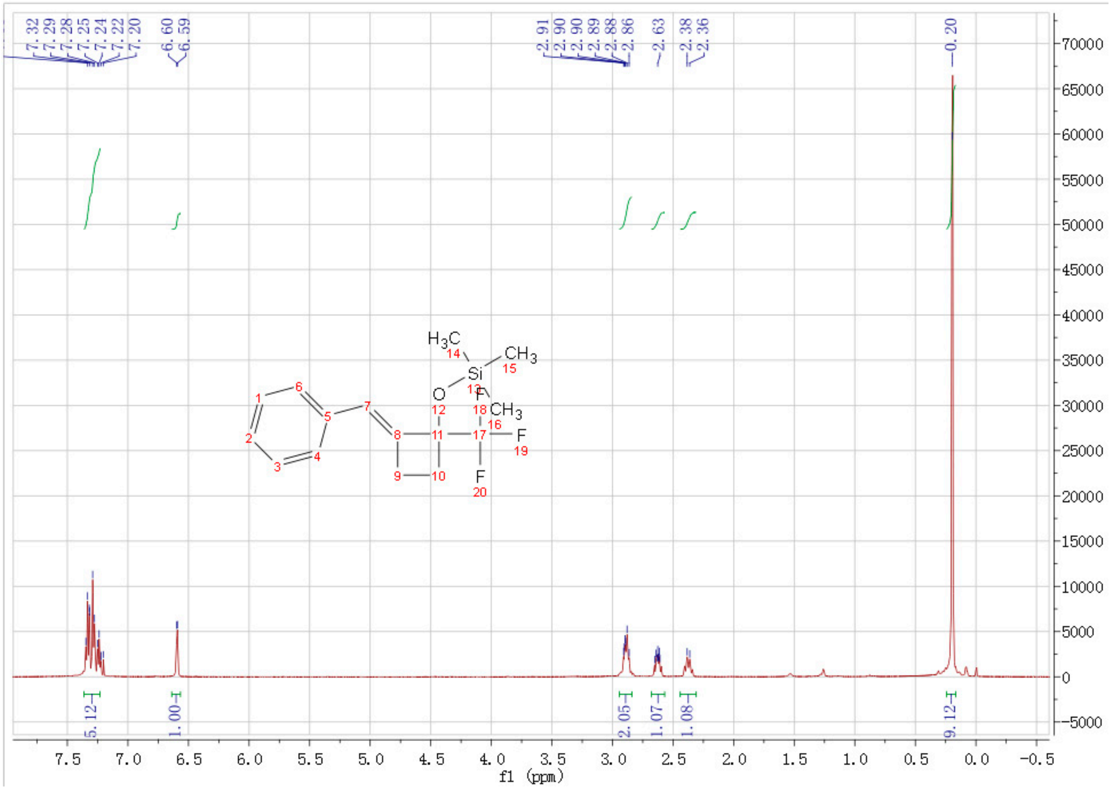

Figure S66 ¹³C NMR spectra for compound 5j

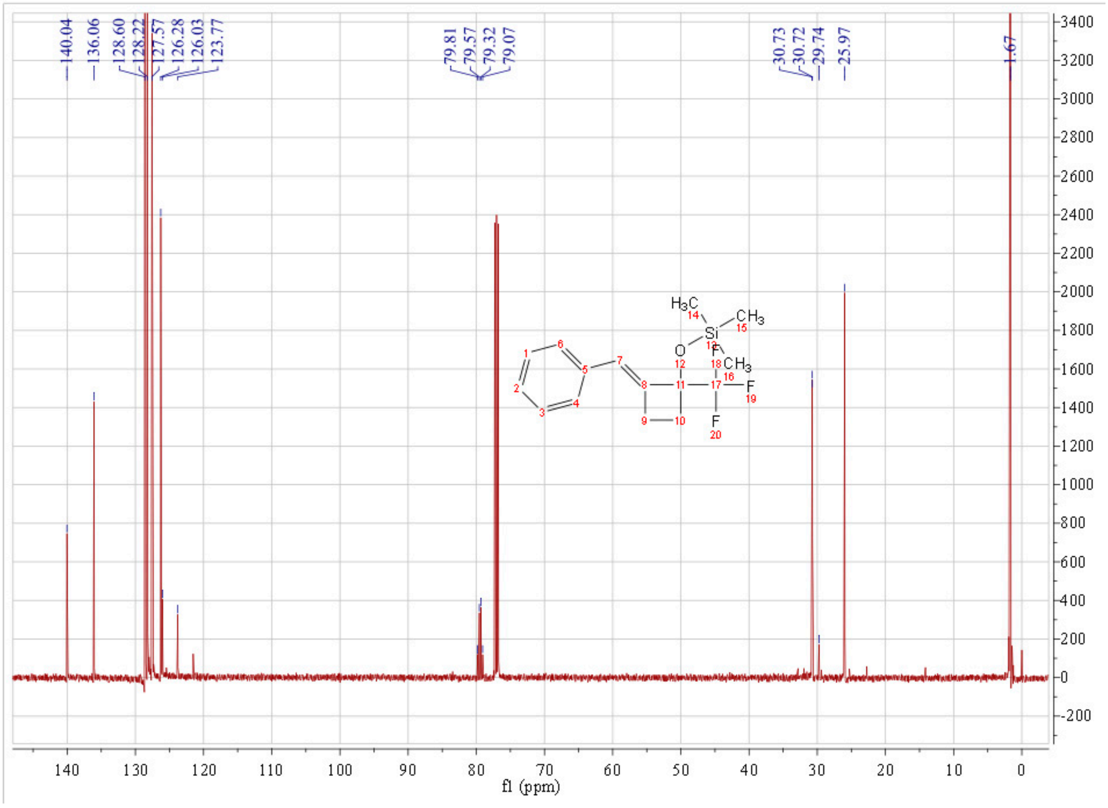

Figure S67 ^{1}H NMR spectra for compound 5k

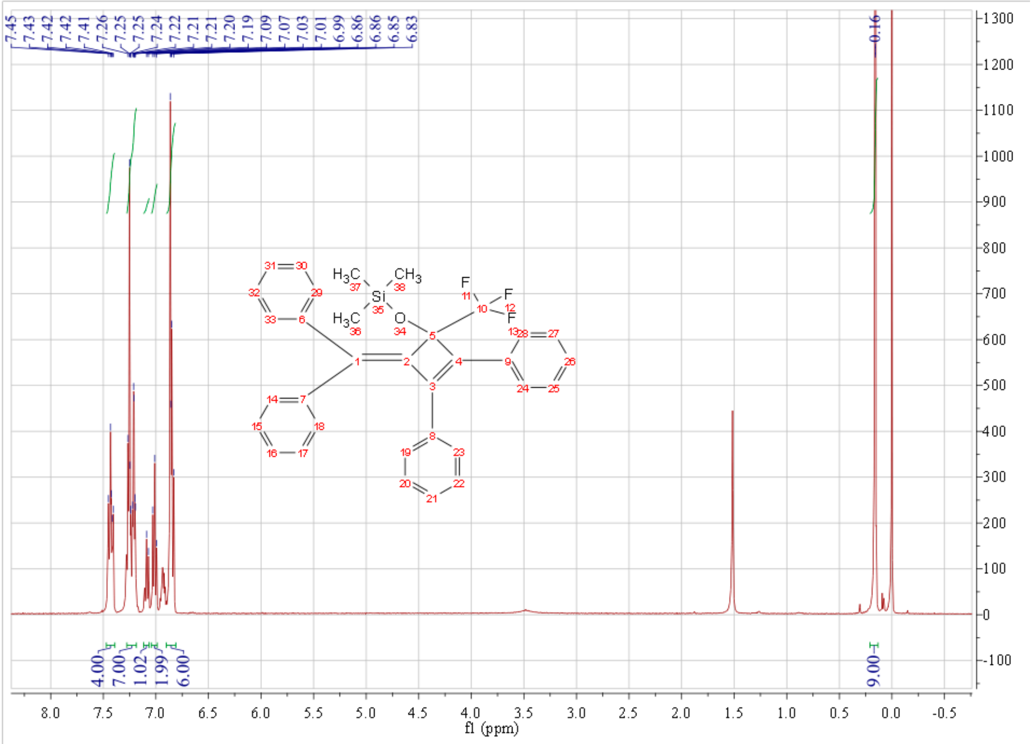

Figure S68 ^{13}C NMR spectra for compound 5k

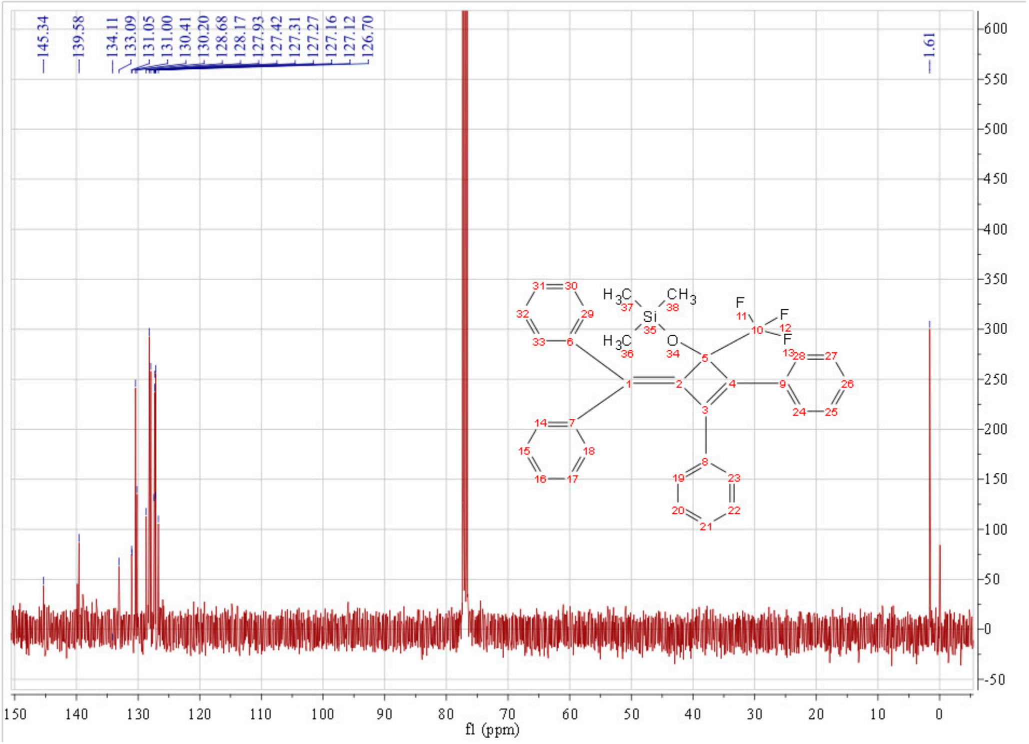

Supplement: Supplementary file 1 [file molecules-22-00769-s001.pdf]
